# Blood biomarkers for memory: toward early detection of risk for Alzheimer disease, pharmacogenomics, and repurposed drugs

**DOI:** 10.1038/s41380-019-0602-2

**Published:** 2019-12-02

**Authors:** A. B. Niculescu, H. Le-Niculescu, K. Roseberry, S. Wang, J. Hart, A. Kaur, H. Robertson, T. Jones, A. Strasburger, A. Williams, S. M. Kurian, B. Lamb, A. Shekhar, D. K. Lahiri, A. J. Saykin

**Affiliations:** 1 Department of Psychiatry, Indiana University School of Medicine, Indianapolis, IN USA; 2 Stark Neuroscience Research Institute, Indiana University School of Medicine, Indianapolis, IN USA; 3 Indianapolis VA Medical Center, Indianapolis, IN USA; 4 Indiana Alzheimer Disease Center, Indiana University School of Medicine, Indianapolis, IN USA; 5 Department of Molecular Medicine, Scripps Research, La Jolla, CA USA

**Keywords:** Genetics, Predictive markers

## Abstract

Short-term memory dysfunction is a key early feature of Alzheimer’s disease (AD). Psychiatric patients may be at higher risk for memory dysfunction and subsequent AD due to the negative effects of stress and depression on the brain. We carried out longitudinal within-subject studies in male and female psychiatric patients to discover blood gene expression biomarkers that track short term memory as measured by the retention measure in the Hopkins Verbal Learning Test. These biomarkers were subsequently prioritized with a convergent functional genomics approach using previous evidence in the field implicating them in AD. The top candidate biomarkers were then tested in an independent cohort for ability to predict state short-term memory, and trait future positive neuropsychological testing for cognitive impairment. The best overall evidence was for a series of new, as well as some previously known genes, which are now newly shown to have functional evidence in humans as blood biomarkers: RAB7A, NPC2, TGFB1, GAP43, ARSB, PER1, GUSB, and MAPT. Additional top blood biomarkers include GSK3B, PTGS2, APOE, BACE1, PSEN1, and TREM2, well known genes implicated in AD by previous brain and genetic studies, in humans and animal models, which serve as reassuring de facto positive controls for our whole-genome gene expression discovery approach. Biological pathway analyses implicate LXR/RXR activation, neuroinflammation, atherosclerosis signaling, and amyloid processing. Co-directionality of expression data provide new mechanistic insights that are consistent with a compensatory/scarring scenario for brain pathological changes. A majority of top biomarkers also have evidence for involvement in other psychiatric disorders, particularly stress, providing a molecular basis for clinical co-morbidity and for stress as an early precipitant/risk factor. Some of them are modulated by existing drugs, such as antidepressants, lithium and omega-3 fatty acids. Other drug and nutraceutical leads were identified through bioinformatic drug repurposing analyses (such as pioglitazone, levonorgestrel, salsolidine, ginkgolide A, and icariin). Our work contributes to the overall pathophysiological understanding of memory disorders and AD. It also opens new avenues for precision medicine- diagnostics (assement of risk) as well as early treatment (pharmacogenomically informed, personalized, and preventive).

## Introduction

“If you find ways to repair the memory damaged by Alzheimer’s disease or dementia and so forth, it is very likely that the same methods could be used to upgrade the memory of completely healthy people”

- Yuval Noah Harari

Alzheimer disease (AD) is a clear and present danger to older adults, and has a profound socio-economic impact. Existing therapies are limited in efficacy. Early identification of individuals at risk may open the door to preventive approaches. Short-term memory dysfunction is a key early feature of AD. We proposed to identify blood biomarkers that track a relevant related quantitative phenotype for short-term memory, the retention measure of recall in Hopkins Verbal Learning Test (HVLT) (Fig. S1). Blood biomarkers are easily accessible clinically, as opposed to brain imaging or even CSF changes. The nervous and immune system have some common developmental roots, and there are bi-directional brain-immune interactions [1]. Similar gene expression changes in brain and blood cells may also be due to similar gene promoter regulation in response to shared internal milieu and external environment changes [2–6]. Previous work by our group has identified blood gene expression biomarkers that track suicidal ideation using longitudinal within-subject designs, validated them in suicide completers, and tested them in independent cohorts for ability to assess state (suicidal ideation), and ability to predict trait (future hospitalizations for suicidality) [7–10]. Those biomarkers were also useful in pharmacogenomic and drug repurposing analyses. We recently successfully used a similar approach for pain [11], and for stress [12]. In the current study, we endeavored to use this comprehensive step-wise approach to identify biomarkers for short-term memory that may have relevance to AD. We conducted our longitudinal studies in psychiatric disorder subjects, a population enriched in memory retention abnormalities, and which may be at increased risk of AD and other aging-related disorders, due at least in part to the effects of stress and depression [13, 14]. The subjects had blood gene expression data at multiple testing visits, and were phenotyped at each visit, including with HVLT. They also had electronic medical records for long term follow-up of subsequent outcomes, including future neuropsychological testing as part of standard clinical care.

## Materials and methods

### Cohorts

**Fig. 1:**
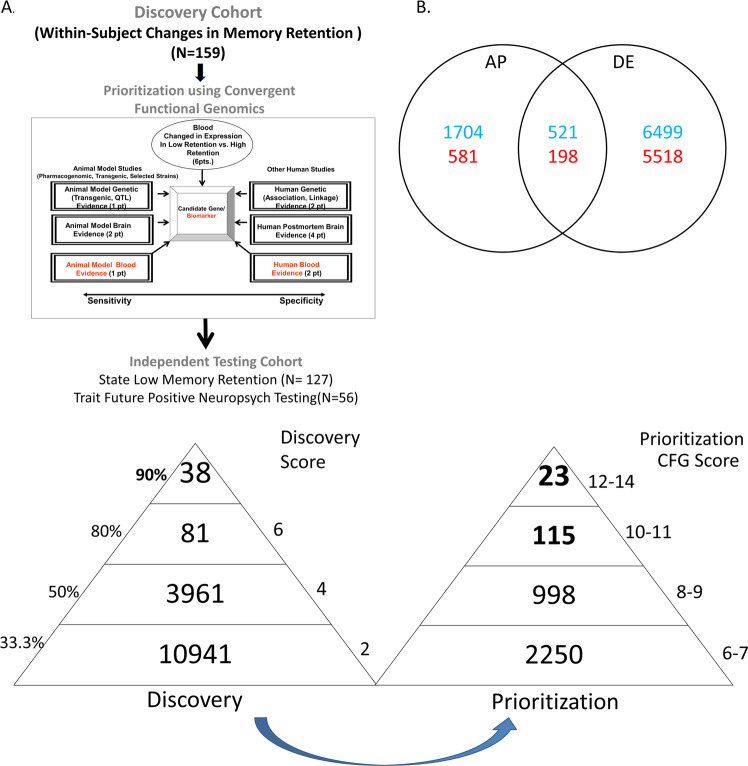
Steps 1-3: discovery, prioritization and validation/testing. **a** Cohorts used in study, depicting flow of discovery, prioritization, and testing of biomarkers. **b** Differential gene expression in the discovery cohort -number of genes identified with differential expression (DE) and absent–present (AP) methods with an internal score of 2 and above. Red—increased in expression in high memory, blue—decreased in expression in high memory. Pyramid on the left depicts the number of discovery step probesets, identified based on their score for tracking memory, with a maximum of internal points of 6 (33% (2 pt), 50% (4 pt) and 80% (6 pt)). Pyramid on the right depicts prioritization with CFG for prior evidence of involvement in AD. In the prioritization step probesets are converted to their associated genes using Affymetrix annotation and GeneCards. Genes are prioritized and scored using CFG for AD evidence with a maximum of 12 external points. Genes scoring at least ten points out of a maximum possible of 18 total internal and external scores points are carried to the testing step

We used two independent cohorts of psychiatric disorders patients, one for Discovery of candidate biomarkers, and one for validation/testing of top biomarkers (for predicting memory state, and predicting future positive neuropsychological testing for cognitive impairment) (Fig. 1, Table 1, and Table S1).

**Table 1:** Aggregate demographics.

Cohorts	Number of subjects (number of visits)	Gender	Diagnosis	Ethnicity	Age in years at time of lab visit Mean (SD) (Range)	*T*-test for age at time of lab visit
Discovery						
Discovery cohort (within-subject changes in memory retention)	159 (with 496 visits)	Male = 131(414) Female = 28(82)	BP = 52 (187) MDD = 23(64) SZA = 35(97) SZ = 27(82) PTSD = 14 (43) MOOD = 5(14) PSYCH = 3 (9)	EA = 107(347) AA = 47(135) Asian = 1(2) Hispanic = 3(9) Biracial = 1(3)	50.26 (8.97) (22–66)	
Testing						
Independent testing cohort for predicting state (low memory retention ≤40 at time of assessment)	127 (238 visits)	Male = 97(176) Female = 30(62)	BP = 37 (73) MDD = 24(48) SZA = 27(48) SZ = 23(42) PTSD = 12(20) MOOD = 2(5) PSYCH = 2(2)	EA = 86(162) AA = 40(73) Asian = 1(3)	50.48 (8.2) (23–74) Low memory retention = 50.9 (10.9) Others = 50.32 (6.83)	Low memory retention (*n* = 68) vs. Others (*n* = 170) 0.703983
Independent testing cohort for predicting trait (future positive neuropsych testing for dementia in all years following assessment)	56 (111 visits)	Male = 47(91) Female = 9(20)	BP = 11(23) MDD = 13(26) SZA = 11(20) SZ = 15(30) PTSD = 5(10) MOOD = 1(2)	EA = 33(64) AA = 23(47)	55.6 (5.0) (40–74) Neuropsych testing positive = 54.2 (6.05) Others = 55.8 (4.89)	Future positive neuropsych testing (*n* = 11) vs. Others (*n* = 100) 0.411644

Cohorts used in our study. *BP* bipolar, *MDD* major depressive disorder, *SZA* schizoaffective disorder, *SZ* schizophrenia, *PTSD* posttraumatic stress disorder

Similar to our previous studies [7–9], the psychiatric subjects are part of a larger longitudinal cohort of adults that we are continuously collecting. Subjects were recruited from the patient population at the Indianapolis VA Medical Center. All subjects understood and signed informed consent forms detailing the research goals, procedure, caveats and safeguards, per IRB approved protocol. Subjects completed diagnostic assessments by an extensive structured clinical interview—Diagnostic Interview for Genetic Studies, and up to six testing visits, 3–6 months apart or whenever a new psychiatric hospitalization occurred. At each testing visit, they received a series of rating scales, including a Hopkins Verbal Learning Test (HVLT-R), and the blood was drawn. We collected whole blood (10 ml) in two RNA-stabilizing PAXgene tubes, labeled with an anonymized ID number, and stored at −80 °C in a locked freezer until the time of future processing. Whole-blood RNA was extracted for microarray gene expression studies from the PAXgene tubes, as detailed below.

For this study, our within-subject longitudinal discovery cohort, from which the biomarker data were derived, consisted of 159 subjects (131 males, 28 females) with multiple testing visits (a total of 496), who each had at least one 20% change in the Retention measure of HVLT (Fig. S1) from one consecutive testing visit to another.

Our independent test cohort for predicting state (low memory retention) consisted of 127 subjects (97 males, 30 females), demographically matched with the discovery cohort, with one or more testing visits in our lab (for a total of 238 visits). Low Memory Retention was defined as a score of ≤ 40 (Fig. 1, Table 1 and Table S1). Subjects were matched for age between low memory group and the rest group.

Our test cohort for predicting trait (future positive neuropsychological testing for cognitive impairment) is a subset of the independent test cohort, that consisted of 56 subjects (47 males, 9 females), demographically matched with the discovery cohort, with one or more testing visits in our lab (for a total of 111 visits). Positive neuropsychological testing was defined as a diagnosis of MCI, ADRD (Alzheimer Disorder Related Dementia), or other dementia upon neuropsychological testing done in a clinical setting, triggered by clinical concerns as part of regular clinical care (Fig. 1, Table 1, and Table S1). Subjects were matched for age at time of lab visit between the positive neuropsychological testing group and the rest group.

#### Medications

The subjects in the discovery cohort were all diagnosed with various psychiatric disorders (Table 1), and had various medical co-morbidities. Their medications were listed in their electronic medical records, and documented by us at the time of each testing visit. Medications can have a strong influence on gene expression. However, our discovery of differentially expressed genes was based on within-subject analyses, which factor out not only genetic background effects but also minimizes medication effects, as the subjects rarely had major medication changes between visits. Moreover, there was no consistent pattern of any particular type of medication, as our subjects were on a wide variety of different medications, psychiatric and nonpsychiatric. Furthermore, the independent validation/testing cohorts’ gene expression data was *Z*-scored by gender and diagnosis before being combined, to normalize for any such effects. Some subjects may be noncompliant with their treatment and may thus have changes in medications or drug of abuse not reflected in their medical records. That being said, our goal is to find biomarkers that track memory retention, regardless if the reason for it is endogenous biology or driven by substance abuse or medication noncompliance. In fact, one would expect some of these biomarkers to be direct or indirect targets of medications, as we show in this paper. Overall, the discovery, prioritization, and validation/ replication by testing in independent cohorts of the biomarkers, with our design, occurs despite the subjects having different genders, diagnoses, being on various different medications, and other lifestyle variables.

### Blood gene expression experiments

#### RNA extraction

Whole blood (2.5–5 ml) was collected into each PaxGene tube by routine venipuncture. PaxGene tubes contain proprietary reagents for the stabilization of RNA. RNA was extracted and processed as previously described [7–9].

#### Microarrays

Microarray work was carried out using previously described methodology [7–10].

The dataset is available at GEO (GSE127711).

### Biomarkers

#### Step 1: Discovery

We have used the subject’s score from the HVLT- DR Retention measure, assessed at the time of blood collection (Fig. 1). Using a 20% change threshold in Retention, we analyzed gene expression differences between visits, using a powerful within-subject design, then an across-subjects summation (Fig. 1).

We analyzed the data in two ways: an absent–present (AP) approach, and a differential expression (DE) approach, as in previous work by us on suicide biomarkers [7–9]. The AP approach may capture turning on and off of genes, and the DE approach may capture gradual changes in expression. We used a within-subject design, then an across-subjects summation score for probesets. Analyses were performed as previously described [8–10]. In brief, we imported all Affymetrix microarray data as CEL. files into Partek Genomic Suites 6.6 software package (Partek Incorporated, St Louis, MI, USA). Using only the perfect match values, we ran a robust multi-array analysis (RMA) by gender and diagnosis, background corrected with quantile normalization and a median polish probeset summarization of all chips, to obtain the normalized expression levels of all probesets for each chip. Then, to establish a list of differentially expressed probesets we conducted a within- subject analysis, using a fold change in expression of at least 1.2 between high- and low-memory visits within each subject. For each comparison, probesets that have a 1.2-fold change are then assigned either a 1 (increased in high memory) or a -1 (decreased in high memory; 0.5 or −0.5 if the change is between 1.1 and 1.2 fold; and 0 if the change is less than 1.1 fold. These values were then summed for each probeset across all the comparisons and subjects, yielding a range of raw scores. The raw scores were converted into internal scores for the next step, CFG prioritization: probesets above the 33.3% of raw scores received an internal score of 2 points, those above 50% 4 points, and those above 80% 6 points [8–10]. This was done in order to normalize and be able to easily combine internal (discovery) and external evidence (prioiritzation) scores, and to avoid over-fitting to the discovery cohort. We have developed in our lab R scripts to automate and conduct all these large dataset analyses in bulk, checked against human manual scoring [10].

Gene Symbol for the probesets were identified using NetAffyx (Affymetrix) for the Affymetrix HG-U133 Plus 2.0 Arrays which were used in this study, followed by GeneCards to confirm the primary gene symbol. In addition, for those probesets that were not assigned a gene symbol by NetAffyx, we used GeneAnnot or UCSC to obtain gene symbol for these uncharacterized probesets, followed by GeneCard. Genes were then scored using our manually curated CFG databases as described below (Fig. 1).

#### Step 2: Prioritization using Convergent Functional Genomics (CFG)

##### Databases

We have established in our laboratory (Laboratory of Neurophenomics, www.neurophenomics.info) manually curated databases of the human gene expression/protein expression studies (postmortem brain, peripheral tissue/fluids: CSF, blood and cell cultures), human genetic studies (association, copy number variations and linkage), and animal model gene expression and genetic studies, published to date on psychiatric disorders. Only findings deemed significant in the primary publication, by the study authors, using their particular experimental design and thresholds, are included in our databases. Our databases include only primary literature data and do not include review papers or other secondary data integration analyses to avoid redundancy and circularity. These large and constantly updated databases have been used in our CFG cross validation and prioritization platform (Fig. 1). For this study, data from 213 papers on AD were present in the databases at the time of the CFG analyses (August 2018) (human genetic studies-62, human brain tissue studies-49, human peripheral tissue/fluids- 83, nonhuman genetic studies-4, nonhuman brain tissue studies-13, nonhuman peripheral tissue/fluids- 2). Analyses were performed as previously described, using these databases [8, 9].

##### CFG scoring

For CFG analyses [15, 16], external cross-validating lines of evidence were weighted such that findings in human studies are prioritized over animal model studies, and brain studies are prioritized over peripheral fluid or genetic findings, by giving them twice as many points. Each line of evidence is capped in such a way that any positive findings within that line of evidence result in maximum points, regardless of how many different studies support that single line of evidence, to avoid potential popularity biases. Thus, genetic data had a maximum of two points, human brain expression data 4 points, human peripheral expression data 2 points, nonhuman genetic data 1 point, nonhuman brain expression data 2 points, and nonhuman peripheral expression data had 1 point, for a total of 12 points (Table S2). In addition to our external score, we also prioritized genes based upon the initial differential expression analyses used to identify them. Probesets identified by within-subject differential expression analyses can receive a maximum of 6 internal points. Thus, the maximum possible total CFG score for each gene is 18 points (6 points for the internal score and 12 points for the external score), with the external evidence weighted twice as much as the internal evidence to prevent a fit to cohort effect. It has not escaped our attention that other ways of scoring the lines of evidence may give slightly different results in terms of prioritization, if not in terms of the list of genes per se. Nevertheless, this simple scoring system provides a good separation of genes based on differential expression and on independent cross-validating evidence in the field.

### Choice of biomarkers to be carried forward

We carried forward into testing the candidate biomarkers after the prioritization step, using as threshold a CFG score ≥ 10 (*n* = 138 probesets, 112 genes). A CFG score of 10 or above reflects an empirical cutoff of over half of the maximum possible total CFG score of 18, and permits the inclusion of potentially novel genes (with a maximal internal score of 6) and with some solid external prior literature evidence for involvement in AD. Of these, the top candidate biomarkers had a CFG score ≥ 12 (*n* = 23 probesets, 18 genes). In Step 3, testing, we then predict in independent cohorts state (low memory retention), and trait (future positive neuropsychological testing for cognitive impairment).

### Diagnostics

In Step 3, testing, the test cohort for predicting low memory retention (state), and the test cohort for predicting Future Positive Neuropsychological Testing (trait), were completely independent from the discovery and validation cohorts; there was no subject overlap with them. They were assembled out of data that was RMA normalized by gender and diagnosis. The expression values of markers used for predictions were furthermore *Z* scored by gender and diagnosis in order to avoid potential artefacts due to different ranges of expression in different gender and diagnoses, and to be able to combine different markers into panels. Markers in panels were combined by simple summation of the increased risk markers minus the decreased risk markers. Predictions were performed using R-studio. For cross-sectional analyses, we used marker expression levels, z-scored by gender and diagnosis. For longitudinal analyses, we combined four measures: marker expression levels, slope (defined as ratio of levels at current testing visit vs. previous visit, divided by time between visits), maximum levels (at any of the current or past visits), and maximum slope (between any adjacent current or past visits). For decreased markers, we used the minimum rather than the maximum for level calculations. All four measures were each Z-scored by gender and diagnosis, then combined in an additive fashion into a single measure. The longitudinal analysis was carried out in a sub-cohort of the testing cohort consisting of subjects that had at least two test visits.

#### Predicting state—low memory

**Fig. 2:**
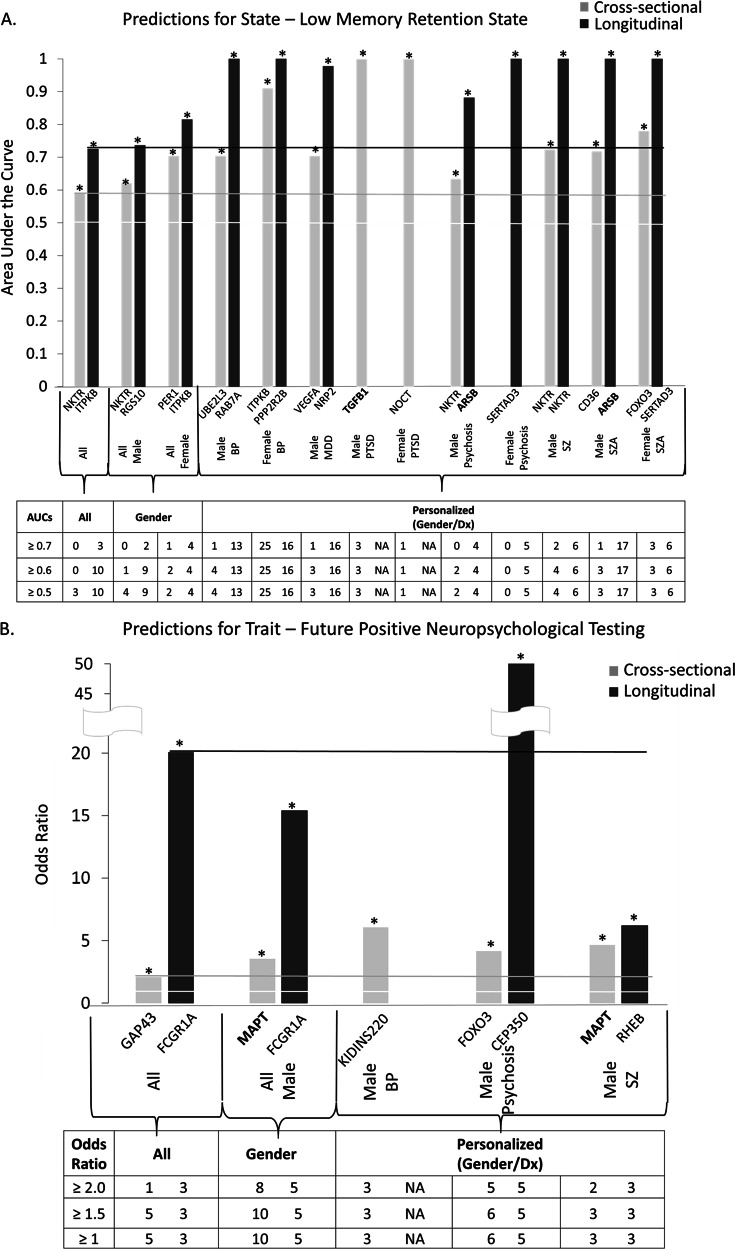
Best predictive biomarkers. **a** For state-low memory retention state. **b** For trait-future positive neuropsychological testing. From among the top candidate biomarker list (CFG score ≥ 10, *n* = 138 probesets). Bold- top CFG scoring biomarkers on the list (CFG ≥ 12, *n* = 23 probesets). Bar graph shows best predictive biomarkers in each group. * Nominally significant *p* < 0.05. Table underneath the figures displays the actual number of biomarkers for each group whose ROC AUC *p*-values (**a**) and Cox Regression Odds Ratio *p*-values (**b**) are at least nominally significant. Some female diagnostic group are missing from the graph as they did not have subjects to be tested or any significant biomarkers. Cross-sectional is based on levels at one visit. Longitudinal is based on levels at multiple visits (integrates levels at most recent visit, maximum levels, slope into most recent visit, and maximum slope). Dividing lines represent the cutoffs for a test performing at chance levels (white), and at the same level as the best biomarkers for all subjects in cross-sectional (gray) and longitudinal (black) based predictions. All biomarkers perform better than chance. Biomarkers performed better when personalized by gender and diagnosis

Receiver-operating characteristic (ROC) analyses between marker levels and memory state were performed by assigning subjects visits with a HVLT Retention score of ≤40 into the Low Memory category. We used the pROC package of R [17] (Fig. 2).

#### Predicting trait-future positive neuropsychological testing for cognitive impairment

We conducted analyses for predicting future positive neuropsychological testing performed as part of routine clinical care in subjects that had follow-up in the VA system, in which we have access to complete electronic medical records, using electronic medical records follow-up data of our study subjects (up to 12.81 years from initial visit at the time of the analyses). Analyses between marker measures (cross-sectional, longitudinal) at a specific testing visit and future positive neuropsychological test were performed a as described below, based on assigning if subjects had a future positive neuropsychological test for cognitive impairment or not. A Cox regression was performed using the time in days from the lab testing visit date to the positive neuropsychological testing date. The hazard ratio was calculated such that a value greater than 1 always indicates increased risk for positive neuropsychological testing, regardless if the biomarker is increased or decreased in expression.

### Biological understanding

### Clock gene database

We compiled a database of genes associated with circadian function, by using a combination of review papers and searches of existing databases CircaDB, GeneCards, and GenAtlas. Using the data we compiled from these sources we identified a total of 1468 genes that show circadian functioning. We further classified genes into “core” clock genes, i.e. those genes that are the main engine driving circadian function (*n* = 18), “immediate” clock genes, i.e., the genes that directly input or output to the core clock (*n* = 331), and “distant” clock genes, i.e., genes that directly input or output to the immediate clock genes (*n* = 1119).

### Pathway analyses

IPA (Ingenuity Pathway Analysis, Qiagen), David Functional Annotation Bioinformatics Microarray Analysis (National Institute of Allergy and Infectious Diseases), and Kyoto Encyclopedia of Genes and Genomes (KEGG) (through DAVID) were used to analyze the biological roles, including top canonical pathways and diseases (Table 2), of the candidate genes resulting from our work. We conducted analyses for the 112 unique genes (from 138 probesets) that came out of the prioritization step, and for the top 18 unique genes (from 23 probesets). For Network analysis of the 112 unique genes we performed STRING Interaction Network by inputting the genes into the search window and performed Multiple Proteins Homo sapiens analysis.

**Table 2:** Biological pathway analyses. For top candidate biomarkers after discovery and prioritization. **A**. Pathways for top biomarkers with CFG score ≥ 12. **B**. Pathways for biomarkers with CFG score ≥ 10. **C**. Diseases for top biomarkers with CFG score ≥ 12. **D**. Diseases for biomarkers with CFG score ≥10

	DAVID GO functional annotation biological processes					KEGG pathways				Ingenuity pathways		
	No.	Term	Count	%	*P*-value	Term	Count	%	*P*-value	Top canonical pathways	*P*-value	Overlap
**A. Pathways CFG ≥ 12**												
Top biomarkers (*n* = 18 genes, 23 probeset)	1	Positive regulation of peptidyl-tyrosine phosphorylation	3	16.7	3.10E−03	Alzheimer’s disease	5	27.8	3.80E−04	Neuroinflammation Signaling Pathway	3.04E−09	2.2 % 7/313
	2	Response to oxidative stress	3	16.7	5.40E−03	Lysosome	4	22.2	2.10E−03	Amyloid Processing	7.61E−08	7.8 % 4/51
	3	Neuron projection regeneration	2	11.1	8.10E−03	Glycosaminoglycan degradation	2	11.1	4.10E−02	Reelin Signaling in Neurons	5.86E−05	3.2 % 3/94
	4	Positive regulation of cardiac muscle cell differentiation	2	11.1	1.00E−02	Pathways in cancer	4	22.2	5.00E−02	14-3-3-mediated Signaling	1.87E−04	2.2 % 3/139
	5	Negative regulation of blood vessel endothelial cell migration	2	11.1	1.40E−02					Regulation of the Epithelial-Mesenchymal Transition Pathway	5.20E−04	1.5 % 3/197

**B. Pathways CFG** **≥** **10**												
Top biomarkers (*n* = 112 genes,138 probesets)	1	Cholesterol transport	4	3.6	1.40E−04	Alzheimer’s disease	11	9.8	1.10E−05	LXR/RXR activation	1.15E−08	7.4 % 9/121
	2	Positive regulation of peptidyl-tyrosine phosphorylation	6	5.4	1.90E−04	Nonalcoholic fatty liver disease (NAFLD)	8	7.1	1.10E−03	Neuroinflammation signaling pathway	6.82E−08	3.8 % 12/313
	3	Cellular response to hypoxia	6	5.4	4.00E−04	Osteoclast differentiation	7	6.2	2.70E−03	Atherosclerosis Signaling	4.14E−06	5.5 % 7/128
	4	Cholesterol efflux	4	3.6	5.50E−04	AMPK signaling pathway	6	5.4	1.00E−02	Role of Macrophages, Fibroblasts and Endothelial Cells in Rheumatoid Arthritis	6.03E−06	3.1 % 10/323
	5	Cholesterol import	3	2.7	6.20E−04	PI3K-Akt signaling pathway	10	8.9	1.10E−02	Amyloid Processing	6.05E−06	9.8 % 5/51

	DAVID					Ingenuity pathways disease		
	No.	Term	Count	%	*P*-value	Diseases and disorders	*P*-value	Molecules
**C. Diseases CFG** **≥** **12**								
Top biomarkers (*n* = 18 genes, 23 probeset	1	Alzheimer’s disease	12	66.7	2.50E−09	Neurological disease	2.26E−05 to 1.40E−15	16
	2	Depression	6	33.3	1.30E−06	Organismal injury and abnormalities	2.26E−05 to 3.26E−13	17
	3	Frontotemporal dementia	3	16.7	9.70E−06	Metabolic disease	2.12E−05 to 1.30E−10	15
	4	Lung cancer	7	38.9	3.30E−05	Psychological disorders	1.76E−05 to 1.77E−10	11
	5	Breast cancer	7	38.9	7.50E−05	Inflammatory response	2.05E−05 to 4.48E−10	15

**D. Diseases CFG** **≥** **10**								
Top biomarkers (*n* = 112 genes, 138 probesets)	1	Alzheimer’s disease	11	26	2.10E−18	Neurological disease	1.27E−06 to 2.30E−21	76
	2	atherosclerosis	8	17	1.00E−08	Organismal injury and abnormalities	1.32E−06 to 2.30E−21	204
	3	cognitive trait	7	10	2.70E−07	Psychological disorders	1.27E−06 to 2.30E−21	51
	4	Type 2 diabetes| edema | rosiglitazone	6	39	2.80E−07	Metabolic disease	1.27E−06 to 3.83E−21	49
	5	Aging/telomere length	1	10	2.90E−07	Skeletal and muscular disorders	1.32E−06 to 2.17E−17	64

### CFG beyond Alzheimer’s: evidence for involvement in other psychiatric and related disorders

We also used a CFG approach to examine evidence from other psychiatric and related disorders, for the list of top biomarkers after Step 3 testing (*n* = 36 genes, from 42 probesets) (Table S3).

### Therapeutic

#### Pharmacogenomics

We analyzed which of our top biomarkers from Table 3 (*n* = 36 genes, from 42 probesets) are known to be modulated by existing drugs using our CFG databases, and using Ingenuity Drugs analyses (Table 3 and Table S4).

**Table 3:** Top biomarkers. Convergent functional evidence for relevance to short-term memory tracking and Alzheimer disease (AD)

Genesymbol/Gene name	Probeset	Step 1 Discovery in blood (Direction of change tracking increased memory) method/ score/ % up to 6pts	Step 2 External CFG evidence for involvement in AD score up to 12 pt	Step 3 Best significant prediction of state Low memory retention ROC AUC/ *p*-value up to 6 pts ALL 4pts gender 2pts gender /Dx	Step 3 Best significant predictions of trait future positive neuropsych OR/OR *p*-value Up to 6 pts ALL 4pts gender 2pts gender /Dx	Other psychiatric and related disorders evidence(change in opposite direction to increased me mory)	Pharmacogenomics Drugs that modulate the biomarker (Change in Same Direction to Increased Memory)	CFE polyevidence score
RAB7A RAB7A, member RAS oncogene family	227602_at	(I) AP/2 43.8% (I) DE/4 69.6%	7	**ALL** **L:** (17/111) 0.66/1.7 3E−02 **Gender Dx** F-BP **L:** (2/9) 1/2.02 E−02 M-BP **L:** (1/27) 1/4.76E−02 M-PSYCHOSIS **L:** (8/27) 0.76/1.68E−02 M-SZ **L:** (5/14) 0.8/3.59E−02 M-SZA **C:** (12/33) 0.67/4.98E−02	**Gender** Male **C:** (7/91) 2.51/3.08E−02	BP Brain arousal depression MDD neuropathic pain	TCA Valproate	21
**NPC2** **Niemann-Pick disease, type C2**	200701_at	(D) DE/6 80.8%	8	**ALL** **L:** (17/111) 0.65/2.38E−02 **Gender** Male **L:** (12/79) 0.65/4.65E−02 **Gender Dx** M-MDD **L:** (3/18) 0.96/7.58E−03 M-SZA **L:** (3/13) 0.9/2.13E−02		Aging alcohol SZ		20
**TGFB1** **transforming growth factor beta 1**	203084_at	(I) AP/4 54.5%	9	**ALL** **C:** (68/238) 0.58/2.88E−02 **Gender** Male **C:** (53/176) 0.6/2.29E−02 **Gender Dx** M-PTSD **C:** (4/10) 1/5.26E−03 M-SZ **C:** (15/34) 0.68/3.99E−02		Aging ASD BP Chronic stress Depression Longevity Pain Phencyclidine PTSD Suicide SZ	Omega-3 fatty acids	19
GAP43 growth associated protein 43	204471_at	(I) DE/4 50.8%	7	**Gender Dx** M-SZA **L:** (3/13) 0.867/3.15E−02	**ALL** **C:** (11/111) 2.07/2.08E−02 **L:** (3/50) 6.14/1.51-02 **Gender** Male **C:** (7/91) 2.94/1.17E−02 **L:** (3/43) 5.54/1.47-02 **Gender-Dx** M-Psychosis **L:** (2/22) 5.4/2.96-02 M-SZ **L:** (2/13) 4.08/3.83-02	BP depression SZ stress	Valproate Benzodiazepines	19
**ARSB** **arylsulfatase B**	1554030_at	(I) DE/6 91.7%	6	**ALL** **L:** (17/111) 0.72/2.19E−03 **Gender** Male **L:** (12/79) 0.74/4.92E−03 **Gender Dx** F-BP **L:** (2/9) 0.93/3.95E−02 M-PSYCHOSIS **L:** (8/27) 0.88/1.04E−03 M-SZ **L:** (5/14) 0.8/3.59E−02 M-SZA **L:** (3/13) 1/5.61E−03		Alcohol Depression MDD Suicide		18
PER1 period circadian clock 1	242832_at	(I) DE/4 61.3%	6	**Gender** Female **C:**(15/62) 0.7/9.17E−03 **Gender Dx** F-BP **C:** (6/19) 0.83/1.13E−02 M-BP **L:** (1/27) 1/4.76E−02	**Gender** Male **L:**(3/43) 5.2/4.97E−03	Alcohol Anxiety ASD Autism BP Circadian abnormalities Depression MDD PTSD Sleep Duration Suicide SZ	Lithium Clozapine Quetiapine Avibactam	18
**GUSB** **glucuronidase, beta**	202605_at	(D) DE/4 55.7%	8	**ALL** **L:** (17/111) 0.65/2.16E−02 **Gender** Female **L:** (5/32) 0.79/2.29E−02 **Gender Dx** F-BP **C:** (6/19) 0.81/1.76E−02 M-MDD **L:** (3/18) 0.89/1.91E−02		Aging Methamphetamine	Clozapine	18
**MAPT** **microtubule associated protein tau**	203930_s_at	(I) DE/2 33.7%	10		**ALL** **L:** (11/111) 1.96/2.95E−02 **Gender** Male **C:** (7/91) 3.54/4.62E−02 **Gender Dx** M-PSYCHOSIS **C:** (5/47) 2.84/3.34E−02 M-SZ **C:** (4/27) 4.65/4.06E−02	Aging Alcohol Intellect MDD Methamphetamine Phencyclidine Stress Suicide SZ	Lithium Omega-3 fatty acids	18
FCGR1A Fc fragment of IgG, high affinity Ia, receptor (CD64)	216951_at	(I) DE/4 64.6%	7		**ALL** **L:** (3/49) 20/3.50-02 **Gender** Male **L:** (3/40) 15.4/4.37E−02			17
UBE2L3 ubiquitin conjugating enzyme E2L 3	200682_s_at	(D) DE/6 91%	4	**ALL** **L:** (17/111) 0.63/4.13E−02 **Gender** Male **L:** (12/79) 0.65/4.92E−02 **Gender Dx** M-BP **C:** (10/54) 0.7/2.25E−02 M-SZA **L:** (3/13) 0.9/2.13E−02		Aging Alcohol ASD Depression Stress SZ	Clozapine	16
NKTR natural killer cell triggering receptor	1570342_at	(D) AP/6 85%	4	**ALL** **C:** (68/238) 0.59/1.40E−02 **Gender** Male **C:** (53/176) 0.62/5.55E−03 **Gender Dx** M-BP **C:** (10/54) 0.68/3.56E−02 M-PSYCHOSIS **C:** (27/67) 0.63/3.19E−02 M-PSYCHOSIS **L:** (8/27) 0.72/3.55E−02 M-SZ **C:** (15/34) 0.72/1.38E−02 M-SZ **L:** (5/14) 1/1.35E−03		Alcohol BP Depression MDD Social Isolation Stress Suicide SZ		16
RHEB Ras homolog enriched in brain	243008_at	(D) AP/6 84.4% (D) DE/4 64.1%	4		**ALL** **C:** (11/111) 1.51/3.05E−02 **Gender** Male **C:** (7/91) 1.63/2.46E−02 **Gender Dx** M-PSYCHOSIS **C:** (5/47) 2.12/5.45E−03 **L:** (2/22) 9.69/1.68E−02 M-SZ **C:** (4/27) 1.82/1.78E−02 **L:** (2/13) 6.22/3.32E−02	Suicide Pain SZ	Antidepressants	16
**PTGS2** **prostaglandin-endoperoxide synthase 2 (prostaglandin G/H synthase and cyclooxygenase)**	1554997_a_at	(D) DE/4 76%	10	**Gender Dx** M-PTSD **C:** (4/10) 0.88/2.75E−02		Aggression Alcohol ASD BP Chronic Fatigue Syndrome Depression Depression-Related MDD Neurological Pain Phencyclidine Social Isolation Stress Stress Substances/Addictions Suicide	Antipsychotics Lithium Vorinostat	16
RGS10 regulator of G-protein signaling 10	214000_s_at	(I) DE/4 63.5%	6	**ALL** **L:** (17/111) 0.7/3.89E−03 **Gender** Male **L:** (12/79) 0.74/4.73E−03 **Gender Dx** F-BP **L:** (2/9) 0.93/3.95E−02 M-BP **L:** (1/27) 1/4.76E−02 M-MDD **L:** (3/18) 0.87/2.53E−02 M-SZ **C:** (15/34) 0.68/3.70E−02		Aging BP Female specific interpersonal-traumas Methamphetamine Post-Deployment PTSD PTSD Stress Suicide SZ		16
**MAPT** **microtubule associated protein tau**	203928_x_at	(I) DE/4 57.5%	10	**Gender Dx** F-BP **C:** (6/19) 0.81/1.76E−02		Aging Alcohol Intellect MDD Methamphetamine Phencyclidine Stress Suicide SZ	Lithium Omega-3 fatty acids	16
ITPKB inositol-trisphosphate 3-kinase B	232526_at	(I) DE/4 51.9%	6	**ALL** **L:** (17/111) 0.73/1.60E−03 **Gender** Male **L:** (12/79) 0.7/1.44E−02 Female **L:**(5/32) 0.79/2.29E−02 **Gender Dx** M-BP **L:** (1/27) 1/4.76E−02		Aging Alcohol MDD Phencyclidine Stress Suicide,SZ SZ		16
KIDINS220 kinase D-interacting substrate 220 kDa	214932_at	(I) DE/4 51.9%	6	**Gender Dx** F-BP **L:** (2/9) 0.93/3.95E−02	**Gender** Male **C:** (7/91) 2.49/3.78E−02 **Gender-Dx** M-BP **C:** (2/16) 6.06/4.18-02	Alcohol MDD Psychosis Pain Suicide Stress	Clozapine	16
**GSK3B** **glycogen synthase kinase 3 beta**	209945_s_at	(D) DE/4 50.3%	10	**Gender Dx** M-SZA **L:** (3/13) 0.93/1.40E−02		Aging Alcohol ASD BP BP,SZ MDD Stress Suicide SZ	Astaxanthin-DHA Antipsychotics Lithium Omega-3 fatty acids Ketamine lipoteichoic acid Valproate enzastaurin, glycogen synthase kinase-3beta inhibitor	16
SERTAD3 SERTA domain containing 3	219382_at	(D) DE/6 81.4%	5	**Gender** Female **L:** (5/32) 0.79/2.29E−02 **Gender Dx** F-BP **C:** (6/19) 0.81/1.76E−02 F-PSYCHOSIS **L:** (2/13) 1/1.50E−02 F-SZA **L:** (2/8) 1/2.28E−02		Alcohol ASD Aging		15
**APOE** **apolipoprotein E**	212884_x_at	(D) AP/2 34.1%	11	**Gender Dx** M-PTSD **C:** (4/10) 0.88/2.75E−02 **Gender Dx** M-SZ **L:** (5/14) 0.89/9.82E−03		Aggression Aging Alcohol Anxiet ASD BP Brain arousal MDD PTSD Stress Suicide SZ TBI	Omega-3 fatty acids	15
**UBE2I** **ubiquitin conjugating enzyme E2I**	233360_at	(D) DE/6 86.8%	6	**Gender Dx** F-PSYCHOSIS **L:** (2/13) 0.91/3.78E−02 F-SZA **L:** (2/8) 0.92/4.78E−02		Aging Alcohol ASD Hallucinations Mood State Stress	Clozapine	14
FOXO3 forkhead box O3	231548_at	(I) AP/2 38.9% (I) DE/6 82.3%	4	**Gender Dx** F-SZA **C:** (5/15) 0.78/4.32E−02	**Gender Dx** M-PSYCHOSIS **C:** (5/47) 4.14/4.58E−02	BP Cocaine Longevity PTSD Stress Suicide	Clozapine	14
**THRA** **thyroid hormone receptor, alpha**	214883_at	(I) DE/4 61.3%	8	**Gender Dx** F-BP **C:** (6/19) 0.79/2.18E−02 M-BP **L:** (1/27) 1/4.76E−02		Alcohol PTSD Stress Suicide SZ	3,5-diiodothyropropionic acid,denosumab/levothyroxine,amiodarone,levothyroxine,dextrothyroxine,L-triiodothyronine	14
ITPKB inositol-trisphosphate 3-kinase B	1554306_at	(D) AP/4 61.1% (D) DE/4 55.7%	6	**Gender** Female **L:**(5/32) 0.81/1.37E−02 **Gender Dx** F-BP **C:** (6/19) 0.91/2.50E−03 F-BP **L:** (2/9) 1/2.02E−02		Acute Stress Aging Alcohol ASD BP MDD Neurological Suicide SZ	Omega-3 fatty acids	14
**IGF1** **insulin-like growth factor 1 (somatomedin C)**	209542_x_at	(I) DE/4 54.1%	8	**Gender Dx** F-BP **C:** (6/19) 0.79/2.18E−02		Aggression Aging Alcoho Anxiety BP Depression Longevity PTSD SZ	Lithium Clozapine Fluoxetine (SSRI), Venlafaxine (SNRI) MEDI-573,BI 836845	14
**NPTX2** **neuronal pentraxin II**	213479_at	(I) DE/4 52.5%	8	**Gender Dx** F-BP **L:** (2/9) 0.93/3.95E−02		Alcohol Brain arousal Cocaine Depression MDD MDD,SZ Mood Disorders NOS Stress Suicide	Clozapine Fluoxetine	14
**GSTM3** **glutathione S-transferase mu 3 (brain)**	235867_at	(D) DE/4 52.1%	8	**Gender Dx** F-SZA **C:** (5/15) 0.78/4.32E−02		BP MDD SZ		14
**BACE1** **Beta-Secretase 1**	222463_s_at	(I) DE/2 44.8%	8		**Gender** Male **C:** (7/91) 1.97/3.78E−02	MDD Stress Suicide		14
**PSEN1** **presenilin 1**	203460_s_at	(D) DE/4 54.5%	9			Aging Alcohol Autism Depression Emotional Stability Neuroticism Suicide SZ	Omega-3 fatty acids	13
GFAP glial fibrillary acidic protein	203540_at	(I) DE/2 34.3%	9	**Gender Dx** F-BP **C:** (6/19) 0.77/3.28E−02		Addictions Alcohol BP MDD Stress Suicide SZ Yohimbine	Omega-3 fatty acids Clozapine	13
**TREM2** **triggering receptor expressed on myeloid cells 2**	219725_at	(I) DE/2 37.6%	11			BP SZ		13
NOCT nocturnin	220671_at	(D) AP/4 69.5%	6	**Gender Dx** F-PTSD **C:** (3/9) 1/1.01E−02		PTSD Post-Deployment PTSD		12
CEP350 centrosomal protein 350 kDa	204373_s_at	(D) DE/4 67.1%	6		**Gender Dx** M-PSYCHOSIS **L:** (2/22) 54.6/3.77E−02	Autism BP Cocaine Depression PTSD Stress Suicide SZ	Antidepressants, Fluoxetine	12
PPP2R2B protein phosphatase 2, regulatory subunit B, beta	205643_s_at	(I) DE/4 63.5%	6	**Gender Dx** F-BP **L:** (2/9) 1/2.02E−02		ADHD Aging Alcohol ASD Circadian abnormalities Longevity PTSD Suicide SZ		12
NRP2 neuropilin 2	222877_at	(I) DE/4 61.3%	6	**Gender Dx** M-MDD L:(3/18) 0.98/5.43E−03		Longevity MDD Phencyclidine Stress	Clozapine	12
**CTSS** **cathepsin S**	232617_at	(D) DE/4 56.9%	8			Aging Alcohol ASD BP Brain arousal Pain Suicide	Omega-3 fatty acids	12
VEGFA vascular endothelial growth factor A	211527_x_at	(I) DE/2 45.3%	8	**Gender Dx** M-MDD **C:** (11/38) 0.7/2.57E−02		Alcohol Anxiety BP Chronic Stress Depression Hallucinations Intellect MDD Pain MSK Stress Suicide SZ	Antipsychotics Fluoxetine Steroids	12
**MAPT** **microtubule associated protein tau**	233117_at	(I) DE/2 44.2%	10			Aging Alcohol Intellect MDD Methamphetamine Phencyclidine Stress Suicide SZ	Lithium Omega-3 fatty acids	12
**GSK3B** **glycogen synthase kinase 3 beta**	240562_at	(I) DE/2 39.2%	10			Aging Alcohol ASD BP MDD Methamphetamine Psychological Stress Stress Suicide SZ Yohimbine	Antipsychotics Antipsychotics Pregnenolone sulfate Fluoxetine (SSRI) Lithium mood stabilizing drugs Valproate	12
**GSK3B** **glycogen synthase kinase 3 beta**	242336_at	(D) AP/2 34.1%	10			Aging Alcohol ASD BP BP,SZ MDD Stress Suicide SZ	Astaxanthin-DHA Antipsychotics Lithium Omega-3 fatty acids Ketamine lipoteichoic acid Valproate enzastaurin, glycogen synthase kinase-3beta inhibitor	12
**BACE1** **Beta-Secretase 1**	224335_s_at	(I) DE/2 43.1%	8			MDD Stress Suicide		10

Bold—top biomarkers after discovery and prioritization (*n* = 23, CFG ≥ 12)). Underlined—best predictor in a category after testing of the longer list candidate biomarkers after discovery and prioritization (*n*  =  138, CFG ≥ 10), as depicted in Fig. 2. We tabulated into a convergent functional evidence (CFE) score all the evidence from discovery (up to six points), prioritization (up to 12 points), testing (State Memory Retention State and Trait Future Positive Neuropsychological Testing (up to six points each if significantly predicts in all subjects, four points if predicts by gender, two points if predicts in gender/diagnosis subgroups). The goal is to highlight, based on the totality of our data and of the evidence in the field to date, biomarkers that have all around evidence: track memory, are implicated in AD, and predict memory state and future dementia. Such biomarkers merit priority evaluation in future clinical trials. Red—increased in expression (I) in high memory states, blue—decreased in expression (D). DE—differential expression, AP—absent/present. C—cross-sectional analyses; L—longitudinal analyses, using levels and slopes from multiple visits. In all, by gender, and personalized by gender and diagnosis (gender/Dx), DE—differential expression, AP—absent/present. For Step 3 predictions, C-cross-sectional (using levels from one visit), L-longitudinal, M-males, F-females. MDD-depression, BP-bipolar, SZ-schizophrenia, SZA-schizoaffective, PSYCHOSIS- schizophrenia and schizoaffective combined, PTSD-post-traumatic stress disorder. This is a summary table—the Supplementary Information contains 336 references related to the data summarized here

#### New drug discovery/repurposing

We also analyzed which drugs and natural compounds are a match for the gene expression profiles of panels of our top biomarkers, using the Connectivity Map (https://portals.broadinstitute.org, Broad Institute, MIT), and the NIH LINCS L1000 database (Table 4).

**Table 4:** Therapeutics. New drug discovery/repurposing. **A**, **B**. Connectivity Map [40, 41] (CMAP) analysis. Query for signature is done using exact Affymetrix probesets and direction of change. Drugs that have same gene expression profile effects to our high memory retention biomarkers signatures. A score of 1 indicates the perfect match, i.e. the best potential therapeutic for increasing memory retention. **C**, **D**. NIH LINCS analysis using the L1000CDS2 (LINCS L1000 Characteristic Direction Signature Search Engine) tool. Query for signature is done using gene symbols and direction of change. Shown are compounds mimicking direction of change in high memory. A higher score indicates a better match

Rank	CMAP name	Score	Description
**A. Top biomarkers CFG ≥ 12 (*n* = 23 probesets; 7 increased and 6 decreased were present in HG-U133A array used by CMAP).**			
1	Verteporfin	1	A benzoporphyrin derivative, is a medication used as a photosensitizer for photodynamic therapy to eliminate the abnormal blood vessels in the eye associated with conditions such as the wet form of macular degeneration.
2	**Pioglitazone**	0.987	A drug of the thiazolidinedione (TZD) class with hypoglycemic (antihyperglycemic, antidiabetic) action, used to treat diabetes
3	***Salsolidine***	0.972	A tetrahydroisoquinoline isolated from plants of the genus *Salsola*. Tetrahydroisoquinolines are steroselective competitive inhibitors of the enzyme monoamine oxidase. They are also a competitive inhibitors of catechol-O-methyltransferase.
4	Sulfadimidine	0.97	A sulfonamide antibacterial.
5	**SB-203580**	0.968	Specific inhibitor of p38MAPK
6	Ronidazole	0.966	An antiprotozoal agent used in veterinary medicine
7	Mesalazine	0.961	Anti-inflammatory salycilate derivative used to treat ulcerative colitis
8	Dioxybenzone	0.946	An organic compound used in sunscreen to block UVB and short-wave UVA rays. It is a derivative of benzophenone.
9	**Metamizole**	0.942	A nonsteroidal anti-inflammatory drug
10	8-Azaguanine	0.936	A purine analog with antineoplastic activity

**B. Top Biomarkers CFG ≥ 10 (*n* = 138 probesets; 45 increased and 38 decreased were present in HG-U133A array used by CMAP).**			
1	**Levonorgestrel**	1	Progesterone derivative used as contraceptive. Progesterone and its derivatives have some evidence for promoting brain cell growth, at least in adult rats, and some studies have shown that it can improve cognitive performance in the aging mouse.
2	Aminohippuric acid	0.955	Nontoxic diagnostic tool to measure effective renal plasma flow
3	Meglumine	0.933	Meglumine, also known as megluminum or methylglucamine, belongs to the class of organic compounds known as hexoses. Often used as an excipient in pharmaceuticals. Methylglucamine orotate is a memory-improving drug, altough the ortoate component was though to be the active compound.
4	mesalazine	0.932	Nonsteroidal antiinflamatory drug used to treat inflammatory bowel diseases
5	**Lymecycline**	0.92	Tetracycline antibiotic; tetracyclines have been shown to have beneficial effects in neurodegenerative diseases
6	Torasemide	0.918	diuretic
7	Dioxybenzone	0.916	Sunscreen compound
8	***Ginkgolide A***	0.915	A natural compound with neuroprotective and possible AD preventing effects
9	Rimexolone	0.907	Rimexolone is a derivative of prednisolone, a synthetic glucocorticoid with anti-inflammatory and immunosuppressive property.
10	Ketanserin	0.905	Ketanserin is a selective serotonin receptor antagonist with weak adrenergic receptor blocking properties. The drug is effective in lowering blood pressure in essential hypertension. It also inhibits platelet aggregation. It is well tolerated and is particularly effective in older patients.

Rank	Score	Drug	Description
**C. Top biomarkers CFG ≥ 12 (*n* = 23 probesets, 18 unique genes; 8 increased and 10 decreased).**			
1	0.2941	BRD-K03371390	7-fluoro-6-methoxy-2,3,4,9-tetrahydro-1H-pyrido[3,4-b]indol-1-one
2	0.2941	NCGC00185923-01	3-[[4-(2,6-Difluoro-4-methoxyphenyl)sulfonyl-1,4-diazepan-1-yl]sulfonyl]aniline
3	0.2353	BENZANTHRONE	dye that binds to amyloid fibrils
4	0.2353	SQ 22536	adenylyl cyclase inhibitor
5	0.2353	***ICARIIN***	prenylated flavanol glycoside from *Epimedium sagittatum*
6	0.2353	YM 90709	IL-5 receptor antagonist
7	0.2353	QUIPAZINE MALEATE	binds to serotonin receptors, particularly to 5HT2A and 5HT3
8	0.2353	Cisapride	serotonin 5-HT_4_ receptor agonist
9	0.2353	LEUCINE ENKEPHALIN	enkephalin
10	0.2353	BRD-K15318909	Prima-1, anticancer agent

**D. Top biomarkers CFG ≥ 10 (*n* = 112 unique genes; 68 increased and 64 decreased).**			
1	0.1048	Proparacaine hydrochloride	Local anesthetic
2	0.0952	BRD-K00944562	[(4S,5S)-5-(2-Azidophenyl)-4-[2-(benzenesulfonyl)ethyl]-2-[4-(3-hydroxypropoxy)phenyl]-5H-1,3-oxazol-4-yl]-piperidin-1-ylmethanone
3	0.0952	BRD-A80151636	(6Ar)-5-bromo-N-[(1S,4R)-2-hydroxy-7-(2-methylpropyl)-5,8-dioxo-4-propan-2-yl-3-oxa-6,9-diazatricyclo[7.3.0.02,6]dodecan-4-yl]-7-methyl-6,6a,8,9-tetrahydro-4H-indolo[4,3-fg]quinoline-9-carboxamide
4	0.0952	BRD-K05361803	5-Chloro-N-heptylnaphthalene-1-sulfonamide PKC activator
5	0.0952	BRD-K82137294	1-[(2S,3S)-2-[[Benzenesulfonyl(methyl)amino]methyl]-5-[(2R)-1-hydroxypropan-2-yl]-3-methyl-6-oxo-3,4-dihydro-2H-1,5-benzoxazocin-8-yl]-3-[4-(trifluoromethyl)phenyl]urea
6	0.0952	BRD-K34206396	N-[(2S,3S)-2-[[1,3-Benzodioxol-5-ylmethyl(methyl)amino]methyl]-5-[(2R)-1-hydroxypropan-2-yl]-3-methyl-6-oxo-3,4-dihydro-2H-1,5-benzoxazocin-8-yl]-2-(1-methylindol-3-yl)acetamide
7	0.0952	**Pioglitazone**	A drug of the thiazolidinedione (TZD) class with hypoglycemic (antihyperglycemic, antidiabetic) action, used to treat diabetes
8	0.0857	TENOXICAM	Nonsteroidal anti-inflammatory drug
9	0.0857	BRD-K64642496	Tert-butyl 3-[(2S,5S,8S)-14-methoxy-2-(2-methylpropyl)-4,7-dioxo-3,6,17-triazatetracyclo[8.7.0.03,8.011,16]heptadeca-1(10),11,13,15-tetraen-5-yl]propanoate Inhibitor of breast cancer resistance protein
10	0.0857	BRD-K18364651	N-[(3R,9S,10S)-9-[[Cyclohexanecarbonyl(methyl)amino]methyl]-12-[(2S)-1-hydroxypropan-2-yl]-3,10-dimethyl-13-oxo-2,8-dioxa-12-azabicyclo[12.4.0]octadeca-1(14),15,17-trien-16-yl]pyridine-4-carboxamide

Bold—drugs known to have pro-cognitive effects, which thus serve as a *de facto* positive control for our approach. Italic—natural compounds.

### Convergent functional evidence (CFE)

**Fig. 3:**
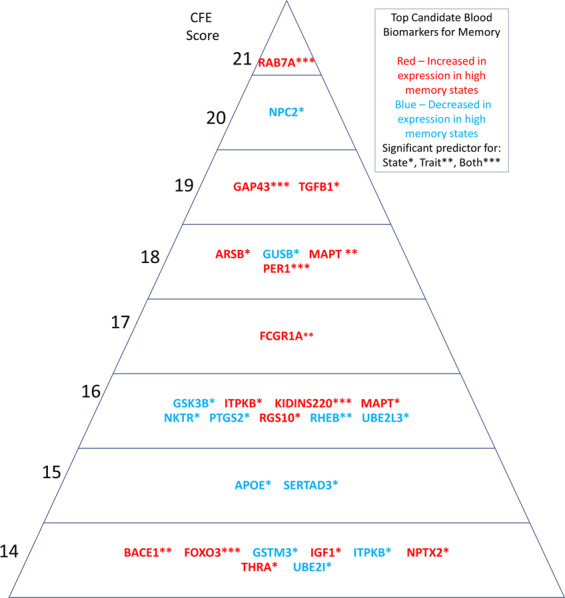
Convergent functional evidence for involvement in memory and AD. Genes from Table 3

For the top biomarkers (Table 3 and Fig. 3), we tabulated into a convergent functional evidence (CFE) score all the evidence from discovery (up to 6 points), prioritization (up to 12 points), testing (state, trait - up to 6 points each if significantly predicts in all subjects, 4 points if predicts by gender, 2 points if predicts in gender/diagnosis). The total score can be up to 30 points: 18 from our data and 12 from literature data. We weigh our data more than the literature data, as ours is direct functional evidence for the involvement of the gene/biomarker in the phenotype. The goal is to highlight, based on the totality of our data and of the evidence in the field to date, biomarkers that have all around evidence: track memory, are reflective of AD and related pathology, and predict it. Such biomarkers merit priority evaluation in future clinical trials.

## Results

In Step 1 Discovery, we identified blood gene expression biomarkers that track memory using the Retention measure from HVLT. At a phenotypic level, the Retention measure normalizes memory retention measurements in each subject during a testing visit, comparing the latter delayed recall trials with the earlier ones in that particular testing visit. We then used a powerful within –subject design in a cohort of subjects who displayed at least a 20% change in the Retention measure between different testing visits (*n* = 159 subjects, with 496 visits), to identify differentially expressed genes that track memory retention. Using our 33% of maximum raw score threshold (internal score of 2 pts, we had 10,941 unique probesets (Fig. 1). These were carried forward to the prioritization step. This represents approximately a 5-fold enrichment of the 54,625 probesets on the Affymetrix array.

In Step 2 Prioritization, we used a Convergent Functional Genomics (CFG) approach to prioritize the the candidate biomarkers identified in the discovery step (33% cutoff, internal score of ≥ 2 pt.) by using published literature evidence (genetic, gene expression and proteomic), from human and animal model studies, for involvement in AD (Fig. 1 and Table S2). There were 138 probesets that had a CFG score (combined internal and external score) of 10 and above. These were carried forward to the testing in independent cohorts step (see Supplementary Information- Complete Data and Analyses). This represents approximately a 400-fold enrichment of the probesets on the Affymetrix array.

In Step 3 Testing, we examined in an independent cohort (*n* = 127) from the one used for discovery whether the longer list of biomarkers prioritized in Step 2 (*n* = 138, CFG ≥ 10) are predictive of memory retention measure (state), and of future positive neuropsychological testing for MCI, AD or other dementia (trait), using electronic medical records follow-up data of our study subjects (up to 12.81 years from initial visit at the time of the analyses- December 2018) (Fig. 1, Table 1, and Table S1). The gene expression data in the test cohorts was normalized (Z-scored) across genders and various psychiatric diagnoses, before those different demographic groups were combined. We used biomarker levels information cross-sectionally, as well as expanded longitudinal information about biomarker levels at multiple visits, as predictors. We tested the biomarkers in all subjects in the independent test cohort, as well as in a more personalized fashion by gender and psychiatric diagnosis. We were successful in identifying gene expression biomarkers that were predictive in the independent cohorts for memory state, and for future neuropsychological testing positive for cognitive decline. We show increased accuracy with the personalized approach. In general, the longitudinal information was more predictive than the cross-sectional information.

Top predictive biomarkers for state were ITPKB, NKTR, RGS10, and PER1 (Fig. 2a and Table 3). The AUC ROCs ranged from over 0.6 for all subjects tested, to over 0.8 personalized by gender, and over 0.9 personalized by gender and diagnosis. NKTR is illustrative in this regard. It has a very modest AUC across all (0.59/*p* = 1.40E−02), higher in males (0.62/*p* = 5.55E−03), even more so in males with schizophrenia using cross-sectional levels of the biomarker (0.72/p = 1.38E-02), and the highest in male with schizophrenia using longitudinal levels of the biomarker (1/*p* = 1.35E−03). NKTR (Natural Killer Cell Triggering Receptor) is increased in expression in blood in low memory states in our study. There is some previous evidence showing it is increased in expression in the hippocampus in Alzheimer [18], but not as a top finding, and there is no previous functional evidence as a blood biomarker for memory or AD. Interestingly, it is a top biomarker for stress, increased in expression in high stress states stress states in a recent study from our group [12]. NKTR was also reported increased in expression in blood in studies of social isolation in humans [19], and in brain in studies of chronic variable stress in mice by Nestler and colleagues [20]. NKTR is also increased in expression in our previous blood biomarker studies of suicide, in both males [8, 21], and females [22], as well as increased in expression in postmortem brain studies in depression [23] and in schizophrenia [24], possibly underlying the effect of stress on those disorders and, based on our new data, on decreasing memory retention and promoting AD.

Top predictive biomarkers for trait were FCGR1A, GAP43, and MAPT (Fig. 2b and Table 3). Their Cox Regression Odds Ratios are at least 2-fold or higher, and significant. Caution should be used in interpreting these results because of the small Ns. We illustrate the virtues of personalization by gender and diagnosis with the example of RHEB in male schizophrenia, as our only future ADRD conversion to date was a male with schizophrenia (Figure S2A, B).

Based on our studies and analyses, the biomarkers with the best overall convergent functional evidence (CFE) for relevance to memory and AD were some new genes such as RAB7A, NPC2, TGFB1, GAP43, ARSB, PER1, GUSB, and MAPT (tau), as well as the well-known GSK3B, PTGS2, APOE, BACE1, PSEN1, and TREM2 (Table 3 and Fig. 3), with previous genetic and/or animal model evidence, that now have functional evidence in humans from our studies. The fact that key genes for AD brain pathology came out of our unbiased whole-genome discovery step tracking memory is reassuring, and they serve as de facto positive controls for our approach. Some of our new genes have previous supportive convergent evidence, buried in previous datasets; they were not considered strong candidate genes to be involved in AD before, and had no functional evidence as blood biomarkers for memory. An example is ARSB, a top biomarker from discovery, and our strongest predictor for low memory state in subjects with psychosis (SZ, SZA), with an AUC of 0.88/p-value 1.04E-03. ARSB (arylsulfatase B) is decreased in expression in blood low memory states in our studies. It has previous evidence for being decreased in expression in hippocampus and other brain regions in MCI [25], and genetic evidence for association with hippocampal volume in AD [26]. Additional new genes/biomarkers, with strong evidence for tracking memory state in the discovery step, are NKTR (natural killer cell triggering receptor), RHEB (Ras homolog enriched in brain), UBE2L3 (ubiquitin conjugating enzyme E2L 3), FOXO3 (forkhead box O3), SERTAD3 (SERTA domain containing 3), and UBE2I (ubiquitin conjugating enzyme E2I).

**Fig. 4:**
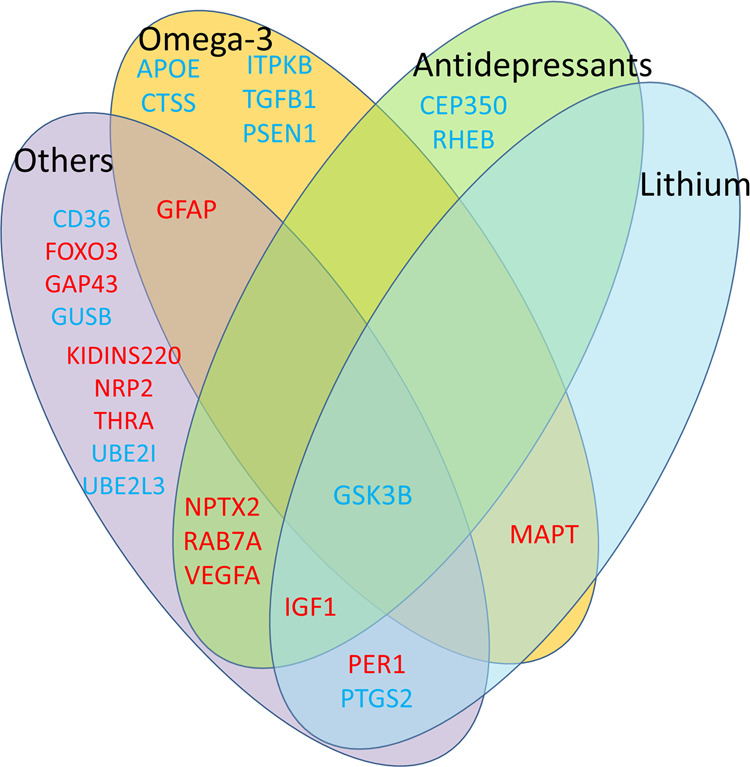
Pharmacogenomics: Top biomarkers (from Table 3) modulated by existing drugs

Some of the biomarkers are targets of existing drugs, such as lithium, antidepressants, and omega-3 fatty acids (Fig. 4, Table S4), of potential utility in patient stratification and pharmacogenomics approaches. Moreover, the top biomarkers gene expression signature, upon bioinformatics drug repurposing analyses (Table 4), yielded new drug candidates (such as pioglitazone and levonorgestrel), and natural compounds (such as salsolidine, ginkgolide A and icariin). The signature could be used for targeted enrollment of patients in clinical trials for these compounds, which would increase the odds of success, and for objectively measuring response to treatment.

## Discussion

We describe a novel approach for discovering blood biomarkers of relevance to AD, and testing them in independent cohorts. The biomarkers also open a window into understanding the biology of memory disorders in general, and of AD and related disorders in particular.

### Brain–blood

Blood biomarkers offer real-world clinical practice advantages. As the brain cannot be readily biopsied in live individuals, and CSF is less easily accessible than blood, we have endeavored over the years to identify blood biomarkers for neuropsychiatric disorders. A whole–blood approach facilities field deployment of sample collection. The assessment of gene expression changes focuses our approach on immune cells. The ability to identify peripheral gene expression changes that reflect brain activities is likely due to the fact that the brain and immune system have developmental commonalities, marked by shared reactivity and ensuing gene expression patterns. There is also a bi-directional interaction between the brain and immune system. Not all changes in expression in peripheral cells are reflective of or germane to brain activity. By carefully tracking a phenotype with our within subject design, and using convergent functional genomics prioritization, we are able to extract the peripheral changes that do track and are relevant to the brain activity studied, in this case memory and AD.

### Power considerations

Based on our work for the last two decades in genetics and gene expression, along with the results of others in the field, we estimate that the within-subject longitudinal design, by factoring out all genetic and some environmental variability, is up to 3 orders of magnitude more powerful than an inter-subject case-control cross-sectional design. Moreover, gene expression, by integrating the effects of many SNPs and environment, is up to 3 orders of magnitude more powerful than a genetic study. Combined, our approach may be up to 6 orders of magnitude more powerful than a GWAS study, even prior to the CFG literature-based prioritization step.

As an illustration of this, after the completion of our analyses, two major genetic studies came out. The first was a large scale genetic meta-analysis of clinically diagnosed AD and AD-by-proxy (71,880 cases, 383,378 controls) (Jansen et al. Nature Genetics 2019) [27]. 14 of their 29 genome-wide significant loci/ genes were present in the candidate gene expression biomarkers for memory list that had survived our initial whole-genome unbiased within-subject Discovery step, before any CFG literature prioritization (APOE, TREM2, ABCA7, APH1B, BIN1, MS4A6A, PICALM, SLC24A4, HLA-DRB1, CNTNAP2, CASS4, CD33, CD2AP, and ADAM10). Two more of the 29 had anti-sense transcripts in our candidate biomarker list from Discovery (EPHA1-AS1 and ABI3-AS1) (see Supplementary Information- Complete Data and Analyses). The second was a large scale genetic meta-analysis of clinically diagnosed late-onset Alzheimer’s Disease (94,437 individuals) [28]. 15 of their 24 genome-wide significant loci/ genes were present in the candidate biomarker for memory list that had survived our initial whole-genome unbiased within-subject Discovery step, before any CFG literature prioritization (TREM2, ABCA7,INPP5D, BIN1, MS4A6A, PICALM, FERMT2, SLC24A4, HLA-DRB1, CASS4, CD2AP, IQCK, WWOX, ACE and ADAM10). One more of the 24 had anti-sense transcripts in our candidate biomarker list from Discovery (EPHA1-AS1) (see Supplementary Information- Complete Data and Analyses). This independent reproducibility of findings between our studies and the genetic studies, which are done in independent cohorts from ours, with independent methodologies, is reassuring, and provides strong convergent evidence for the validity and relevance of our approach and of the GWAS approach. Our work also provides functional evidence for some of the GWAS top hits. For example, APOE, a strong finding from genetic studies of AD, seems to be involved in pathology by levels of expression also, not just mutant forms. In fact, there is prior evidence that APOE promoter region SNPs may be involved in the pathology of AD [29]. APOE may be involved in particular in memory impairment [30].

### Pathophysiological insights

A number of top biomarkers identified by us have biological roles that are related to the circadian clock (Table S3). To be able to ascertain all the genes in our dataset that were circadian and do estimates for enrichment, we compiled from the literature a database of all the known circadian genes, numbering a total of 1468 genes. Using an estimate of about 21 000 genes in the human genome, that gives about 7% of genes having some circadian pattern. Out of our 112 candidate biomarker genes, 19 had circadian evidence (16.96%), including the clock core gene PER1, suggesting a 2.4-fold enrichment for circadian genes. This provides a molecular underpinning for the epidemiological data between disrupted sleep and risk of AD [31, 32], and for the clinical phenomenology of “sundowning”.

The top candidate biomarkers also had prior evidence of involvement in other psychiatric and related disorders (Table S3), providing a molecular underpinning for the possible precursor effects of these disorders in AD. In particular, a majority of them have an overlap with stress (84%), aging (49%) and suicide (75%), consistent with them being part of the effects of stress on aging and the “life switch”, as described in a previous study by us [14]. The primary medications used to treat stress disorders are serotonin reuptake inhibitors (SSRIs). A recent study has shown that long-term SSRI treatment may delay progress from MCI to AD [33].

The top biological pathways where the candidate biomarkers from discovery and prioritization map were related to LXR/RXR activation, neuroinflammation signaling and atherosclerosis signaling (Table 2B). Agonists of nuclear receptors LXR:RXR and PPAR:RXR are known to ameliorate AD-related cognitive impairment and amyloid accumulation in murine models of AD. Interestingly, the ω-3 fatty acid docosahexaenoic acid (DHA), in combination with RXR agonist bexarotene, enhances LXR:RXR target gene expression of Abca1 and ApoE, reduces soluble forms of Aβ, and abrogates release of pro-inflammatory cytokines and mediators both in vitro and in a mouse model of AD [34]. Neuroinflammation is postulated to be a central mechanism in AD [35]. Atherosclerosis, and more general cardiovascular disorders, are recognized clinical risk factors for AD. The STRING gene interaction analysis (Fig. S3) revealed at least 3 networks. Network 1 (red) includes TREM2, along with ITPKB and FCGR1A; it may be involved in reactivity and inflammatory responses. Network 2 (green) includes GSK3B, along with MAPT (tau) and BACE1; it may be involved in activity and cellular trophicity. Network 3 (blue) includes PSEN1, along with FOXO3 and RHEB; it may be involved in connectivity and synaptic integrity. APOE is at the overlap of Networks 1 and 2. It is conceivable mechanistically that stress can lead to inflammation, followed by neuronal death (apoptosis) and synaptic connectivity loss, resulting in decreased memory and ultimately dementia.

The majority of top blood biomarkers we have identified have prior evidence in postmortem brain datasets from AD, which indicates their relevance to the pathophysiology of AD (Table S2). The co-directionality of blood changes in our work and brain changes reported in the literature needs to be interpreted with caution, as it may depend on brain region and on disease stage. Nevertheless, it is intriguing that three well known genes for AD, TREM2, MAPT (tau), and BACE1 are changed in the same direction (increased in expression) in blood in high memory states in our cohorts as they are in AD samples (Table S2). Consistent with the above discussion, RHEB is also changed in same direction (decreased) in high memory states in our data, and in AD brains in previous datasets, which increases BACE1 levels and amyloid β generation [36]. APP itself does not make the threshold as a candidate biomarker for memory in our discovery approach, but BACE 1 does, and it is increased in expression in high memory states. So is the APP-related gene APLP2 (data not shown). This opens the possibility that these genes are part of normal memory function, and their increase in AD is compensatory and/or excessive, like a scar formation. If so, treatments that target them may backfire in early stages of the disease, or need to be very carefully dosed in later stages of the disease. It is notable that various efforts to target amyloid accumulation have not been successful in multiple clinical trials to date.

The fact that GSK3B is a top candidate biomarker, has interesting neurobiological and therapeutic implications. GSK3B is decreased in expression in high memory states in our current work, and is mostly increased in expression in psychiatric disorders, including depression and stress related ones (Table S3), suggesting an avenue to their impact on memory and on later life AD. It is a known target of lithium [37], a medication that could be used for memory disorders improvement and prevention of AD, particularly in individuals with mood disorders earlier in life. Lithium is known to have anti-apoptotic roles [38, 39], so it is possible that it may prevent the neuronal loss of viability that is a precursor and part of AD, and which in turn triggers and is accentuated by the scar-like deposition of MAPT and APP.

### Pharmacogenomics and drug repurposing

Besides GSK3B, lithium also modulates expression of MAPT, PER1, PTGS2 and IGF1 (Fig. 4). GSK3B and MAPT are also modulated by omega-3 fatty acids. Such information could be useful for personalized treatment selection, and for monitoring response to treatment. Moreover, the drug repurposing analyses using the biomarker signatures from our studies (Table 4) yielded drug candidates such as pioglitazone (a diabetes medication, previously studied for AD prevention in clinical trials that may have been too heterogeneous in enrollment), levonorgestrel (a progesterone derivative), and the natural compounds salsolidine (a natural compound with acetylcholinesterase inhibitory properties), ginkgolide A (a natural compound with neuroprotective effects) and icariin (a natural compound reported to improve memory impairment in Alzheimer’s disease model mice). We now have human functional data-based evidence for the potentially utility of these drugs. Various abbreviated signatures present in individual patients could be used in the future to choose the best repurposed drug fit for each patient.

### Diagnostics

Our data for assessment and predictions needs to be interpreted with caution, particularly for the trait predictions, due to the small Ns. Nevertheless, the results are obtained in an independent cohort from the one used in discovery, and suggest interesting leads, as well as the fact that gender differences will be important for clinical applications. We would propose that a panel of our top genes, such as those in Table 3, be tested for predictions in other independent cohorts, and potentially be used in clinical settings. A comination of phenomic data (clinical information, imaging) and and biomarker data may work best. Of note, however, the HVLT was not a significant trait predictor in our cohort (data not shown).

## Conclusions

Our work has provided evidence for novel possible precision medicine approaches, diagnostic and therapeutic. In particular, it may lead to improved early objective assessment of state, of future risk, and to targeted preventive treatments (in essence, a risk evaluation and mitigation system) for memory disorders in general, and AD in particular, that result in decreased quality and quantity of life, at a massive cost to individuals, families and society. It also opens a novel window into disease pathophysiology, and may lead to new targets for drug development. Given the growing world-wide burden of AD, and the unsuccessful approaches to date, such new avenues should be pursued with vigor and alacrity.

## Supplementary information

Supplementary Information- Table S1 Detailed Demographics

Supplementary Information -Figures S1-S3 and Tables S2-S4

Supplementary Information-Complete Data and Analyses

## References

[1] Bilbo SD, Schwarz JM (2012). The immune system and developmental programming of brain and behavior. Front Neuroendocrinol.

[2] Le-Niculescu H, McFarland MJ, Ogden CA, Balaraman Y, Patel S, Tan J (2008). Phenomic, convergent functional genomic, and biomarker studies in a stress-reactive genetic animal model of bipolar disorder and co-morbid alcoholism. Am J Med Genet B Neuropsychiatr Genet.

[3] Le-Niculescu H, Kurian SM, Yehyawi N, Dike C, Patel SD, Edenberg HJ (2009). Identifying blood biomarkers for mood disorders using convergent functional genomics. Mol Psychiatry.

[4] Kurian SM, Le-Niculescu H, Patel SD, Bertram D, Davis J, Dike C (2011). Identification of blood biomarkers for psychosis using convergent functional genomics. Mol Psychiatry.

[5] Le-Niculescu H, Balaraman Y, Patel SD, Ayalew M, Gupta J, Kuczenski R (2011). Convergent functional genomics of anxiety disorders: translational identification of genes, biomarkers, pathways and mechanisms. Transl Psychiatry.

[6] Witt SH, Sommer WH, Hansson AC, Sticht C, Rietschel M, Witt CC (2013). Comparison of gene expression profiles in the blood, hippocampus and prefrontal cortex of rats. In Silico Pharmacol.

[7] Le-Niculescu H, Levey DF, Ayalew M, Palmer L, Gavrin LM, Jain N (2013). Discovery and validation of blood biomarkers for suicidality. Mol Psychiatry.

[8] Niculescu AB, Levey DF, Phalen PL, Le-Niculescu H, Dainton HD, Jain N (2015). Understanding and predicting suicidality using a combined genomic and clinical risk assessment approach. Mol Psychiatry.

[9] Levey DF, Niculescu EM, Le-Niculescu H, Dainton HL, Phalen PL, Ladd TB (2016). Towards understanding and predicting suicidality in women: biomarkers and clinical risk assessment. Mol Psychiatry.

[10] Niculescu AB, Le-Niculescu H, Levey DF, Phalen PL, Dainton HL, Roseberry K (2017). Precision medicine for suicidality: from universality to subtypes and personalization. Mol Psychiatry.

[11] Niculescu AB, Le-Niculescu H, Levey DF, Roseberry K, Soe KC, Rogers J (2019). Towards precision medicine for pain: diagnostic biomarkers and repurposed drugs. Mol Psychiatry.

[12] Le-Niculescu H, Roseberry K, Levey DF, Rogers J, Kosary K, Prabha S et al. Towards precision medicine for stress disorders: diagnostic biomarkers and targeted drugs. Mol Psychiatry. 2019. [Epub ahead of print]. 10.1038/s41380-019-0370-z PMC7192849 30862937

[13] Nho K, Ramanan VK, Horgusluoglu E, Kim S, Inlow MH, Risacher SL (2015). Comprehensive gene- and pathway-based analysis of depressive symptoms in older adults. J Alzheimer’s Dis.

[14] Rangaraju S, Levey DF, Nho K, Jain N, Andrews KD, Le-Niculescu H (2016). Mood, stress and longevity: convergence on ANK3. Mol Psychiatry.

[15] Bertsch B, Ogden CA, Sidhu K, Le-Niculescu H, Kuczenski R, Niculescu AB (2005). Convergent functional genomics: a Bayesian candidate gene identification approach for complex disorders. Methods.

[16] Le-Niculescu H, Patel SD, Niculescu AB (2010). Convergent integration of animal model and human studies of bipolar disorder (manic-depressive illness). Curr Opin Pharmacol.

[17] Robin X, Turck N, Hainard A, Tiberti N, Lisacek F, Sanchez JC (2011). pROC: an open-source package for R and S+ to analyze and compare ROC curves. BMC Bioinf.

[18] Blalock EM, Geddes JW, Chen KC, Porter NM, Markesbery WR, Landfield PW (2004). Incipient Alzheimer’s disease: microarray correlation analyses reveal major transcriptional and tumor suppressor responses. Proc Natl Acad Sci USA.

[19] Cole SW, Hawkley LC, Arevalo JM, Sung CY, Rose RM, Cacioppo JT (2007). Social regulation of gene expression in human leukocytes. Genome Biol.

[20] Labonte B, Engmann O, Purushothaman I, Menard C, Wang J, Tan C (2017). Sex-specific transcriptional signatures in human depression. Nat Med.

[21] Karege F, Perroud N, Burkhardt S, Fernandez R, Ballmann E, La Harpe R (2012). Protein levels of beta-catenin and activation state of glycogen synthase kinase-3beta in major depression. A study with postmortem prefrontal cortex. J Affect Disord.

[22] Canli T, Wen R, Wang X, Mikhailik A, Yu L, Fleischman D (2017). Differential transcriptome expression in human nucleus accumbens as a function of loneliness. Mol Psychiatry.

[23] Blasi G, Napolitano F, Ursini G, Di Giorgio A, Caforio G, Taurisano P (2013). Association of GSK-3beta genetic variation with GSK-3beta expression, prefrontal cortical thickness, prefrontal physiology, and schizophrenia. Am J Psychiatry.

[24] Glatt SJ, Everall IP, Kremen WS, Corbeil J, Sasik R, Khanlou N (2005). Comparative gene expression analysis of blood and brain provides concurrent validation of SELENBP1 up-regulation in schizophrenia. Proc Natl Acad Sci USA.

[25] Berchtold NC, Sabbagh MN, Beach TG, Kim RC, Cribbs DH, Cotman CW (2014). Brain gene expression patterns differentiate mild cognitive impairment from normal aged and Alzheimer’s disease. Neurobiol Aging.

[26] Potkin SG, Guffanti G, Lakatos A, Turner JA, Kruggel F, Fallon JH (2009). Hippocampal atrophy as a quantitative trait in a genome-wide association study identifying novel susceptibility genes for Alzheimer’s disease. PLoS One.

[27] Jansen IE, Savage JE, Watanabe K, Bryois J, Williams DM, Steinberg S et al. Genome-wide meta-analysis identifies new loci and functional pathways influencing Alzheimer’s disease risk. Nature Genet. 2019;51:404–13. 10.1038/s41588-018-0311-9 PMC6836675 30617256

[28] Kunkle BW, Grenier-Boley B, Sims R, Bis JC, Damotte V, Naj AC (2019). Genetic meta-analysis of diagnosed Alzheimer’s disease identifies new risk loci and implicates Abeta, tau, immunity and lipid processing. Nat Genet.

[29] Maloney B, Ge YW, Petersen RC, Hardy J, Rogers JT, Perez-Tur J (2010). Functional characterization of three single-nucleotide polymorphisms present in the human APOE promoter sequence: Differential effects in neuronal cells and on DNA-protein interactions. Am J Med Genet Part B, Neuropsychiatr Genet.

[30] Mukherjee S, Mez J, Trittschuh EH, Saykin AJ, Gibbons LE, Fardo DW et al. Genetic data and cognitively defined late-onset Alzheimer’s disease subgroups. Mol Psychiatry. 2018. [Epub ahead of print]. 10.1038/s41380-018-0298-8 PMC6548676 30514930

[31] Noble W, Spires-Jones TL (2019). Sleep well to slow Alzheimer’s progression?. Science.

[32] Irwin MR, Vitiello MV (2019). Implications of sleep disturbance and inflammation for Alzheimer’s disease dementia. Lancet Neurol.

[33] Bartels C, Wagner M, Wolfsgruber S, Ehrenreich H, Schneider A, Alzheimer’s Disease (2018). Impact of SSRI therapy on risk of conversion from mild cognitive impairment to Alzheimer’s dementia in individuals with previous depression. Am J Psychiatry.

[34] Casali BT, Corona AW, Mariani MM, Karlo JC, Ghosal K, Landreth GE (2015). Omega-3 fatty acids augment the actions of nuclear receptor agonists in a mouse model of Alzheimer’s disease. J Neurosci.

[35] Kinney JW, Bemiller SM, Murtishaw AS, Leisgang AM, Salazar AM, Lamb BT (2018). Inflammation as a central mechanism in Alzheimer’s disease. Alzheimers Dement.

[36] Shahani N, Pryor W, Swarnkar S, Kholodilov N, Thinakaran G, Burke RE (2014). Rheb GTPase regulates beta-secretase levels and amyloid beta generation. J Biol Chem.

[37] Mendes CT, Mury FB, de Sa Moreira E, Alberto FL, Forlenza OV, Dias-Neto E (2009). Lithium reduces Gsk3b mRNA levels: implications for Alzheimer disease. Eur Arch psychiatry Clin Neurosci.

[38] Fan M, Jin W, Zhao H, Xiao Y, Jia Y, Yin Y (2015). Lithium chloride administration prevents spatial learning and memory impairment in repeated cerebral ischemia-reperfusion mice by depressing apoptosis and increasing BDNF expression in hippocampus. Behavioural Brain Res.

[39] Liechti FD, Studle N, Theurillat R, Grandgirard D, Thormann W, Leib SL (2014). The mood-stabilizer lithium prevents hippocampal apoptosis and improves spatial memory in experimental meningitis. PLoS One.

[40] Lamb J, Crawford ED, Peck D, Modell JW, Blat IC, Wrobel MJ (2006). The Connectivity Map: using gene-expression signatures to connect small molecules, genes, and disease. Science.

[41] Lamb J (2007). The Connectivity Map: a new tool for biomedical research. Nat Rev Cancer.

