# Supplementary material for: Blood biomarkers for memory: toward early detection of risk for Alzheimer disease, pharmacogenomics, and repurposed drugs
Source: Mol Psychiatry. 2019 Dec 2;25(8):1651–72. doi: 10.1038/s41380-019-0602-2 (PMC7387316; doi:10.1038/s41380-019-0602-2)
Supplement: Supplementary file 1 — Supplementary Information- Table S1 Detailed Demographics [file 41380_2019_602_MOESM1_ESM.docx]

| **Table S1 Detailed Demographics** | | | | | | | | |
| --- | --- | --- | --- | --- | --- | --- | --- | --- |
| **A. Discovery Cohort (n=159) (496 visits)** | | | | | | | | |
| Participant ID visit number | Diagnosis | Gender (M/F) | Age at testing (Years) | Ethnicity | HVLT3 Memory Retention | **Discovery Cohort** | Test Cohort Memory Retention State (HVLT3) | Test Cohort Future Neuropsych Postitive Testing |
| phchp003v2 | SZ | M | 50 | African American | 20 | Yes | No | No |
| phchp003v3 | SZ | M | 50 | African American | 46 | Yes | No | No |
| phchp003v5 | SZ | M | 59 | African American | 43 | Yes | No | No |
| phchp004v2 | SZA | M | 60 | African American | 32 | Yes | No | No |
| phchp004v3 | SZA | M | 60 | African American | 41 | Yes | No | No |
| phchp004v4 | SZA | M | 63 | African American | 20 | Yes | No | No |
| phchp009v1 | SZ | M | 55 | African American | 37 | Yes | No | No |
| phchp009v3 | SZ | M | 56 | African American | 45 | Yes | No | No |
| phchp010v1 | SZA | M | 45 | Caucasian | 44 | Yes | No | No |
| phchp010v2 | SZA | M | 45 | Caucasian | 61 | Yes | No | No |
| phchp010v3 | SZA | M | 45 | Caucasian | 69 | Yes | No | No |
| phchp012v1 | SZA | M | 55 | Caucasian | 32 | Yes | No | No |
| phchp012v2 | SZA | M | 55 | Caucasian | 75 | Yes | No | No |
| phchp012v3 | SZA | M | 55 | Caucasian | 55 | Yes | No | No |
| phchp013v1 | SZA | M | 53 | African American | 20 | Yes | No | No |
| phchp013v3 | SZA | M | 54 | African American | 55 | Yes | No | No |
| phchp016v1 | SZ | M | 54 | African American | 20 | Yes | No | No |
| phchp016v2 | SZ | M | 54 | African American | 55 | Yes | No | No |
| phchp016v3 | SZ | M | 54 | African American | 47 | Yes | No | No |
| phchp019v1 | SZ | M | 50 | African American | 67 | Yes | No | No |
| phchp019v2 | SZ | M | 51 | African American | 56 | Yes | No | No |
| phchp019v3 | SZ | M | 51 | African American | 38 | Yes | No | No |
| phchp019v5 | SZ | M | 59 | African American | 42 | Yes | No | No |
| phchp021v1 | SZA | M | 48 | Hispanic | 21 | Yes | No | No |
| phchp021v2 | SZA | M | 49 | Hispanic | 56 | Yes | No | No |
| phchp021v3 | SZA | M | 49 | Hispanic | 48 | Yes | No | No |
| phchp026v1 | SZA | M | 49 | African American | 47 | Yes | No | No |
| phchp026v2 | SZA | M | 49 | African American | 46 | Yes | No | No |
| phchp026v3 | SZA | M | 49 | African American | 56 | Yes | No | No |
| phchp028v1 | BP | F | 50 | Asian | 23 | Yes | No | No |
| phchp028v2 | BP | F | 50 | Asian | 39 | Yes | No | No |
| phchp031v1 | BP | M | 51 | Caucasian | 39 | Yes | No | No |
| phchp031v2 | BP | M | 51 | Caucasian | 47 | Yes | No | No |
| phchp031v3 | BP | M | 52 | Caucasian | 20 | Yes | No | No |
| phchp035v1 | BP | F | 36 | Caucasian | 36 | Yes | No | No |
| phchp035v2 | BP | F | 37 | Caucasian | 57 | Yes | No | No |
| phchp035v3 | BP | F | 37 | Caucasian | 57 | Yes | No | No |
| phchp038v1 | SZA | M | 58 | African American | 40 | Yes | No | No |
| phchp038v2 | SZA | M | 58 | African American | 43 | Yes | No | No |
| phchp038v3 | SZA | M | 59 | African American | 55 | Yes | No | No |
| phchp040v1 | SZA | M | 50 | Caucasian | 56 | Yes | No | No |
| phchp040v2 | SZA | M | 50 | Caucasian | 38 | Yes | No | No |
| phchp040v3 | SZA | M | 50 | Caucasian | 47 | Yes | No | No |
| phchp042v1 | SZA | M | 43 | Caucasian | 71 | Yes | No | No |
| phchp042v2 | SZA | M | 43 | Caucasian | 22 | Yes | No | No |
| phchp042v3 | SZA | M | 44 | Caucasian | 49 | Yes | No | No |
| phchp046v1 | SZA | M | 45 | Caucasian | 33 | Yes | No | No |
| phchp046v2 | SZA | M | 45 | Caucasian | 27 | Yes | No | No |
| phchp046v3 | SZA | M | 45 | Caucasian | 29 | Yes | No | No |
| phchp048v1 | SZA | M | 56 | African American | 55 | Yes | No | No |
| phchp048v2 | SZA | M | 57 | African American | 41 | Yes | No | No |
| phchp048v3 | SZA | M | 57 | African American | 63 | Yes | No | No |
| phchp049v1 | SZA | M | 46 | Caucasian | 49 | Yes | No | No |
| phchp049v2 | SZA | M | 47 | Caucasian | 31 | Yes | No | No |
| phchp052v1 | SZ | M | 60 | Caucasian | 48 | Yes | No | No |
| phchp052v2 | SZ | M | 60 | Caucasian | 80 | Yes | No | No |
| phchp052v3 | SZ | M | 60 | Caucasian | 49 | Yes | No | No |
| phchp053v1 | BP | M | 58 | Caucasian | 55 | Yes | No | No |
| phchp053v2 | BP | M | 58 | Caucasian | 63 | Yes | No | No |
| phchp053v3 | BP | M | 58 | Caucasian | 37 | Yes | No | No |
| phchp055v1 | BP | F | 46 | Caucasian | 31 | Yes | No | No |
| phchp055v2 | BP | F | 46 | Caucasian | 55 | Yes | No | No |
| phchp055v3 | BP | F | 46 | Caucasian | 55 | Yes | No | No |
| phchp058v1 | SZ | M | 56 | African American | 20 | Yes | No | No |
| phchp058v2 | SZ | M | 56 | African American | 47 | Yes | No | No |
| phchp058v3 | SZ | M | 56 | African American | 47 | Yes | No | No |
| phchp061v1 | SZ | M | 49 | Caucasian | 28 | Yes | No | No |
| phchp061v2 | SZ | M | 49 | Caucasian | 67 | Yes | No | No |
| phchp061v3 | SZ | M | 50 | Caucasian | 56 | Yes | No | No |
| phchp062v1 | SZ | M | 56 | Caucasian | 25 | Yes | No | No |
| phchp062v2 | SZ | M | 56 | Caucasian | 45 | Yes | No | No |
| phchp062v3 | SZ | M | 57 | Caucasian | 29 | Yes | No | No |
| phchp065v1 | SZA | M | 62 | Caucasian | 25 | Yes | No | No |
| phchp065v2 | SZA | M | 62 | Caucasian | 20 | Yes | No | No |
| phchp065v3 | SZA | M | 62 | Caucasian | 33 | Yes | No | No |
| phchp067v1 | BP | M | 39 | Caucasian | 25 | Yes | No | No |
| phchp067v3 | BP | M | 40 | Caucasian | 55 | Yes | No | No |
| phchp069v1 | SZ | M | 47 | Caucasian | 48 | Yes | No | No |
| phchp069v2 | SZ | M | 47 | Caucasian | 56 | Yes | No | No |
| phchp069v3 | SZ | M | 48 | Caucasian | 44 | Yes | No | No |
| phchp070v1 | SZ | M | 52 | African American | 56 | Yes | No | No |
| phchp070v2 | SZ | M | 52 | African American | 75 | Yes | No | No |
| phchp070v3 | SZ | M | 52 | African American | 45 | Yes | No | No |
| phchp070v4 | SZ | M | 56 | African American | 55 | Yes | No | No |
| phchp070v5 | SZ | M | 56 | African American | 45 | Yes | No | No |
| phchp070v6 | SZ | M | 57 | African American | 45 | Yes | No | No |
| phchp072v1 | SZA | M | 60 | Caucasian | 48 | Yes | No | No |
| phchp072v2 | SZA | M | 60 | Caucasian | 55 | Yes | No | No |
| phchp072v3 | SZA | M | 60 | Caucasian | 80 | Yes | No | No |
| phchp073v1 | SZA | M | 50 | Caucasian | 20 | Yes | No | No |
| phchp073v3 | SZA | M | 50 | Caucasian | 72 | Yes | No | No |
| phchp074v1 | SZA | F | 46 | African American | 39 | Yes | No | No |
| phchp074v2 | SZA | F | 46 | African American | 27 | Yes | No | No |
| phchp074v3 | SZA | F | 46 | African American | 42 | Yes | No | No |
| phchp075v1 | SZA | M | 57 | Caucasian | 69 | Yes | No | No |
| phchp075v3 | SZA | M | 58 | Caucasian | 37 | Yes | No | No |
| phchp079v1 | BP | M | 44 | Caucasian | 48 | Yes | No | No |
| phchp079v2 | BP | M | 44 | Caucasian | 55 | Yes | No | No |
| phchp079v3 | BP | M | 45 | Caucasian | 50 | Yes | No | No |
| phchp079v4 | BP | M | 49 | Caucasian | 76 | Yes | No | No |
| phchp079v5 | BP | M | 50 | Caucasian | 47 | Yes | No | No |
| phchp079v6 | BP | M | 50 | Caucasian | 55 | Yes | No | No |
| phchp083v1 | SZ | M | 50 | African American | 44 | Yes | No | No |
| phchp083v2 | SZ | M | 50 | African American | 35 | Yes | No | No |
| phchp083v3 | SZ | M | 51 | African American | 46 | Yes | No | No |
| phchp084v1 | BP | F | 49 | Caucasian | 49 | Yes | No | No |
| phchp084v2 | BP | F | 49 | Caucasian | 65 | Yes | No | No |
| phchp084v3 | BP | F | 50 | Caucasian | 56 | Yes | No | No |
| phchp084v4 | BP | F | 57 | Caucasian | 48 | Yes | No | No |
| phchp085v1 | SZA | M | 57 | Caucasian | 32 | Yes | No | No |
| phchp085v2 | SZA | M | 57 | Caucasian | 43 | Yes | No | No |
| phchp085v3 | SZA | M | 57 | Caucasian | 38 | Yes | No | No |
| phchp086v1 | SZ | M | 49 | Caucasian | 42 | Yes | No | No |
| phchp086v2 | SZ | M | 49 | Caucasian | 35 | Yes | No | No |
| phchp086v3 | SZ | M | 49 | Caucasian | 56 | Yes | No | No |
| phchp087v1 | SZA | M | 65 | Caucasian | 49 | Yes | No | No |
| phchp087v2 | SZA | M | 66 | Caucasian | 65 | Yes | No | No |
| phchp087v3 | SZA | M | 66 | Caucasian | 49 | Yes | No | No |
| phchp088v1 | BP | M | 44 | Caucasian | 20 | Yes | No | No |
| phchp088v2 | BP | M | 45 | Caucasian | 22 | Yes | No | No |
| phchp088v3 | BP | M | 45 | Caucasian | 55 | Yes | No | No |
| phchp088v5 | BP | M | 50 | Caucasian | 20 | Yes | No | No |
| phchp088v6 | BP | M | 51 | Caucasian | 20 | Yes | No | No |
| phchp089v1 | SZA | M | 33 | Caucasian | 31 | Yes | No | No |
| phchp089v2 | SZA | M | 33 | Caucasian | 48 | Yes | No | No |
| phchp089v4 | SZA | M | 38 | Caucasian | 30 | Yes | No | No |
| phchp091v1 | SZA | M | 55 | Caucasian | 55 | Yes | No | No |
| phchp091v3 | SZA | M | 55 | Caucasian | 20 | Yes | No | No |
| phchp093v1 | BP | M | 51 | Caucasian | 20 | Yes | No | No |
| phchp093v2 | BP | M | 51 | Caucasian | 35 | Yes | No | No |
| phchp093v3 | BP | M | 52 | Caucasian | 20 | Yes | No | No |
| phchp093v4 | BP | M | 56 | Caucasian | 39 | Yes | No | No |
| phchp093v5 | BP | M | 57 | Caucasian | 35 | Yes | No | No |
| phchp093v6 | BP | M | 57 | Caucasian | 20 | Yes | No | No |
| phchp099v1 | SZ | M | 49 | Caucasian | 43 | Yes | No | No |
| phchp099v2 | SZ | M | 49 | Caucasian | 54 | Yes | No | No |
| phchp099v3 | SZ | M | 49 | Caucasian | 56 | Yes | No | No |
| phchp106v1 | BP | F | 28 | Mixed | 45 | Yes | No | No |
| phchp106v2 | BP | F | 28 | Mixed | 55 | Yes | No | No |
| phchp106v3 | BP | F | 29 | Mixed | 55 | Yes | No | No |
| phchp108v1 | SZ | M | 42 | Caucasian | 20 | Yes | No | No |
| phchp108v3 | SZ | M | 43 | Caucasian | 55 | Yes | No | No |
| phchp109v1 | BP | M | 22 | Caucasian | 24 | Yes | No | No |
| phchp109v2 | BP | M | 25 | Caucasian | 53 | Yes | No | No |
| phchp112v1 | BP | M | 46 | Caucasian | 55 | Yes | No | No |
| phchp112v2 | BP | M | 46 | Caucasian | 46 | Yes | No | No |
| phchp112v3 | BP | M | 47 | Caucasian | 56 | Yes | No | No |
| phchp117v1 | BP | M | 43 | Caucasian | 37 | Yes | No | No |
| phchp117v2 | BP | M | 43 | Caucasian | 49 | Yes | No | No |
| phchp117v3 | BP | M | 43 | Caucasian | 48 | Yes | No | No |
| phchp118v1 | SZA | M | 46 | African American | 33 | Yes | No | No |
| phchp118v2 | SZA | M | 47 | African American | 48 | Yes | No | No |
| phchp118v4 | SZA | M | 50 | African American | 32 | Yes | No | No |
| phchp119v2 | SZA | M | 56 | African American | 67 | Yes | No | No |
| phchp119v3 | SZA | M | 56 | African American | 39 | Yes | No | No |
| phchp121v1 | MOOD | M | 55 | Caucasian | 20 | Yes | No | No |
| phchp121v2 | MOOD | M | 56 | Caucasian | 39 | Yes | No | No |
| phchp121v3 | MOOD | M | 56 | Caucasian | 20 | Yes | No | No |
| phchp124v1 | BP | M | 53 | Caucasian | 40 | Yes | No | No |
| phchp124v2 | BP | M | 54 | Caucasian | 55 | Yes | No | No |
| phchp127v1 | SZA | F | 58 | Caucasian | 64 | Yes | No | No |
| phchp127v2 | SZA | F | 58 | Caucasian | 55 | Yes | No | No |
| phchp127v3 | SZA | F | 59 | Caucasian | 43 | Yes | No | No |
| phchp128v1 | BP | M | 45 | Caucasian | 31 | Yes | No | No |
| phchp128v2 | BP | M | 45 | Caucasian | 41 | Yes | No | No |
| phchp129v1 | SZA | M | 22 | Caucasian | 44 | Yes | No | No |
| phchp129v3 | SZA | M | 27 | Caucasian | 20 | Yes | No | No |
| phchp131v1 | SZ | F | 54 | African American | 20 | Yes | No | No |
| phchp131v2 | SZ | F | 55 | African American | 40 | Yes | No | No |
| phchp131v3 | SZ | F | 56 | African American | 21 | Yes | No | No |
| phchp132v1 | BP | M | 51 | Caucasian | 20 | Yes | No | No |
| phchp132v2 | BP | M | 51 | Caucasian | 20 | Yes | No | No |
| phchp132v3 | BP | M | 52 | Caucasian | 74 | Yes | No | No |
| phchp132v4 | BP | M | 54 | Caucasian | 20 | Yes | No | No |
| phchp132v5 | BP | M | 54 | Caucasian | 47 | Yes | No | No |
| phchp132v6 | BP | M | 55 | Caucasian | 31 | Yes | No | No |
| phchp134v1 | BP | M | 59 | Caucasian | 32 | Yes | No | No |
| phchp134v2 | BP | M | 59 | Caucasian | 62 | Yes | No | No |
| phchp134v3 | BP | M | 59 | Caucasian | 50 | Yes | No | No |
| phchp134v4 | BP | M | 61 | Caucasian | 50 | Yes | No | No |
| phchp134v5 | BP | M | 62 | Caucasian | 50 | Yes | No | No |
| phchp134v6 | BP | M | 62 | Caucasian | 55 | Yes | No | No |
| phchp136v1 | BP | M | 41 | Caucasian | 26 | Yes | No | No |
| phchp136v2 | BP | M | 41 | Caucasian | 55 | Yes | No | No |
| phchp136v3 | BP | M | 41 | Caucasian | 48 | Yes | No | No |
| phchp138v1 | MOOD | M | 59 | African American | 55 | Yes | No | No |
| phchp138v2 | MOOD | M | 59 | African American | 38 | Yes | No | No |
| phchp138v3 | MOOD | M | 59 | African American | 37 | Yes | No | No |
| phchp140v1 | BP | M | 38 | Caucasian | 22 | Yes | No | No |
| phchp140v2 | BP | M | 38 | Caucasian | 47 | Yes | No | No |
| phchp140v3 | BP | M | 38 | Caucasian | 55 | Yes | No | No |
| phchp140v4 | BP | M | 40 | Caucasian | 37 | Yes | No | No |
| phchp141v1 | BP | F | 47 | Caucasian | 55 | Yes | No | No |
| phchp141v2 | BP | F | 47 | Caucasian | 68 | Yes | No | No |
| phchp141v3 | BP | F | 47 | Caucasian | 57 | Yes | No | No |
| phchp142v1 | BP | M | 55 | Caucasian | 65 | Yes | No | No |
| phchp142v2 | BP | M | 55 | Caucasian | 40 | Yes | No | No |
| phchp142v3 | BP | M | 55 | Caucasian | 41 | Yes | No | No |
| phchp142v4 | BP | M | 57 | Caucasian | 50 | Yes | No | No |
| phchp142v5 | BP | M | 57 | Caucasian | 55 | Yes | No | No |
| phchp142v6 | BP | M | 58 | Caucasian | 50 | Yes | No | No |
| phchp149v1 | MOOD | M | 45 | Caucasian | 39 | Yes | No | No |
| phchp149v2 | MOOD | M | 45 | Caucasian | 20 | Yes | No | No |
| phchp150v1 | SZA | M | 61 | Caucasian | 49 | Yes | No | No |
| phchp150v2 | SZA | M | 61 | Caucasian | 43 | Yes | No | No |
| phchp150v3 | SZA | M | 62 | Caucasian | 20 | Yes | No | No |
| phchp151v1 | SZ | M | 24 | Caucasian | 24 | Yes | No | No |
| phchp151v2 | SZ | M | 24 | Caucasian | 53 | Yes | No | No |
| phchp151v3 | SZ | M | 24 | Caucasian | 36 | Yes | No | No |
| phchp153v1 | BP | M | 55 | Caucasian | 66 | Yes | No | No |
| phchp153v2 | BP | M | 55 | Caucasian | 64 | Yes | No | No |
| phchp153v3 | BP | M | 56 | Caucasian | 48 | Yes | No | No |
| phchp153v4 | BP | M | 57 | Caucasian | 64 | Yes | No | No |
| phchp153v6 | BP | M | 58 | Caucasian | 65 | Yes | No | No |
| phchp154v1 | PSYCH | M | 51 | African American | 39 | Yes | No | No |
| phchp154v2 | PSYCH | M | 51 | African American | 20 | Yes | No | No |
| phchp154v3 | PSYCH | M | 52 | African American | 28 | Yes | No | No |
| phchp155v1 | MDD | M | 37 | Caucasian | 55 | Yes | No | No |
| phchp155v2 | MDD | M | 37 | Caucasian | 74 | Yes | No | No |
| phchp157v1 | BP | M | 57 | African American | 55 | Yes | No | No |
| phchp157v2 | BP | M | 57 | African American | 71 | Yes | No | No |
| phchp157v3 | BP | M | 58 | African American | 50 | Yes | No | No |
| phchp160v1 | SZA | F | 41 | Caucasian | 38 | Yes | No | No |
| phchp160v2 | SZA | F | 41 | Caucasian | 55 | Yes | No | No |
| phchp160v3 | SZA | F | 41 | Caucasian | 47 | Yes | No | No |
| phchp161v1 | MDD | M | 54 | African American | 37 | Yes | No | No |
| phchp161v2 | MDD | M | 54 | African American | 48 | Yes | No | No |
| phchp161v3 | MDD | M | 54 | African American | 40 | Yes | No | No |
| phchp162v1 | MDD | M | 57 | Caucasian | 70 | Yes | No | No |
| phchp162v2 | MDD | M | 57 | Caucasian | 35 | Yes | No | No |
| phchp162v3 | MDD | M | 57 | Caucasian | 55 | Yes | No | No |
| phchp166v1 | BP | M | 56 | Caucasian | 68 | Yes | No | No |
| phchp166v2 | BP | M | 56 | Caucasian | 48 | Yes | No | No |
| phchp166v3 | BP | M | 56 | Caucasian | 55 | Yes | No | No |
| phchp166v4 | BP | M | 58 | Caucasian | 55 | Yes | No | No |
| phchp166v5 | BP | M | 58 | Caucasian | 47 | Yes | No | No |
| phchp166v6 | BP | M | 59 | Caucasian | 80 | Yes | No | No |
| phchp168v1 | MDD | M | 48 | African American | 35 | Yes | No | No |
| phchp168v2 | MDD | M | 48 | African American | 39 | Yes | No | No |
| phchp168v3 | MDD | M | 49 | African American | 47 | Yes | No | No |
| phchp172v1 | BP | F | 24 | Caucasian | 80 | Yes | No | No |
| phchp172v2 | BP | F | 24 | Caucasian | 62 | Yes | No | No |
| phchp172v3 | BP | F | 25 | Caucasian | 53 | Yes | No | No |
| phchp176v1 | SZ | M | 23 | African American | 80 | Yes | No | No |
| phchp176v2 | SZ | M | 24 | African American | 45 | Yes | No | No |
| phchp179v1 | BP | M | 36 | Caucasian | 67 | Yes | No | No |
| phchp179v2 | BP | M | 37 | Caucasian | 41 | Yes | No | No |
| phchp179v4 | BP | M | 37 | Caucasian | 41 | Yes | No | No |
| phchp181v1 | BP | F | 28 | Caucasian | 48 | Yes | No | No |
| phchp181v3 | BP | F | 28 | Caucasian | 39 | Yes | No | No |
| phchp181v4 | BP | F | 29 | Caucasian | 46 | Yes | No | No |
| phchp183v1 | BP | M | 48 | Caucasian | 80 | Yes | No | No |
| phchp183v2 | BP | M | 48 | Caucasian | 56 | Yes | No | No |
| phchp184v1 | BP | M | 64 | Caucasian | 57 | Yes | No | No |
| phchp184v2 | BP | M | 64 | Caucasian | 47 | Yes | No | No |
| phchp184v3 | BP | M | 64 | Caucasian | 40 | Yes | No | No |
| phchp185v1 | SZA | M | 51 | African American | 32 | Yes | No | No |
| phchp185v2 | SZA | M | 51 | African American | 42 | Yes | No | No |
| phchp185v3 | SZA | M | 52 | African American | 20 | Yes | No | No |
| phchp186v1 | BP | M | 43 | Caucasian | 55 | Yes | No | No |
| phchp186v2 | BP | M | 44 | Caucasian | 48 | Yes | No | No |
| phchp186v3 | BP | M | 44 | Caucasian | 50 | Yes | No | No |
| phchp186v4 | BP | M | 46 | Caucasian | 61 | Yes | No | No |
| phchp186v5 | BP | M | 48 | Caucasian | 63 | Yes | No | No |
| phchp187v1 | SZ | M | 49 | African American | 20 | Yes | No | No |
| phchp187v2 | SZ | M | 49 | African American | 42 | Yes | No | No |
| phchp188v1 | PSYCH | M | 48 | African American | 29 | Yes | No | No |
| phchp188v2 | PSYCH | M | 49 | African American | 20 | Yes | No | No |
| phchp188v3 | PSYCH | M | 54 | African American | 37 | Yes | No | No |
| phchp189v1 | SZ | M | 25 | Caucasian | 20 | Yes | No | No |
| phchp189v3 | SZ | M | 25 | Caucasian | 41 | Yes | No | No |
| phchp190v1 | BP | M | 49 | Caucasian | 25 | Yes | No | No |
| phchp190v2 | BP | M | 49 | Caucasian | 47 | Yes | No | No |
| phchp190v3 | BP | M | 50 | Caucasian | 47 | Yes | No | No |
| phchp190v4 | BP | M | 54 | Caucasian | 35 | Yes | No | No |
| phchp192v1 | SZA | M | 55 | African American | 45 | Yes | No | No |
| phchp192v2 | SZA | M | 56 | African American | 31 | Yes | No | No |
| phchp192v3 | SZA | M | 56 | African American | 37 | Yes | No | No |
| phchp193v1 | BP | M | 39 | Hispanic | 70 | Yes | No | No |
| phchp193v3 | BP | M | 39 | Hispanic | 55 | Yes | No | No |
| phchp193v4 | BP | M | 40 | Hispanic | 49 | Yes | No | No |
| phchp194v1 | MDD | M | 47 | Caucasian | 47 | Yes | No | No |
| phchp194v2 | MDD | M | 47 | Caucasian | 40 | Yes | No | No |
| phchp194v3 | MDD | M | 47 | Caucasian | 22 | Yes | No | No |
| phchp195v1 | SZ | M | 52 | Caucasian | 56 | Yes | No | No |
| phchp195v2 | SZ | M | 53 | Caucasian | 23 | Yes | No | No |
| phchp195v3 | SZ | M | 53 | Caucasian | 39 | Yes | No | No |
| phchp196v1 | MDD | M | 56 | African American | 55 | Yes | No | No |
| phchp196v3 | MDD | M | 57 | African American | 20 | Yes | No | No |
| phchp199v1 | SZ | M | 49 | African American | 42 | Yes | No | No |
| phchp199v2 | SZ | M | 49 | African American | 66 | Yes | No | No |
| phchp199v3 | SZ | M | 50 | African American | 47 | Yes | No | No |
| phchp200v1 | MDD | M | 56 | Caucasian | 48 | Yes | No | No |
| phchp200v2 | MDD | M | 57 | Caucasian | 62 | Yes | No | No |
| phchp200v3 | MDD | M | 57 | Caucasian | 55 | Yes | No | No |
| phchp204v1 | BP | F | 49 | Caucasian | 47 | Yes | No | No |
| phchp204v2 | BP | F | 49 | Caucasian | 56 | Yes | No | No |
| phchp204v3 | BP | F | 49 | Caucasian | 45 | Yes | No | No |
| phchp208v1 | MDD | M | 56 | African American | 47 | Yes | No | No |
| phchp208v2 | MDD | M | 56 | African American | 46 | Yes | No | No |
| phchp208v3 | MDD | M | 58 | African American | 30 | Yes | No | No |
| phchp209v2 | PTSD | M | 54 | African American | 41 | Yes | No | No |
| phchp209v3 | PTSD | M | 54 | African American | 25 | Yes | No | No |
| phchp210v1 | BP | M | 43 | Caucasian | 35 | Yes | No | No |
| phchp210v2 | BP | M | 43 | Caucasian | 39 | Yes | No | No |
| phchp210v3 | BP | M | 44 | Caucasian | 26 | Yes | No | No |
| phchp211v1 | SZ | M | 62 | Caucasian | 39 | Yes | No | No |
| phchp211v2 | SZ | M | 62 | Caucasian | 30 | Yes | No | No |
| phchp211v3 | SZ | M | 62 | Caucasian | 30 | Yes | No | No |
| phchp213v1 | PTSD | M | 62 | Caucasian | 46 | Yes | No | No |
| phchp213v2 | PTSD | M | 62 | Caucasian | 57 | Yes | No | No |
| phchp213v3 | PTSD | M | 62 | Caucasian | 55 | Yes | No | No |
| phchp214v1 | PTSD | F | 52 | Caucasian | 33 | Yes | No | No |
| phchp214v2 | PTSD | F | 53 | Caucasian | 55 | Yes | No | No |
| phchp214v3 | PTSD | F | 57 | Caucasian | 39 | Yes | No | No |
| phchp215v1 | PTSD | F | 58 | Caucasian | 20 | Yes | No | No |
| phchp215v2 | PTSD | F | 58 | Caucasian | 41 | Yes | No | No |
| phchp215v3 | PTSD | F | 61 | Caucasian | 20 | Yes | No | No |
| phchp216v1 | MOOD | M | 50 | African American | 56 | Yes | No | No |
| phchp216v2 | MOOD | M | 51 | African American | 45 | Yes | No | No |
| phchp216v3 | MOOD | M | 51 | African American | 46 | Yes | No | No |
| phchp219v1 | BP | M | 61 | Caucasian | 55 | Yes | No | No |
| phchp219v2 | BP | M | 61 | Caucasian | 55 | Yes | No | No |
| phchp219v3 | BP | M | 62 | Caucasian | 34 | Yes | No | No |
| phchp221v1 | MDD | M | 51 | African American | 35 | Yes | No | No |
| phchp221v2 | MDD | M | 51 | African American | 47 | Yes | No | No |
| phchp221v3 | MDD | M | 52 | African American | 56 | Yes | No | No |
| phchp227v1 | MDD | M | 55 | Caucasian | 64 | Yes | No | No |
| phchp227v2 | MDD | M | 55 | Caucasian | 37 | Yes | No | No |
| phchp227v3 | MDD | M | 55 | Caucasian | 33 | Yes | No | No |
| phchp235v1 | MDD | M | 54 | African American | 23 | Yes | No | No |
| phchp235v2 | MDD | M | 55 | African American | 21 | Yes | No | No |
| phchp235v3 | MDD | M | 55 | African American | 29 | Yes | No | No |
| phchp238v1 | MDD | M | 62 | Caucasian | 34 | Yes | No | No |
| phchp238v2 | MDD | M | 63 | Caucasian | 67 | Yes | No | No |
| phchp238v3 | MDD | M | 63 | Caucasian | 55 | Yes | No | No |
| phchp240v1 | MDD | F | 55 | Caucasian | 21 | Yes | No | No |
| phchp240v2 | MDD | F | 55 | Caucasian | 55 | Yes | No | No |
| phchp240v3 | MDD | F | 56 | Caucasian | 55 | Yes | No | No |
| phchp242v1 | MDD | M | 55 | African American | 32 | Yes | No | No |
| phchp242v2 | MDD | M | 57 | African American | 55 | Yes | No | No |
| phchp242v3 | MDD | M | 57 | African American | 43 | Yes | No | No |
| phchp253v1 | BP | M | 25 | Caucasian | 45 | Yes | No | No |
| phchp253v2 | BP | M | 26 | Caucasian | 45 | Yes | No | No |
| phchp253v3 | BP | M | 26 | Caucasian | 20 | Yes | No | No |
| phchp254v1 | MDD | F | 49 | Caucasian | 38 | Yes | No | No |
| phchp254v2 | MDD | F | 49 | Caucasian | 80 | Yes | No | No |
| phchp254v3 | MDD | F | 50 | Caucasian | 47 | Yes | No | No |
| phchp258v1 | BP | F | 52 | Caucasian | 49 | Yes | No | No |
| phchp258v2 | BP | F | 52 | Caucasian | 35 | Yes | No | No |
| phchp258v3 | BP | F | 54 | Caucasian | 47 | Yes | No | No |
| phchp259v1 | MDD | M | 56 | Caucasian | 44 | Yes | No | No |
| phchp259v2 | MDD | M | 57 | Caucasian | 46 | Yes | No | No |
| phchp259v3 | MDD | M | 57 | Caucasian | 35 | Yes | No | No |
| phchp270v1 | BP | M | 36 | Caucasian | 32 | Yes | No | No |
| phchp270v2 | BP | M | 36 | Caucasian | 48 | Yes | No | No |
| phchp270v3 | BP | M | 41 | Caucasian | 49 | Yes | No | No |
| phchp270v4 | BP | M | 41 | Caucasian | 55 | Yes | No | No |
| phchp270v5 | BP | M | 42 | Caucasian | 55 | Yes | No | No |
| phchp270v6 | BP | M | 44 | Caucasian | 37 | Yes | No | No |
| phchp273v1 | BP | M | 27 | Caucasian | 21 | Yes | No | No |
| phchp273v2 | BP | M | 28 | Caucasian | 42 | Yes | No | No |
| phchp274v1 | BP | M | 48 | Caucasian | 47 | Yes | No | No |
| phchp274v2 | BP | M | 48 | Caucasian | 33 | Yes | No | No |
| phchp274v3 | BP | M | 48 | Caucasian | 35 | Yes | No | No |
| phchp274v4 | BP | M | 50 | Caucasian | 55 | Yes | No | No |
| phchp275v1 | SZ | M | 63 | Caucasian | 75 | Yes | No | No |
| phchp275v3 | SZ | M | 63 | Caucasian | 20 | Yes | No | No |
| phchp276v1 | SZ | M | 59 | African American | 44 | Yes | No | No |
| phchp276v2 | SZ | M | 59 | African American | 35 | Yes | No | No |
| phchp276v4 | SZ | M | 61 | African American | 73 | Yes | No | No |
| phchp277v1 | SZ | M | 49 | Caucasian | 47 | Yes | No | No |
| phchp277v2 | SZ | M | 50 | Caucasian | 35 | Yes | No | No |
| phchp277v3 | SZ | M | 50 | Caucasian | 56 | Yes | No | No |
| phchp277v4 | SZ | M | 52 | Caucasian | 67 | Yes | No | No |
| phchp277v5 | SZ | M | 52 | Caucasian | 45 | Yes | No | No |
| phchp279v1 | SZ | M | 60 | African American | 43 | Yes | No | No |
| phchp279v2 | SZ | M | 61 | African American | 21 | Yes | No | No |
| phchp279v4 | SZ | M | 61 | African American | 69 | Yes | No | No |
| phchp282v1 | SZ | M | 56 | Caucasian | 55 | Yes | No | No |
| phchp282v3 | SZ | M | 57 | Caucasian | 33 | Yes | No | No |
| phchp282v4 | SZ | M | 58 | Caucasian | 55 | Yes | No | No |
| phchp282v5 | SZ | M | 59 | Caucasian | 48 | Yes | No | No |
| phchp287v1 | SZA | M | 59 | Caucasian | 43 | Yes | No | No |
| phchp287v2 | SZA | M | 60 | Caucasian | 55 | Yes | No | No |
| phchp287v3 | SZA | M | 60 | Caucasian | 64 | Yes | No | No |
| phchp290v1 | BP | M | 55 | Caucasian | 45 | Yes | No | No |
| phchp290v2 | BP | M | 55 | Caucasian | 37 | Yes | No | No |
| phchp290v3 | BP | M | 55 | Caucasian | 41 | Yes | No | No |
| phchp292v1 | BP | M | 42 | Caucasian | 38 | Yes | No | No |
| phchp292v2 | BP | M | 42 | Caucasian | 47 | Yes | No | No |
| phchp292v3 | BP | M | 42 | Caucasian | 42 | Yes | No | No |
| phchp297v1 | SZA | M | 54 | African American | 31 | Yes | No | No |
| phchp297v2 | SZA | M | 55 | African American | 64 | Yes | No | No |
| phchp297v4 | SZA | M | 57 | African American | 45 | Yes | No | No |
| phchp298v1 | SZA | M | 56 | Caucasian | 40 | Yes | No | No |
| phchp298v2 | SZA | M | 56 | Caucasian | 55 | Yes | No | No |
| phchp298v3 | SZA | M | 56 | Caucasian | 48 | Yes | No | No |
| phchp299v1 | PTSD | M | 54 | Caucasian | 37 | Yes | No | No |
| phchp299v2 | PTSD | M | 54 | Caucasian | 62 | Yes | No | No |
| phchp299v4 | PTSD | M | 55 | Caucasian | 31 | Yes | No | No |
| phchp299v5 | PTSD | M | 56 | Caucasian | 55 | Yes | No | No |
| phchp300v1 | BP | M | 47 | Caucasian | 55 | Yes | No | No |
| phchp300v2 | BP | M | 47 | Caucasian | 55 | Yes | No | No |
| phchp300v3 | BP | M | 48 | Caucasian | 47 | Yes | No | No |
| phchp300v4 | BP | M | 49 | Caucasian | 56 | Yes | No | No |
| phchp300v5 | BP | M | 50 | Caucasian | 80 | Yes | No | No |
| phchp300v6 | BP | M | 50 | Caucasian | 55 | Yes | No | No |
| phchp302v1 | BP | M | 61 | Caucasian | 26 | Yes | No | No |
| phchp302v2 | BP | M | 61 | Caucasian | 50 | Yes | No | No |
| phchp302v3 | BP | M | 61 | Caucasian | 55 | Yes | No | No |
| phchp302v5 | BP | M | 62 | Caucasian | 55 | Yes | No | No |
| phchp302v6 | BP | M | 63 | Caucasian | 55 | Yes | No | No |
| phchp308v1 | SZA | M | 47 | African American | 31 | Yes | No | No |
| phchp308v2 | SZA | M | 47 | African American | 26 | Yes | No | No |
| phchp308v3 | SZA | M | 48 | African American | 39 | Yes | No | No |
| phchp308v4 | SZA | M | 49 | African American | 31 | Yes | No | No |
| phchp309v1 | PTSD | F | 27 | Caucasian | 48 | Yes | No | No |
| phchp309v2 | PTSD | F | 28 | Caucasian | 40 | Yes | No | No |
| phchp309v3 | PTSD | F | 28 | Caucasian | 55 | Yes | No | No |
| phchp310v1 | MOOD | M | 54 | African American | 28 | Yes | No | No |
| phchp310v2 | MOOD | M | 54 | African American | 55 | Yes | No | No |
| phchp310v3 | MOOD | M | 54 | African American | 55 | Yes | No | No |
| phchp311v1 | MDD | F | 60 | African American | 32 | Yes | No | No |
| phchp311v2 | MDD | F | 61 | African American | 63 | Yes | No | No |
| phchp311v3 | MDD | F | 61 | African American | 40 | Yes | No | No |
| phchp312v1 | BP | M | 64 | Caucasian | 57 | Yes | No | No |
| phchp312v2 | BP | M | 65 | Caucasian | 41 | Yes | No | No |
| phchp312v3 | BP | M | 65 | caucasian | 66 | Yes | No | No |
| phchp313v1 | PTSD | M | 46 | African American | 20 | Yes | No | No |
| phchp313v3 | PTSD | M | 46 | African American | 55 | Yes | No | No |
| phchp316v1 | BP | M | 50 | Caucasian | 56 | Yes | No | No |
| phchp316v2 | BP | M | 50 | Caucasian | 49 | Yes | No | No |
| phchp316v3 | BP | M | 51 | Caucasian | 56 | Yes | No | No |
| phchp316v4 | BP | M | 51 | Caucasian | 55 | Yes | No | No |
| phchp316v5 | BP | M | 51 | Caucasian | 80 | Yes | No | No |
| phchp316v6 | BP | M | 52 | Caucasian | 20 | Yes | No | No |
| phchp318v1 | MDD | F | 57 | Caucasian | 55 | Yes | No | No |
| phchp318v2 | MDD | F | 57 | Caucasian | 43 | Yes | No | No |
| phchp318v3 | MDD | F | 58 | Caucasian | 40 | Yes | No | No |
| phchp319v1 | PTSD | M | 42 | African American | 55 | Yes | No | No |
| phchp319v3 | PTSD | M | 43 | African American | 37 | Yes | No | No |
| phchp319v5 | PTSD | M | 44 | African American | 63 | Yes | No | No |
| phchp319v6 | PTSD | M | 44 | African American | 55 | Yes | No | No |
| phchp320v1 | BP | M | 58 | African American | 20 | Yes | No | No |
| phchp320v2 | BP | M | 58 | African American | 24 | Yes | No | No |
| phchp325v1 | PTSD | M | 44 | Caucasian | 22 | Yes | No | No |
| phchp325v3 | PTSD | M | 44 | Caucasian | 55 | Yes | No | No |
| phchp325v4 | PTSD | M | 46 | Caucasian | 40 | Yes | No | No |
| phchp328v1 | MDD | F | 37 | Caucasian | 49 | Yes | No | No |
| phchp328v3 | MDD | F | 38 | Caucasian | 22 | Yes | No | No |
| phchp329v2 | SZA | M | 50 | African American | 35 | Yes | No | No |
| phchp329v3 | SZA | M | 51 | African American | 44 | Yes | No | No |
| phchp332v1 | SZA | F | 47 | African American | 47 | Yes | No | No |
| phchp332v2 | SZA | F | 48 | African American | 64 | Yes | No | No |
| phchp333v1 | PTSD | M | 38 | Caucasian | 61 | Yes | No | No |
| phchp333v2 | PTSD | M | 38 | Caucasian | 48 | Yes | No | No |
| phchp333v3 | PTSD | M | 39 | Caucasian | 64 | Yes | No | No |
| phchp333v4 | PTSD | M | 39 | Caucasian | 62 | Yes | No | No |
| phchp334v1 | BP | F | 50 | Caucasian | 20 | Yes | No | No |
| phchp334v2 | BP | F | 50 | Caucasian | 38 | Yes | No | No |
| phchp334v3 | BP | F | 51 | Caucasian | 31 | Yes | No | No |
| phchp338v1 | BP | F | 51 | Caucasian | 39 | Yes | No | No |
| phchp338v2 | BP | F | 51 | Caucasian | 28 | Yes | No | No |
| phchp338v3 | BP | F | 51 | Caucasian | 28 | Yes | No | No |
| phchp338v5 | BP | F | 52 | Caucasian | 56 | Yes | No | No |
| phchp342v1 | MDD | M | 52 | Caucasian | 48 | Yes | No | No |
| phchp342v2 | MDD | M | 52 | Caucasian | 56 | Yes | No | No |
| phchp342v3 | MDD | M | 52 | Caucasian | 40 | Yes | No | No |
| phchp345v1 | PTSD | M | 33 | Caucasian | 22 | Yes | No | No |
| phchp345v2 | PTSD | M | 34 | Caucasian | 23 | Yes | No | No |
| phchp345v3 | PTSD | M | 34 | Caucasian | 57 | Yes | No | No |
| phchp346v1 | PTSD | F | 36 | African American | 28 | Yes | No | No |
| phchp346v2 | PTSD | F | 37 | African American | 35 | Yes | No | No |
| phchp346v3 | PTSD | F | 37 | African American | 20 | Yes | No | No |
| phchp349v1 | PSYCH | M | 58 | Caucasian | 43 | Yes | No | No |
| phchp349v2 | PSYCH | M | 58 | Caucasian | 78 | Yes | No | No |
| phchp349v3 | PSYCH | M | 59 | Caucasian | 47 | Yes | No | No |
| phchp350v2 | BP | M | 48 | Caucasian | 35 | Yes | No | No |
| phchp350v3 | BP | M | 49 | Caucasian | 25 | Yes | No | No |
| phchp350v4 | BP | M | 49 | Caucasian | 47 | Yes | No | No |
| phchp355v1 | MDD | F | 50 | Caucasian | 83 | Yes | No | No |
| phchp355v2 | MDD | F | 50 | Caucasian | 20 | Yes | No | No |
| phchp358v1 | PTSD | M | 52 | Hispanic | 32 | Yes | No | No |
| phchp358v2 | PTSD | M | 52 | Hispanic | 23 | Yes | No | No |
| phchp358v3 | PTSD | M | 53 | Hispanic | 45 | Yes | No | No |
| phchp359v1 | PTSD | F | 56 | Caucasian | 46 | Yes | No | No |
| phchp359v2 | PTSD | F | 56 | Caucasian | 63 | Yes | No | No |
| phchp359v3 | PTSD | F | 57 | Caucasian | 55 | Yes | No | No |
| phchp362v1 | MDD | M | 54 | Caucasian | 55 | Yes | No | No |
| phchp362v2 | MDD | M | 54 | Caucasian | 56 | Yes | No | No |
| phchp362v3 | MDD | M | 55 | Caucasian | 25 | Yes | No | No |
| phchp363v1 | MDD | M | 46 | African American | 39 | Yes | No | No |
| phchp363v2 | MDD | M | 48 | African American | 28 | Yes | No | No |
| phchp367v1 | BP | M | 48 | Caucasian | 20 | Yes | No | No |
| phchp367v2 | BP | M | 49 | Caucasian | 46 | Yes | No | No |

| **B. Independent Test Cohorts (n=127) (238 visits)** | | | | | | | | | | | | |
| --- | --- | --- | --- | --- | --- | --- | --- | --- | --- | --- | --- | --- |
| **Participant ID visit number** | **Diagnosis** | **Gender (M/F)** | **Age at testing (Years)** | **Ethnicity** | **Memory Retention** | **Discovery Cohort** | **Test Cohort Memory Retention State** | **Test Cohort Future Neuropsych Postitive Testing** | **Length of follow-up (days)** | **Time to Future Neuropsych Positive visits** | **Neuropsych Test Date** | **Neuropsych Test Results** |
| phchp027v1 | SZA | M | 40 | Caucasian | 42 | No | Yes | Yes | 4503 | 4299 | 5/10/2018 | 3 = other cognitive disorder but not MCI or ADRD |
| phchp205v1 | PTSD | F | 54 | Caucasian | 48 | No | Yes | Yes | 2586 | 559 | 5/13/2013 | 3 = other cognitive disorder but not MCI or ADRD |
| phchp205v2 | PTSD | F | 54 | Caucasian | 55 | No | Yes | Yes | 2480 | 453 | 5/13/2013 | 3 = other cognitive disorder but not MCI or ADRD |
| phchp205v3 | PTSD | F | 54 | Caucasian | 48 | No | Yes | Yes | 2388 | 361 | 5/13/2013 | 3 = other cognitive disorder but not MCI or ADRD |
| phchp098v1 | SZ | M | 59 | African American | 45 | No | Yes | Yes | 3417 | 2638 | 10/12/2016 | 2 = ADRD |
| phchp039v1 | BP | M | 52 | Caucasian | 47 | No | Yes | Yes | 4393 | 674 | 9/24/2008 | 1 = MCI |
| phchp039v3 | BP | M | 52 | Caucasian | 56 | No | Yes | Yes | 4155 | 436 | 9/24/2008 | 1 = MCI |
| phchp165v1 | SZ | M | 60 | African American | 37 | No | Yes | Yes | 2800 | 2206 | 4/15/2017 | 1 = MCI |
| phchp165v2 | SZ | M | 60 | African American | 43 | No | Yes | Yes | 2709 | 2115 | 4/15/2017 | 1 = MCI |
| phchp165v3 | SZ | M | 61 | African American | 36 | No | Yes | Yes | 2618 | 2024 | 4/15/2017 | 1 = MCI |
| phchp289v1 | PTSD | F | 50 | Caucasian | 20 | No | Yes | Yes | 2247 | 1043 | 8/14/2015 | 1 = MCI |
| phchp006v1 | SZA | M | 52 | African American | 44 | No | Yes | Yes | 4651 | 4651 |  |  |
| phchp006v2 | SZA | M | 52 | African American | 44 | No | Yes | Yes | 4566 | 4566 |  |  |
| phchp017v2 | SZA | M | 53 | African American | 55 | No | Yes | Yes | 4459 | 4459 |  |  |
| phchp017v3 | SZA | M | 54 | African American | 47 | No | Yes | Yes | 4288 | 4288 |  |  |
| phchp020v1 | BP | M | 62 | Caucasian | 43 | No | Yes | Yes | 4540 | 4540 |  |  |
| phchp020v2 | BP | M | 62 | Caucasian | 43 | No | Yes | Yes | 4459 | 4459 |  |  |
| phchp020v3 | BP | M | 63 | Caucasian | 50 | No | Yes | Yes | 4322 | 4322 |  |  |
| phchp041v1 | SZ | M | 62 | African American | 55 | No | Yes | Yes | 4375 | 4375 |  |  |
| phchp047v1 | SZA | M | 57 | African American | 43 | No | Yes | Yes | 4260 | 4260 |  |  |
| phchp047v2 | SZA | M | 57 | African American | 37 | No | Yes | Yes | 4168 | 4168 |  |  |
| phchp047v3 | SZA | M | 58 | African American | 32 | No | Yes | Yes | 4076 | 4076 |  |  |
| phchp051v1 | SZA | M | 52 | Caucasian | 31 | No | Yes | Yes | 4244 | 4244 |  |  |
| phchp060v1 | SZ | M | 62 | Caucasian | 20 | No | Yes | Yes | 4186 | 4186 |  |  |
| phchp068v1 | SZA | M | 57 | African American | 45 | No | Yes | Yes | 4118 | 4118 |  |  |
| phchp068v2 | SZA | M | 57 | African American | 39 | No | Yes | Yes | 3987 | 3987 |  |  |
| phchp068v3 | SZA | M | 57 | African American | 43 | No | Yes | Yes | 3892 | 3892 |  |  |
| phchp081v1 | SZA | M | 53 | African American | 46 | No | Yes | Yes | 3990 | 3990 |  |  |
| phchp081v3 | SZA | M | 53 | African American | 55 | No | Yes | Yes | 3768 | 3768 |  |  |
| phchp096v1 | SZ | M | 55 | African American | 35 | No | Yes | Yes | 3447 | 3447 |  |  |
| phchp096v3 | SZ | M | 56 | African American | 41 | No | Yes | Yes | 3273 | 3273 |  |  |
| phchp096v4 | SZ | M | 58 | African American | 35 | No | Yes | Yes | 2566 | 2566 |  |  |
| phchp101v1 | SZA | M | 74 | Caucasian | 21 | No | Yes | Yes | 3364 | 3364 |  |  |
| phchp105v1 | SZA | M | 59 | Caucasian | 64 | No | Yes | Yes | 3321 | 3321 |  |  |
| phchp114v1 | SZA | M | 54 | African American | 20 | No | Yes | Yes | 3067 | 3067 |  |  |
| phchp115v1 | BP | M | 67 | Caucasian | 47 | No | Yes | Yes | 3165 | 3165 |  |  |
| phchp115v2 | BP | M | 67 | Caucasian | 42 | No | Yes | Yes | 3062 | 3062 |  |  |
| phchp115v3 | BP | M | 68 | Caucasian | 45 | No | Yes | Yes | 2961 | 2961 |  |  |
| phchp120v1 | SZ | M | 51 | Caucasian | 80 | No | Yes | Yes | 3122 | 3122 |  |  |
| phchp120v2 | SZ | M | 51 | Caucasian | 80 | No | Yes | Yes | 3038 | 3038 |  |  |
| phchp120v3 | SZ | M | 51 | Caucasian | 80 | No | Yes | Yes | 2947 | 2947 |  |  |
| phchp122v1 | BP | M | 51 | Caucasian | 56 | No | Yes | Yes | 3027 | 3027 |  |  |
| phchp122v2 | BP | M | 51 | Caucasian | 49 | No | Yes | Yes | 2948 | 2948 |  |  |
| phchp133v1 | SZ | M | 55 | Caucasian | 49 | No | Yes | Yes | 3006 | 3006 |  |  |
| phchp143v1 | BP | F | 62 | African American | 39 | No | Yes | Yes | 2949 | 2949 |  |  |
| phchp143v2 | BP | F | 63 | African American | 40 | No | Yes | Yes | 2849 | 2849 |  |  |
| phchp143v3 | BP | F | 63 | African American | 36 | No | Yes | Yes | 2760 | 2760 |  |  |
| phchp144v1 | SZ | M | 56 | African American | 35 | No | Yes | Yes | 2849 | 2849 |  |  |
| phchp164v1 | MDD | F | 48 | Caucasian | 49 | No | Yes | Yes | 2801 | 2801 |  |  |
| phchp164v2 | MDD | F | 49 | Caucasian | 56 | No | Yes | Yes | 2681 | 2681 |  |  |
| phchp164v3 | MDD | F | 49 | Caucasian | 47 | No | Yes | Yes | 2583 | 2583 |  |  |
| phchp174v1 | MDD | M | 54 | Caucasian | 55 | No | Yes | Yes | 2775 | 2775 |  |  |
| phchp191v1 | SZA | M | 58 | African American | 49 | No | Yes | Yes | 2702 | 2702 |  |  |
| phchp191v2 | SZA | M | 58 | African American | 55 | No | Yes | Yes | 2607 | 2607 |  |  |
| phchp191v3 | SZA | M | 59 | African American | 47 | No | Yes | Yes | 2503 | 2503 |  |  |
| phchp197v1 | SZ | M | 56 | Caucasian | 64 | No | Yes | Yes | 2646 | 2646 |  |  |
| phchp197v2 | SZ | M | 57 | Caucasian | 55 | No | Yes | Yes | 2275 | 2275 |  |  |
| phchp197v3 | SZ | M | 57 | Caucasian | 55 | No | Yes | Yes | 2177 | 2177 |  |  |
| phchp197v4 | SZ | M | 58 | Caucasian | 55 | No | Yes | Yes | 2040 | 2040 |  |  |
| phchp198v1 | MDD | M | 61 | Caucasian | 46 | No | Yes | Yes | 2626 | 2626 |  |  |
| phchp198v2 | MDD | M | 61 | Caucasian | 40 | No | Yes | Yes | 2537 | 2537 |  |  |
| phchp198v4 | MDD | M | 62 | Caucasian | 40 | No | Yes | Yes | 2361 | 2361 |  |  |
| phchp203v1 | MOOD | M | 59 | African American | 47 | No | Yes | Yes | 2599 | 2599 |  |  |
| phchp203v2 | MOOD | M | 59 | African American | 41 | No | Yes | Yes | 2508 | 2508 |  |  |
| phchp206v1 | MDD | M | 59 | African American | 32 | No | Yes | Yes | 2587 | 2587 |  |  |
| phchp212v1 | MDD | M | 56 | African American | 75 | No | Yes | Yes | 2527 | 2527 |  |  |
| phchp212v2 | MDD | M | 56 | African American | 75 | No | Yes | Yes | 2447 | 2447 |  |  |
| phchp222v2 | SZ | M | 60 | Caucasian | 46 | No | Yes | Yes | 2233 | 2233 |  |  |
| phchp222v3 | SZ | M | 61 | Caucasian | 43 | No | Yes | Yes | 2144 | 2144 |  |  |
| phchp224v1 | BP | M | 59 | Caucasian | 21 | No | Yes | Yes | 2480 | 2480 |  |  |
| phchp229v1 | PTSD | M | 55 | African American | 31 | No | Yes | Yes | 2459 | 2459 |  |  |
| phchp236v1 | MDD | M | 51 | Caucasian | 47 | No | Yes | Yes | 2451 | 2451 |  |  |
| phchp236v2 | MDD | M | 51 | Caucasian | 56 | No | Yes | Yes | 2359 | 2359 |  |  |
| phchp236v3 | MDD | M | 54 | Caucasian | 48 | No | Yes | Yes | 1593 | 1593 |  |  |
| phchp243v1 | PTSD | M | 50 | African American | 48 | No | Yes | Yes | 2387 | 2387 |  |  |
| phchp243v2 | PTSD | M | 50 | African American | 41 | No | Yes | Yes | 2200 | 2200 |  |  |
| phchp243v3 | PTSD | M | 52 | African American | 48 | No | Yes | Yes | 1600 | 1600 |  |  |
| phchp243v4 | PTSD | M | 54 | African American | 55 | No | Yes | Yes | 917 | 917 |  |  |
| phchp247v1 | MDD | M | 55 | African American | 20 | No | Yes | Yes | 2445 | 2445 |  |  |
| phchp248v1 | SZ | M | 52 | African American | 20 | No | Yes | Yes | 2440 | 2440 |  |  |
| phchp248v2 | SZ | M | 52 | African American | 20 | No | Yes | Yes | 2341 | 2341 |  |  |
| phchp248v3 | SZ | M | 53 | African American | 23 | No | Yes | Yes | 2249 | 2249 |  |  |
| phchp248v5 | SZ | M | 55 | African American | 20 | No | Yes | Yes | 1218 | 1218 |  |  |
| phchp283v1 | SZ | M | 51 | Caucasian | 65 | No | Yes | Yes | 2104 | 2104 |  |  |
| phchp285v1 | BP | F | 56 | Caucasian | 47 | No | Yes | Yes | 2086 | 2086 |  |  |
| phchp285v2 | BP | F | 56 | Caucasian | 48 | No | Yes | Yes | 1948 | 1948 |  |  |
| phchp291v3 | SZ | F | 47 | Caucasian | 55 | No | Yes | Yes | 1449 | 1449 |  |  |
| phchp291v4 | SZ | F | 48 | Caucasian | 56 | No | Yes | Yes | 1312 | 1312 |  |  |
| phchp291v5 | SZ | F | 48 | Caucasian | 57 | No | Yes | Yes | 1197 | 1197 |  |  |
| phchp295v1 | SZ | M | 52 | African American | 37 | No | Yes | Yes | 2195 | 2195 |  |  |
| phchp296v3 | BP | M | 50 | Caucasian | 47 | No | Yes | Yes | 1362 | 1362 |  |  |
| phchp304v1 | MDD | M | 52 | Caucasian | 65 | No | Yes | Yes | 1932 | 1932 |  |  |
| phchp304v2 | MDD | M | 52 | Caucasian | 66 | No | Yes | Yes | 1834 | 1834 |  |  |
| phchp304v3 | MDD | M | 52 | Caucasian | 76 | No | Yes | Yes | 1676 | 1676 |  |  |
| phchp307v1 | PTSD | F | 53 | Caucasian | 90 | No | Yes | Yes | 1492 | 1492 |  |  |
| phchp314v1 | BP | M | 54 | Caucasian | 55 | No | Yes | Yes | 1808 | 1808 |  |  |
| phchp314v2 | BP | M | 54 | Caucasian | 55 | No | Yes | Yes | 1704 | 1704 |  |  |
| phchp314v3 | BP | M | 54 | Caucasian | 48 | No | Yes | Yes | 1613 | 1613 |  |  |
| phchp315v1 | MDD | M | 62 | Caucasian | 40 | No | Yes | Yes | 1778 | 1778 |  |  |
| phchp336v2 | MDD | M | 61 | African American | 39 | No | Yes | Yes | 1394 | 1394 |  |  |
| phchp336v3 | MDD | M | 62 | African American | 39 | No | Yes | Yes | 1289 | 1289 |  |  |
| phchp340v2 | MDD | F | 51 | Caucasian | 56 | No | Yes | Yes | 1491 | 1491 |  |  |
| phchp340v3 | MDD | F | 52 | Caucasian | 65 | No | Yes | Yes | 1369 | 1369 |  |  |
| phchp343v1 | MDD | M | 52 | Caucasian | 56 | No | Yes | Yes | 1557 | 1557 |  |  |
| phchp343v2 | MDD | M | 52 | Caucasian | 56 | No | Yes | Yes | 1465 | 1465 |  |  |
| phchp343v3 | MDD | M | 53 | Caucasian | 63 | No | Yes | Yes | 1299 | 1299 |  |  |
| phchp348v1 | BP | M | 52 | Caucasian | 35 | No | Yes | Yes | 1543 | 1543 |  |  |
| phchp360v1 | BP | F | 56 | Caucasian | 48 | No | Yes | Yes | 1355 | 1355 |  |  |
| phchp360v3 | BP | F | 57 | Caucasian | 55 | No | Yes | Yes | 1106 | 1106 |  |  |
| phchp365v1 | MDD | M | 59 | African American | 37 | No | Yes | Yes | 1234 | 1234 |  |  |
| phchp366v1 | SZ | M | 56 | Caucasian | 25 | No | Yes | Yes | 1183 | 1183 |  |  |
| phchp228v1 | PTSD | M | 43 | African American | 31 | No | Yes |  | 2467 | 1105 |  |  |
| phchp005v1 | SZA | M | 45 | Caucasian | 44 | No | Yes |  | 4678 | 4678 |  |  |
| phchp005v2 | SZA | M | 45 | Caucasian | 47 | No | Yes |  | 4547 | 4547 |  |  |
| phchp005v3 | SZA | M | 45 | Caucasian | 55 | No | Yes |  | 4452 | 4452 |  |  |
| phchp008v1 | SZ | M | 47 | African American | 69 | No | Yes |  | 4636 | 4636 |  |  |
| phchp014v1 | PSYCH | M | 55 | African American | 55 | No | Yes |  | 4592 | 4592 |  |  |
| phchp018v1 | SZA | F | 54 | Caucasian | 55 | No | Yes |  |  |  |  |  |
| phchp022v1 | SZ | M | 48 | Caucasian | 56 | No | Yes |  | 4524 | 4524 |  |  |
| phchp022v2 | SZ | M | 48 | Caucasian | 56 | No | Yes |  | 4421 | 4421 |  |  |
| phchp023v1 | BP | M | 52 | Caucasian | 56 | No | Yes |  |  |  |  |  |
| phchp023v2 | BP | M | 52 | Caucasian | 48 | No | Yes |  |  |  |  |  |
| phchp023v3 | BP | M | 52 | Caucasian | 56 | No | Yes |  |  |  |  |  |
| phchp024v1 | SZA | M | 49 | African American | 32 | No | Yes |  | 4512 | 4512 |  |  |
| phchp025v1 | SZ | M | 42 | Caucasian | 55 | No | Yes |  | 4510 | 4510 |  |  |
| phchp029v1 | MDD | M | 56 | Caucasian | 48 | No | Yes |  |  |  |  |  |
| phchp030v1 | BP | M | 49 | Caucasian | 47 | No | Yes |  | 4484 | 4484 |  |  |
| phchp030v3 | BP | M | 49 | Caucasian | 48 | No | Yes |  | 4256 | 4256 |  |  |
| phchp033v1 | SZA | M | 48 | Caucasian | 28 | No | Yes |  | 4454 | 4454 |  |  |
| phchp034v1 | BP | F | 51 | Asian American | 56 | No | Yes |  |  |  |  |  |
| phchp034v2 | BP | F | 52 | Asian American | 56 | No | Yes |  |  |  |  |  |
| phchp034v3 | BP | F | 52 | Asian American | 56 | No | Yes |  |  |  |  |  |
| phchp037v1 | BP | F | 52 | Caucasian | 56 | No | Yes |  |  |  |  |  |
| phchp057v1 | SZA | M | 47 | Caucasian | 55 | No | Yes |  | 4193 | 4193 |  |  |
| phchp063v1 | SZ | F | 46 | African American | 68 | No | Yes |  |  |  |  |  |
| phchp064v1 | BP | F | 59 | African American | 46 | No | Yes |  |  |  |  |  |
| phchp064v3 | BP | F | 59 | African American | 46 | No | Yes |  |  |  |  |  |
| phchp071v1 | SZA | F | 50 | African American | 38 | No | Yes |  |  |  |  |  |
| phchp076v1 | SZA | F | 41 | African American | 55 | No | Yes |  | 4008 | 4008 |  |  |
| phchp076v2 | SZA | F | 41 | African American | 47 | No | Yes |  | 3906 | 3906 |  |  |
| phchp076v3 | SZA | F | 41 | African American | 47 | No | Yes |  | 3826 | 3826 |  |  |
| phchp078v1 | BP | F | 62 | Caucasian | 30 | No | Yes |  |  |  |  |  |
| phchp080v1 | BP | M | 44 | Caucasian | 48 | No | Yes |  | 3908 | 3908 |  |  |
| phchp092v1 | BP | M | 45 | African American | 42 | No | Yes |  | 3661 | 3661 |  |  |
| phchp092v2 | BP | M | 46 | African American | 42 | No | Yes |  | 3528 | 3528 |  |  |
| phchp092v3 | BP | M | 46 | African American | 48 | No | Yes |  | 3453 | 3453 |  |  |
| phchp094v1 | BP | M | 41 | African American | 55 | No | Yes |  | 3504 | 3504 |  |  |
| phchp097v3 | SZA | F | 26 | Caucasian | 38 | No | Yes |  |  |  |  |  |
| phchp100v1 | BP | M | 28 | Caucasian | 36 | No | Yes |  | 3385 | 3385 |  |  |
| phchp102v1 | SZA | M | 56 | Caucasian | 55 | No | Yes |  |  |  |  |  |
| phchp102v2 | SZA | M | 56 | Caucasian | 46 | No | Yes |  |  |  |  |  |
| phchp102v3 | SZA | M | 56 | Caucasian | 55 | No | Yes |  |  |  |  |  |
| phchp103v1 | SZA | M | 61 | Caucasian | 20 | No | Yes |  |  |  |  |  |
| phchp113v1 | BP | M | 37 | Caucasian | 55 | No | Yes |  | 3164 | 3164 |  |  |
| phchp116v1 | SZA | M | 47 | Caucasian | 40 | No | Yes |  | 3152 | 3152 |  |  |
| phchp130v1 | MDD | F | 42 | Caucasian | 55 | No | Yes |  |  |  |  |  |
| phchp130v2 | MDD | F | 42 | Caucasian | 50 | No | Yes |  |  |  |  |  |
| phchp130v3 | MDD | F | 42 | Caucasian | 55 | No | Yes |  |  |  |  |  |
| phchp139v1 | SZ | M | 24 | Caucasian | 20 | No | Yes |  | 2971 | 2971 |  |  |
| phchp147v1 | BP | M | 38 | Caucasian | 55 | No | Yes |  | 2872 | 2872 |  |  |
| phchp147v2 | BP | M | 38 | Caucasian | 55 | No | Yes |  | 2782 | 2782 |  |  |
| phchp147v3 | BP | M | 38 | Caucasian | 55 | No | Yes |  | 2695 | 2695 |  |  |
| phchp148v1 | SZ | M | 25 | Caucasian | 24 | No | Yes |  | 2828 | 2828 |  |  |
| phchp152v1 | BP | M | 45 | Caucasian | 20 | No | Yes |  | 2921 | 2921 |  |  |
| phchp156v1 | BP | F | 35 | Caucasian | 39 | No | Yes |  | 2809 | 2809 |  |  |
| phchp158v1 | BP | M | 23 | African American | 36 | No | Yes |  | 2828 | 2828 |  |  |
| phchp167v1 | MDD | M | 49 | Caucasian | 28 | No | Yes |  | 2795 | 2795 |  |  |
| phchp169v1 | SZA | M | 50 | African American | 20 | No | Yes |  | 2793 | 2793 |  |  |
| phchp171v1 | BP | M | 36 | Caucasian | 28 | No | Yes |  | 2755 | 2755 |  |  |
| phchp171v2 | BP | M | 36 | Caucasian | 31 | No | Yes |  | 2629 | 2629 |  |  |
| phchp173v1 | MDD | M | 48 | Caucasian | 56 | No | Yes |  | 2605 | 2605 |  |  |
| phchp173v2 | MDD | M | 49 | Caucasian | 56 | No | Yes |  | 2516 | 2516 |  |  |
| phchp173v3 | MDD | M | 49 | Caucasian | 56 | No | Yes |  | 2425 | 2425 |  |  |
| phchp175v1 | SZA | M | 42 | Caucasian | 20 | No | Yes |  | 2774 | 2774 |  |  |
| phchp177v1 | SZ | F | 39 | Caucasian | 43 | No | Yes |  | 2762 | 2762 |  |  |
| phchp177v2 | SZ | F | 39 | Caucasian | 48 | No | Yes |  | 2653 | 2653 |  |  |
| phchp178v1 | BP | M | 49 | Caucasian | 56 | No | Yes |  | 2744 | 2744 |  |  |
| phchp180v1 | BP | F | 47 | Caucasian | 46 | No | Yes |  |  |  |  |  |
| phchp180v2 | BP | F | 47 | Caucasian | 47 | No | Yes |  |  |  |  |  |
| phchp180v3 | BP | F | 47 | Caucasian | 48 | No | Yes |  |  |  |  |  |
| phchp182v1 | MDD | M | 39 | Caucasian | 55 | No | Yes |  | 2732 | 2732 |  |  |
| phchp182v2 | MDD | M | 39 | Caucasian | 55 | No | Yes |  | 2635 | 2635 |  |  |
| phchp182v3 | MDD | M | 40 | Caucasian | 62 | No | Yes |  | 2544 | 2544 |  |  |
| phchp207v1 | SZ | M | 48 | African American | 20 | No | Yes |  | 2565 | 2565 |  |  |
| phchp223v1 | SZA | F | 60 | Caucasian | 55 | No | Yes |  |  |  |  |  |
| phchp223v2 | SZA | F | 60 | Caucasian | 49 | No | Yes |  |  |  |  |  |
| phchp223v3 | SZA | F | 61 | Caucasian | 55 | No | Yes |  |  |  |  |  |
| phchp225v1 | PSYCH | M | 58 | African American | 40 | No | Yes |  |  |  |  |  |
| phchp231v1 | MDD | M | 55 | Caucasian | 57 | No | Yes |  |  |  |  |  |
| phchp232v1 | SZA | F | 38 | Caucasian | 55 | No | Yes |  |  |  |  |  |
| phchp232v2 | SZA | F | 38 | Caucasian | 55 | No | Yes |  |  |  |  |  |
| phchp232v3 | SZA | F | 38 | Caucasian | 55 | No | Yes |  |  |  |  |  |
| phchp234v1 | BP | M | 44 | Caucasian | 55 | No | Yes |  | 2452 | 2452 |  |  |
| phchp234v2 | BP | M | 45 | Caucasian | 49 | No | Yes |  | 2242 | 2242 |  |  |
| phchp234v3 | BP | M | 45 | Caucasian | 55 | No | Yes |  | 2151 | 2151 |  |  |
| phchp237v1 | PTSD | M | 39 | Caucasian | 62 | No | Yes |  | 2454 | 2454 |  |  |
| phchp237v2 | PTSD | M | 39 | Caucasian | 55 | No | Yes |  | 2338 | 2338 |  |  |
| phchp239v1 | SZA | F | 54 | African American | 23 | No | Yes |  |  |  |  |  |
| phchp239v2 | SZA | F | 54 | African American | 21 | No | Yes |  |  |  |  |  |
| phchp239v3 | SZA | F | 54 | African American | 25 | No | Yes |  |  |  |  |  |
| phchp241v1 | BP | M | 52 | Caucasian | 20 | No | Yes |  |  |  |  |  |
| phchp265v1 | PTSD | M | 43 | Caucasian | 39 | No | Yes |  | 2118 | 2118 |  |  |
| phchp266v1 | MOOD | M | 41 | Caucasian | 55 | No | Yes |  | 2411 | 2411 |  |  |
| phchp266v2 | MOOD | M | 42 | Caucasian | 55 | No | Yes |  | 2310 | 2310 |  |  |
| phchp266v3 | MOOD | M | 42 | Caucasian | 50 | No | Yes |  | 2214 | 2214 |  |  |
| phchp286v1 | BP | M | 54 | Caucasian | 47 | No | Yes |  |  |  |  |  |
| phchp286v2 | BP | M | 54 | Caucasian | 55 | No | Yes |  |  |  |  |  |
| phchp286v3 | BP | M | 55 | Caucasian | 55 | No | Yes |  |  |  |  |  |
| phchp291v1 | SZ | F | 45 | Caucasian | 48 | No | Yes |  | 2207 | 2207 |  |  |
| phchp291v2 | SZ | F | 46 | Caucasian | 55 | No | Yes |  | 2067 | 2067 |  |  |
| phchp293v1 | BP | M | 43 | Caucasian | 55 | No | Yes |  | 2184 | 2184 |  |  |
| phchp293v2 | BP | M | 44 | Caucasian | 55 | No | Yes |  | 2082 | 2082 |  |  |
| phchp296v1 | BP | M | 48 | Caucasian | 41 | No | Yes |  | 2104 | 2104 |  |  |
| phchp296v2 | BP | M | 49 | Caucasian | 47 | No | Yes |  | 1836 | 1836 |  |  |
| phchp323v3 | PTSD | M | 33 | Caucasian | 35 | No | Yes |  | 1547 | 1547 |  |  |
| phchp324v1 | MDD | M | 33 | African American | 36 | No | Yes |  | 1767 | 1767 |  |  |
| phchp327v1 | MDD | M | 42 | Caucasian | 33 | No | Yes |  | 1754 | 1754 |  |  |
| phchp330v1 | BP | F | 45 | Caucasian | 22 | No | Yes |  | 1694 | 1694 |  |  |
| phchp331v1 | BP | M | 53 | Caucasian | 25 | No | Yes |  |  |  |  |  |
| phchp341v2 | MDD | M | 45 | Caucasian | 55 | No | Yes |  | 1479 | 1479 |  |  |
| phchp341v3 | MDD | M | 45 | Caucasian | 56 | No | Yes |  | 1379 | 1379 |  |  |
| phchp341v4 | MDD | M | 46 | Caucasian | 48 | No | Yes |  | 1113 | 1113 |  |  |
| phchp341v5 | MDD | M | 46 | Caucasian | 56 | No | Yes |  | 980 | 980 |  |  |
| phchp351v1 | MDD | M | 44 | Caucasian | 50 | No | Yes |  | 1498 | 1498 |  |  |
| phchp351v2 | MDD | M | 44 | Caucasian | 44 | No | Yes |  | 1400 | 1400 |  |  |
| phchp353v1 | PTSD | F | 45 | Caucasian | 55 | No | Yes |  | 1474 | 1474 |  |  |
| phchp356v1 | BP | M | 40 | Caucasian | 38 | No | Yes |  | 1446 | 1446 |  |  |
| phchp357v1 | BP | M | 45 | Caucasian | 55 | No | Yes |  | 1445 | 1445 |  |  |
| phchp357v2 | BP | M | 45 | Caucasian | 56 | No | Yes |  | 1351 | 1351 |  |  |
| phchp357v3 | BP | M | 45 | Caucasian | 49 | No | Yes |  | 1212 | 1212 |  |  |
| phchp357v4 | BP | M | 45 | Caucasian | 55 | No | Yes |  | 1120 | 1120 |  |  |
| phchp357v5 | BP | M | 46 | Caucasian | 55 | No | Yes |  | 966 | 966 |  |  |
| phchp361v1 | PTSD | F | 59 | African American | 38 | No | Yes |  | 1361 | 1361 |  |  |
| phchp361v2 | PTSD | F | 60 | African American | 38 | No | Yes |  | 1236 | 1236 |  |  |
| phchp361v3 | PTSD | F | 60 | African American | 43 | No | Yes |  | 1113 | 1113 |  |  |
| phchp364v1 | PTSD | F | 40 | Caucasian | 55 | No | Yes |  | 1232 | 1232 |  |  |
| phchp368v1 | MDD | F | 43 | Caucasian | 39 | No | Yes |  | 1155 | 1155 |  |  |
| phchp368v2 | MDD | F | 44 | Caucasian | 42 | No | Yes |  | 812 | 812 |  |  |
