# Supplementary material for: Blood biomarkers for memory: toward early detection of risk for Alzheimer disease, pharmacogenomics, and repurposed drugs
Source: Mol Psychiatry. 2019 Dec 2;25(8):1651–72. doi: 10.1038/s41380-019-0602-2 (PMC7387316; doi:10.1038/s41380-019-0602-2)
Supplement: Supplementary file 2 — Supplementary Information -Figures S1-S3 and Tables S2-S4 [file 41380_2019_602_MOESM2_ESM.docx]

**Supplementary Information:**

# Figure S1. Hopkins Verbal Learning Test-Delayed Recognition (HVLT-DR). We used the Retention measure from it.


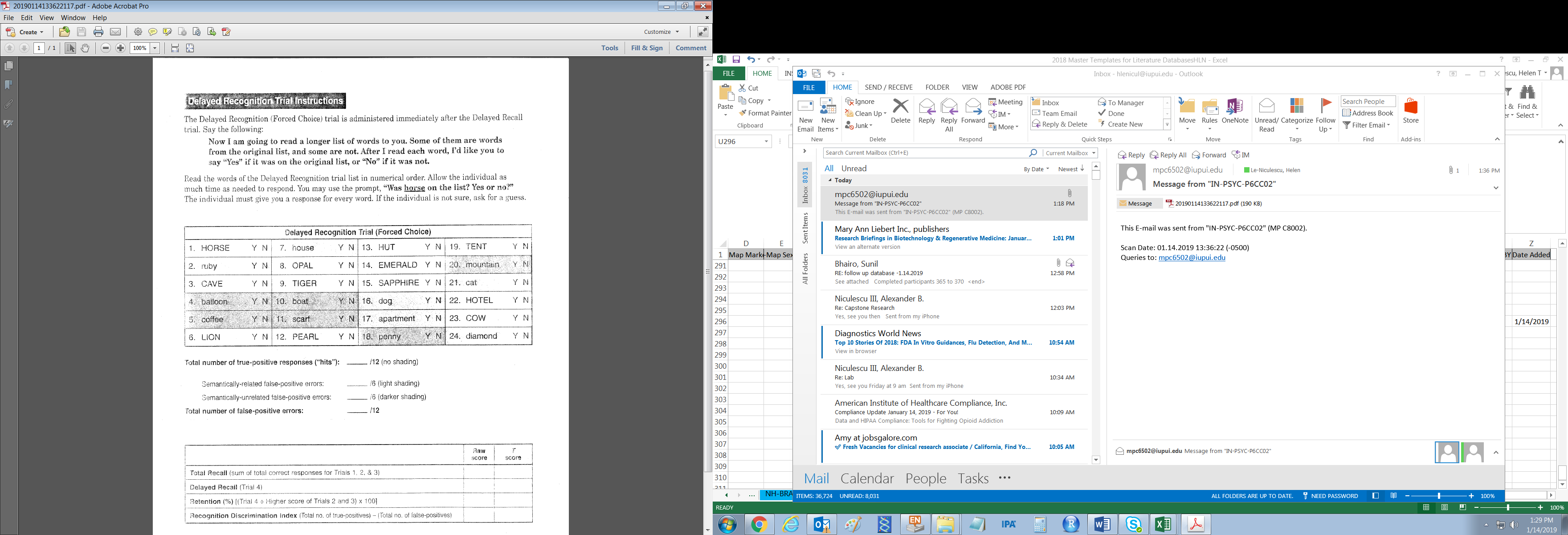


**Figure S2 RHEB as a Possible Personalized Biomarker Predictor for Risk of Future AD in Males with Schizophrenia.** Subject Phchp098 is a male with schizophrenia (SZ) tested in our lab in 2009. He was first diagnosed with paranoid schizophrenia in 1977. In 2016, he was also diagnosed by neuropsychological testing with ADRD and impaired decision-making capacity. At that time, he was 66 years old. Subject is the only one so far with an ADRD diagnosis in the independent replication follow-up cohort. We tested RHEB, the best predictive biomarker for males with SZ (Figure 2B). RHEB levels were Z-scored by gender and diagnosis. Subject Phchp098 had the highest levels of RHEB in our lab testing visit from all the subjects with future neuropsychological testing (A.), and in fact the highest level of RHEB from all the 111 subjects in that cohort (B).


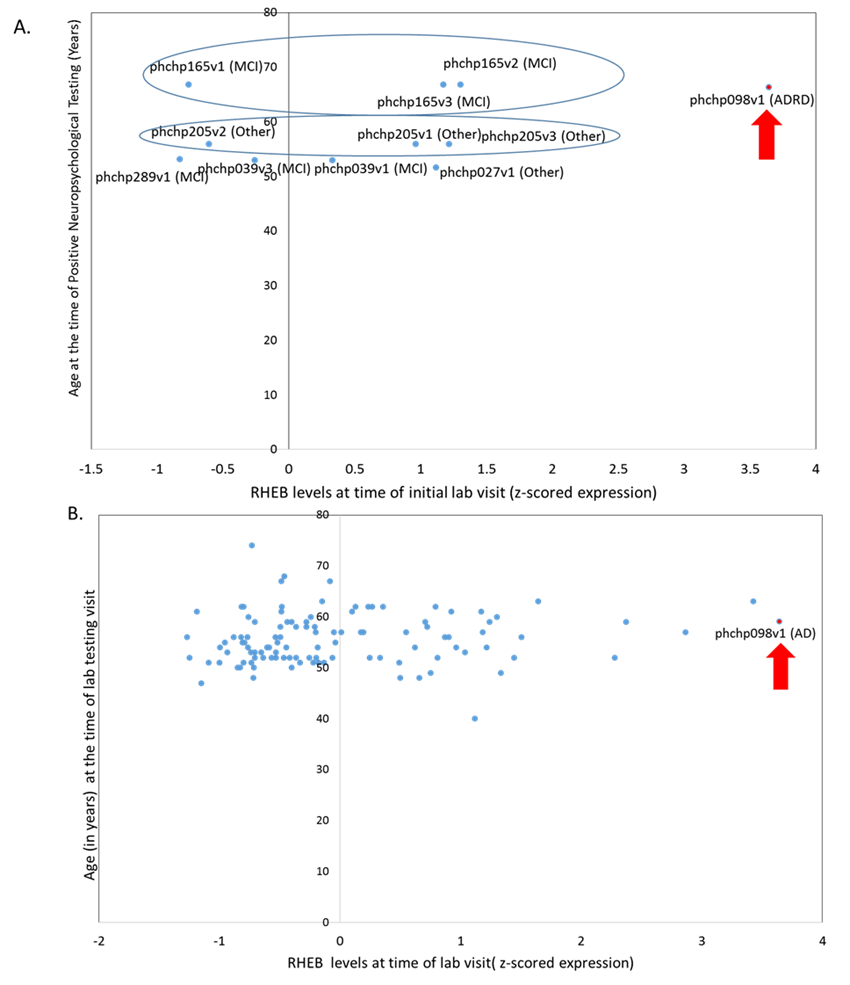


**Figure S3 Interaction Networks.** For Top Candidate Biomarkers (n=112 top genes, 138 probesets). STRING analysis. The STRING gene interaction analysis revealed at least 3 networks (boxed with red, green and blue). The links between nodes depict various types of evidence of interaction (see (<https://string-db.org>).


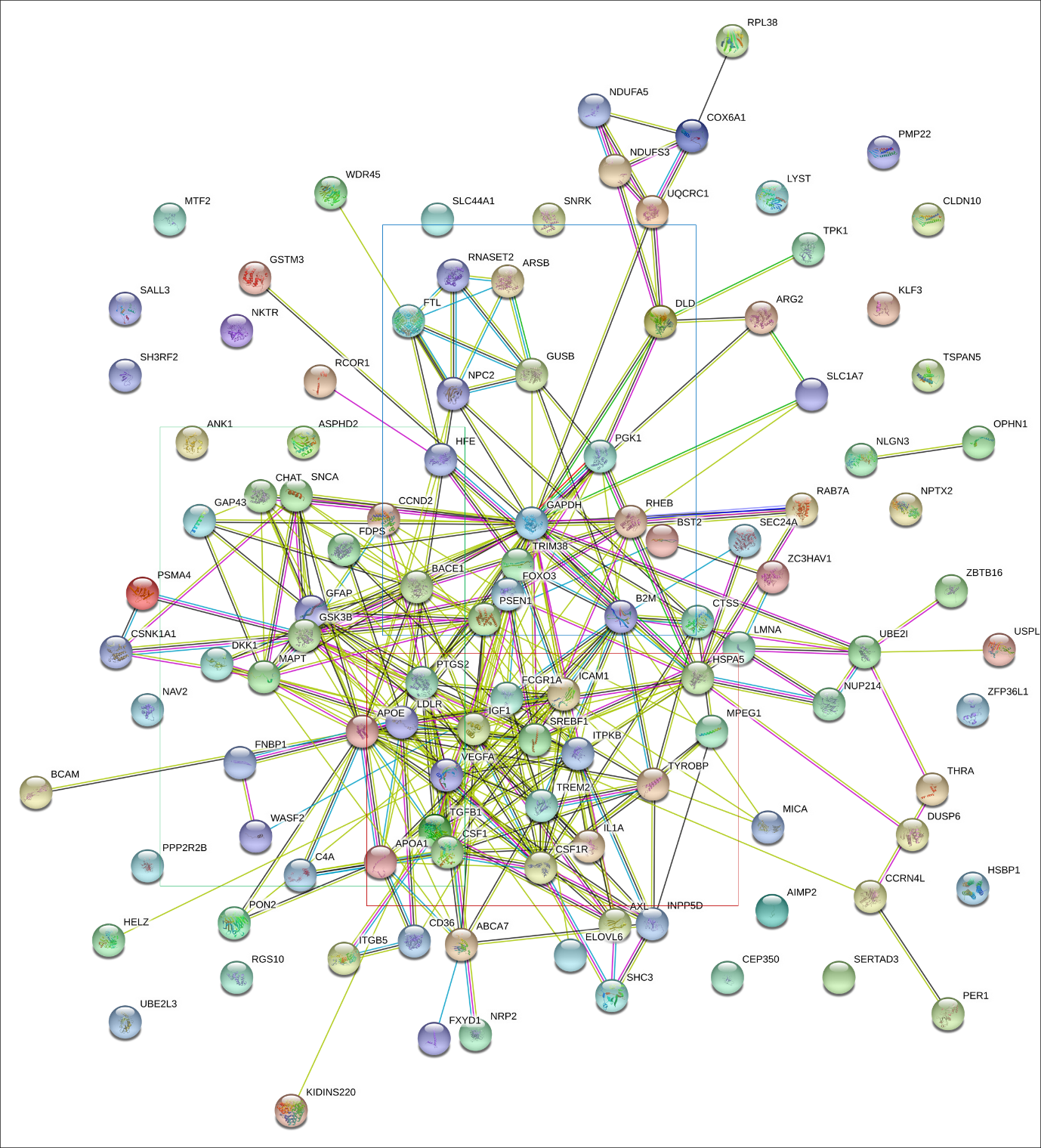


**Table S2. Top Biomarkers (from Table 3). Evidence for involvement in Alzheimer’s Disease.** Assoc- Genetic Association.

| **Gene symbol/**  **Gene name** | **Probesets** | **Step 1  Discovery in Blood  (Direction of Change tracking Memory Increase) Method/ Score/ %  Up to 6 pts** | **Prior human genetic evidence for AD**  **Score**  **2pts** | **Prior human Brain evidence for AD**  **Score**  **4 pts** | **Prior human peripheral evidence for AD**  **Score**  **2 pts** | **Prior Non-human genetic evidence for AD**  **Score**  **1pt** | **Prior Non-human Brain evidence for AD**  **Score**  **2pts.** | **Prior Non-human peripheral evidence for AD**  **Score**  **1pt** | **Step 2  External CFG Evidence For Involvement in AD   Score  Up to 12pt** |
| --- | --- | --- | --- | --- | --- | --- | --- | --- | --- |
| **BACE1**  **Beta-Secretase 1** | 222463_s_at  224335_s_at | (I) DE/2 44.8%  (I) DE/2 43.1% | Assoc [^1^](#_ENREF_1) [^2^](#_ENREF_2) [^3^](#_ENREF_3) [^4^](#_ENREF_4) | (I)  medial temporal and superior parietal gyri  AD[^5^](#_ENREF_5) | Elevated activity CSF  AD  [^6^](#_ENREF_6) | AD[^7^](#_ENREF_7) | (I) (APP23/TNFRII(-/-)) Mice Brain AD [^8^](#_ENREF_8) | activity [^9^](#_ENREF_9) | 12 |
| **APOE apolipoprotein E** | 212884_x_at | (D) AP/2 34.1% | Assoc [^10^](#_ENREF_10) [^11^](#_ENREF_11) [^12^](#_ENREF_12) [^13^](#_ENREF_13) [^14^](#_ENREF_14) [^15^](#_ENREF_15)  Linkage  [^16^](#_ENREF_16) [^17^](#_ENREF_17) [^18^](#_ENREF_18) [^19^](#_ENREF_19) | (D) HIP AD [^14^](#_ENREF_14) | (D)  Plasma  [^20^](#_ENREF_20)  (I)  Plasma  [^21^](#_ENREF_21) | APOE  knock-in mice [^22^](#_ENREF_22) | (I) Cortex AD [^23^](#_ENREF_23) |  | 11 |
| **TREM2 triggering receptor expressed on myeloid cells 2** | 219725_at | (I) DE/2 37.6% | Assoc  [^24^](#_ENREF_24) [^25^](#_ENREF_25) [^26^](#_ENREF_26) [^25^](#_ENREF_25) [^27^](#_ENREF_27)  Linkage  [^18^](#_ENREF_18) [^16^](#_ENREF_16) | (I) Temporal Cortical AD  [^28^](#_ENREF_28) | (I)  CSF  AD [^29^](#_ENREF_29)  (I)  CSF  AD [^30^](#_ENREF_30)  (I)  CSF  AD [^31^](#_ENREF_31)  (I)  PBMC  AD[^32^](#_ENREF_32) [^33^](#_ENREF_33) | Mouse model  [^34^](#_ENREF_34) | (I) Cortex AD [^23^](#_ENREF_23)    (I) Cerebrum  AD [^35^](#_ENREF_35)^,^ [^36^](#_ENREF_36)  (I) Cortexl  AD [^35^](#_ENREF_35)^,^ [^36^](#_ENREF_36) |  | 11 |
| **GSK3B glycogen synthase kinase 3 beta** | 209945_s_at  240562_at  242336_at | (D) DE/4 50.3%  (I) DE/2 39.2%  (D) AP/2 34.1% | Assoc  [^37^](#_ENREF_37) [^38^](#_ENREF_38) [^39^](#_ENREF_39)  Linkage  [^18^](#_ENREF_18) | (I) HIP AD [^40^](#_ENREF_40) | (I)  White blood cells AD [^41^](#_ENREF_41) | Mouse model[^42^](#_ENREF_42) | (D) HIP APP/PS1 Double-Transgenic Mice Astaxanthin-DHA [^43^](#_ENREF_43) |  | 10 |
| **MAPT microtubule associated protein tau** | 203928_x_at  203930_s_at  233117_at | (I) DE/4 57.5%  (I) DE/2 33.7%  (I) DE/2 44.2% | Assoc  [^44^](#_ENREF_44) [^45^](#_ENREF_45) [^46^](#_ENREF_46)  Linkage  [^18^](#_ENREF_18) | (I) Cortical AD [^47^](#_ENREF_47) | (I) Plasma  [^48^](#_ENREF_48)  (I) CSF [^49^](#_ENREF_49)  (I)  CSF AD [^50^](#_ENREF_50)  (I) CSF AD [^51^](#_ENREF_51) [^52^](#_ENREF_52) [^47^](#_ENREF_47) [^53^](#_ENREF_53) | Mouse model [^54^](#_ENREF_54) | (D) HIP APP/PS1 Double-Transgenic Mice Astaxanthin-DHA) [^43^](#_ENREF_43) |  | 10 |
| **PTGS2 prostaglandin-endoperoxide synthase 2 (prostaglandin G/H synthase and cyclooxygenase)** | 1554997_a_at | (D) DE/4 76% | Assoc  [^55^](#_ENREF_55) | (I) Hippocampal Cornu ammonis 1 (CA1) AD [^56^](#_ENREF_56)  (I) HIP AD [^57^](#_ENREF_57) | (D) Blood mononuclear cell (BMC) AD [^58^](#_ENREF_58) |  | (D) Cortex AD [^23^](#_ENREF_23) |  | 10 |
| GFAP glial fibrillary acidic protein | 203540_at | (I) DE/2 34.3% | Linkage  [^18^](#_ENREF_18) | (I)  Temporal Cortex AD [^23^](#_ENREF_23)  (I)  HIP AD[^59^](#_ENREF_59)  (I)  Frontal cortex & HIP AD [^60^](#_ENREF_60)  (D)  HIP AD[^40^](#_ENREF_40)  (D)  HIP AD[^61^](#_ENREF_61) | (I)  CSF  AD [^62^](#_ENREF_62)  (I) Serum AD [^63^](#_ENREF_63) |  | (I) Cortex AD [^23^](#_ENREF_23)  (I) Astrocytes AD [^23^](#_ENREF_23) |  | 9 |
| **PSEN1 presenilin 1** | 203460_s_at | (D) DE/4 54.5% | Assoc [^64^](#_ENREF_64),[^65^](#_ENREF_65),[^66^](#_ENREF_66), [^67^](#_ENREF_67) | (I) Temporal Cortex AD [^23^](#_ENREF_23) |  | Mouse model  [^68^](#_ENREF_68) [^69^](#_ENREF_69) | (I)  Mouse model[^70^](#_ENREF_70) |  | 9 |
| **TGFB1 transforming growth factor beta 1** | 203084_at | (D) AP/4 54.5% | Assoc [^71^](#_ENREF_71)^,^ [^72^](#_ENREF_72)  Linkage[^18^](#_ENREF_18) | (I) HIP AD [^40^](#_ENREF_40) |  | Mouse model  [^73^](#_ENREF_73) | (I)  Cortex AD [^23^](#_ENREF_23) |  | 9 |
| **CTSS cathepsin S** | 232617_at | (D) DE/4 56.9% | Assoc  [^74^](#_ENREF_74)  Linkage  [^18^](#_ENREF_18) | (I) Temporal Cortex AD [^23^](#_ENREF_23)  (I) HIP AD [^40^](#_ENREF_40) |  |  | (I) Cortex AD [^23^](#_ENREF_23) |  | 8 |
| **GSTM3 glutathione S-transferase mu 3 (brain)** | 235867_at | (D) DE/4 52.1% | Assoc  [^75^](#_ENREF_75) [^76^](#_ENREF_76)  Linkage  [^18^](#_ENREF_18) [^77^](#_ENREF_77) | (I) Temporal Cortex AD [^23^](#_ENREF_23)  (D) HIP AD [^40^](#_ENREF_40) | (D) Blood mononuclear cell (BMC) AD [^58^](#_ENREF_58) |  |  |  | 8 |
| **GUSB glucuronidase, beta** | 202605_at | (D) DE/4 55.7% |  | (I) HIP AD [^40^](#_ENREF_40) | (D) Blood mononuclear cell AD[^58^](#_ENREF_58) |  | (I) Cortex AD [^23^](#_ENREF_23) |  | 8 |
| **IGF1 insulin-like growth factor 1 (somatomedin C)** | 209542_x_at | (I) DE/4 54.1% | Assoc [^78^](#_ENREF_78) | (D) HIP AD [^40^](#_ENREF_40) |  |  | (I) Cortex AD [^23^](#_ENREF_23) |  | 8 |
| **NPC2 Niemann-Pick disease, type C2** | 200701_at | (D) DE/6 80.8% | Assoc  [^79^](#_ENREF_79) | (I) Temporal Cortex AD [^23^](#_ENREF_23) |  |  | (I) Cortex AD [^23^](#_ENREF_23) |  | 8 |
| **NPTX2 neuronal pentraxin II** | 213479_at | (I) DE/4 52.5% |  | (D) Frontal Cortex ,Temporal Cortex AD [^23^](#_ENREF_23) | CSF  AD [^80^](#_ENREF_80) |  | (D) Cortex AD [^23^](#_ENREF_23) |  | 8 |
| **THRA thyroid hormone receptor, alpha** | 214883_at | (I) DE/4 61.3% | Assoc [^81^](#_ENREF_81)  Linkage[^18^](#_ENREF_18) | (I) HIP AD [^40^](#_ENREF_40) | (I) Blood mononuclear cell (BMC) AD (Males) [^58^](#_ENREF_58) |  |  |  | 8 |
| **VEGFA** vascular endothelial growth factor A | 211527_x_at | (I) DE/2 45.3% | Assoc [^82^](#_ENREF_82) [^83^](#_ENREF_83) [^84^](#_ENREF_84)  Linkage[^18^](#_ENREF_18) | (D) Temporal Cortex AD [^23^](#_ENREF_23)  (D) HIP AD [^40^](#_ENREF_40) |  |  | (D) Cortex AD [^23^](#_ENREF_23) |  | 8 |
| GAP43  growth associated protein 43 | 204471_at | (I) DE/4 50.8% | Linkage |  | (I) CSF AD [^85^](#_ENREF_85) |  |  |  | 7 |
| FCGR1A  Fc fragment of IgG, high affinity Ia, receptor (CD64) | 216951_at | (I) DE/4 64.6% | Linkage | (D) Hippocampus Dementia [^40^](#_ENREF_40) |  |  | (I) Cortex AD [^23^](#_ENREF_23) |  | 7 |
| **RAB7A** RAB7A, member RAS oncogene family | 227602_at | (I) AP/2 43.8% (I) DE/4 69.6% | Linkage[^18^](#_ENREF_18) | (D)  HIP AD [^40^](#_ENREF_40) | (D) Blood mononuclear cell (BMC) AD [^58^](#_ENREF_58) |  |  |  | 7 |
| KIDINS220  kinase D-interacting substrate 220kDa | 214932_at | (I) DE/4 51.9% |  | (D)  Hippocampus  Dementia  [^40^](#_ENREF_40) |  |  | (D) Cortex [^23^](#_ENREF_23) |  | 6 |
| **ARSB arylsulfatase B** | 1554030_at | (I) DE/6 91.7% | Assoc [^86^](#_ENREF_86) | (D)EC ,HIP,PCG ,SFG  MCI [^87^](#_ENREF_87) |  |  |  |  | 6 |
| **CD36** CD36 molecule (thrombospondin receptor) | 242197_x_at | (D) DE/4 56.9% | Assoc [^88^](#_ENREF_88) | (D) PFC AD [^89^](#_ENREF_89) |  |  |  |  | 6 |
| **CEP350** centrosomal protein 350kDa | 204373_s_at | (D) DE/4 67.1% |  | (I) HIP AD [^40^](#_ENREF_40) |  |  | (D) Cortex AD [^23^](#_ENREF_23) |  | 6 |
| **NRP2**  neuropilin 2 | 222877_at | (I) DE/4 61.3% |  | (I) HIP AD [^40^](#_ENREF_40) | (D) Blood mononuclear cell (BMC) AD [^58^](#_ENREF_58) |  |  |  | 6 |
| **ITPKB** inositol-trisphosphate 3-kinase B | 1554306_at  232526_at | (D) AP/4 61.1%  (D) DE/4 55.7%  (I) DE/4 51.9% |  | (I) Temporal Cortex AD [^23^](#_ENREF_23)  (I) Frontal cortex, (BA8, 9) AD [^90^](#_ENREF_90)  (I) HIP AD [^40^](#_ENREF_40) |  |  | (I) Cortex AD [^23^](#_ENREF_23) |  | 6 |
| **NOCT** nocturnin | 220671_at | (D) AP/4 69.5% | Assoc [^91^](#_ENREF_91) | (I) HIP AD [^40^](#_ENREF_40) |  |  |  |  | 6 |
| **PER1** period circadian clock 1 | 242832_at | (I) DE/4 61.3% |  | (I) HIP AD [^40^](#_ENREF_40) |  |  | (D) Cortex AD [^23^](#_ENREF_23) |  | 6 |
| **PPP2R2B** protein phosphatase 2, regulatory subunit B, beta | 205643_s_at | (I) DE/4 63.5% | Assoc [^92^](#_ENREF_92) | (D) HIP AD [^40^](#_ENREF_40)  (D)  HIP  AD [^59^](#_ENREF_59) |  |  |  |  | 6 |
| **RGS10** regulator of G-protein signaling 10 | 214000_s_at | (I) DE/4 63.5% |  | (I) Temporal Cortex AD [^23^](#_ENREF_23) |  |  | (I) Cortex AD [^23^](#_ENREF_23) |  | 6 |
| **UBE2I ubiquitin conjugating enzyme E2I** | 233360_at | (D) DE/6 86.8% | Assoc [^93^](#_ENREF_93) | (D)  HIP MCI [^87^](#_ENREF_87) |  |  |  |  | 6 |
| **SERTAD3** SERTA domain containing 3 | 219382_at | (D) DE/6 81.4% | Linkage[^18^](#_ENREF_18) | (I) HIP AD [^40^](#_ENREF_40) |  |  |  |  | 5 |
| **FOXO3** forkhead box O3 | 231548_at | (I) DE/6 82.3%  (I) AP/2 38.9% |  | (I) HIP AD [^40^](#_ENREF_40) |  |  |  |  | 4 |
| **NKTR** natural killer cell triggering receptor | 1570342_at | (D) AP/6 85% |  | (I) HIP AD [^40^](#_ENREF_40) |  |  |  |  | 4 |
| **RHEB** Ras homolog enriched in brain | 243008_at | (D) AP/6 84.4% (D) DE/4 64.1% |  | (D) HIP AD [^40^](#_ENREF_40) |  |  |  |  | 4 |
| **UBE2L3** ubiquitin conjugating enzyme E2L 3 | 200682_s_at | (D) DE/6 91% |  | (I)  EC entorhinal cortex  MCI  [^87^](#_ENREF_87)  (I) HIP AD [^40^](#_ENREF_40) |  |  |  |  | 4 |

**Table S3 Top Biomarkers (from Table 3). Evidence for Involvement in Other Psychiatric and Related Disorders.**  In opposite direction to high memory. ASD- Autism Spectrum Disorder; TBI- traumatic brain injury; MDD- depression; BP-bipolar; SZ-schizophrenia; PTSD- post-traumatic stress disorder.

| **Genesymbol/**  **Gene name** | **Probesets** | **Step 1  Discovery in Blood  (Direction of Change tracking Memory Increase) Method/ Score/ %  Up to 6pts** | **Step 2  External CFG Evidence For Involvement in AD   Score  Up to 12pt** | **Human genetic evidence for Other Dx**  **2pts** | **Human Brain evidence for Other Dx**  **4 pts** | **Human peripheral evidence for Other Dx**  **2pts** | **Non-human genetic evidence for Other Dx**  **1pt** | **Non-human Brain n evidence for Other Dx**  **2pts.** | **Non-human peripheral evidence for Other Dx**  **1pt** | **External CFG Evidence for**  **Other Dx**  **Up to 12 pts.** |
| --- | --- | --- | --- | --- | --- | --- | --- | --- | --- | --- |
| **IGF1 insulin-like growth factor 1 (somatomedin C)** | 209542_x_at | (I) DE/4 54.1% | 8 | **Aging** /  **Longevity**  [^94^](#_ENREF_94)  **BP**  [^95^](#_ENREF_95) | (D)  PFC  **Aging**  [^96^](#_ENREF_96)  (D)  motor cortex  **Alcohol**  [^97^](#_ENREF_97)  (D)  DLPFC  **SZ**  [^98^](#_ENREF_98)  (D) Female  BA11  **Depression**  [^99^](#_ENREF_99) | (D)  **Aging**  [^100^](#_ENREF_100)  Differential Methylation  Blood  **Anxiety**  [^101^](#_ENREF_101) | **Depression**  [^102^](#_ENREF_102)  **Aggression**  [^103^](#_ENREF_103) | (D)  septal region  **PTSD**  [^104^](#_ENREF_104) | (D) Plasma **Alcohol**  [^105^](#_ENREF_105) | 12 |
| **APOE apolipoprotein E** | 212884_x_at | (D) AP/2 34.1% | 11 | **TBI**  [^106^](#_ENREF_106)  **Suicide**  [^107^](#_ENREF_107),[^108^](#_ENREF_108)    **PTSD**  [^106^](#_ENREF_106),[^109^](#_ENREF_109)  **Pain**  [^103^](#_ENREF_103)    **Pain**  **MSK**  [^110^](#_ENREF_110)    **Aging** /**Longevity**  [^111^](#_ENREF_111),[^112^](#_ENREF_112),  [^113^](#_ENREF_113)  **MDD**  Depression  [^114^](#_ENREF_114),[^115^](#_ENREF_115)  **Brain arousal**  [^116^](#_ENREF_116)    **Aggression**  [^117^](#_ENREF_117) | (I)  Frontal and temporal cortex  **ASD**  [^118^](#_ENREF_118)  (I)  cerebral cortex  **ASD,SZ**  [^119^](#_ENREF_119)  (I)  OFC  **BP**  [^120^](#_ENREF_120)  (I)  PFC  **BP**  [^121^](#_ENREF_121)  (I)  DLPFC (BA9)  **SZ**  [^122^](#_ENREF_122)  (I)  frontal  **SZ**  [^123^](#_ENREF_123) | (I)  Blood  **Suicide**  [^124^](#_ENREF_124),  (Female Suicide)  [^125^](#_ENREF_125)  (I)  buccal  **Stress**  **PTSD**  [^106^](#_ENREF_106)  (I)  plasma  **Stress**  **PTSD**  [^126^](#_ENREF_126) |  | (I)  NAC  **Alcohol**  [^127^](#_ENREF_127)    (I)  Hippocamus  **Alcohol**  [^128^](#_ENREF_128)  (I)  Hippocampus  **Aging**  [^129^](#_ENREF_129)  (I)  cortex  **Anxiety**  [^130^](#_ENREF_130)    (I)  PAG  Neuropathic **Pain**  [^131^](#_ENREF_131)    (I)  DRG  **Pain**  [^132^](#_ENREF_132)  (I)  Hypothalamus  **Stress**  [^133^](#_ENREF_133) | Altered Serum **Depression**  [^134^](#_ENREF_134) | 11 |
| **BACE1**  **Beta-Secretase 1** | 222463_s_at  224335_s_at | (I) DE/6 44.8%  (I) DE/6 43.1% | 8 | **Suicide** [^135^](#_ENREF_135) | (D)  BA24  **MDD,**  **Suicide**  [^136^](#_ENREF_136) | (D)  Blood  **Suicide**  [^124^](#_ENREF_124)  (I) Blood **Stress**[^137^](#_ENREF_137) | **SZ** [^138^](#_ENREF_138) | (D) NAC, PFC  Female  **Stress**  [^99^](#_ENREF_99) |  | 11 |
| KIDINS220  kinase D-interacting substrate 220kDa | 214932_at | (I) DE/4 51.9% | 6 | **Psychosis**  [^139^](#_ENREF_139)  **Pain**  **MSK**  [^140^](#_ENREF_140) | (D)  Hippocampus  **Alcohol**  [^141^](#_ENREF_141)  (D)  temporal cortex  **Alcohol**  [^142^](#_ENREF_142)  (D)  AMY and cingulate cortex  **MDD**  [^143^](#_ENREF_143) | (D)  Blood  **Suicide**  [^144^](#_ENREF_144),[^125^](#_ENREF_125),  [^124^](#_ENREF_124)  (D)  Peripheral blood monocytes  **Chronic Stress**  [^145^](#_ENREF_145)  (D) Blood **Pain** {Niculescu, 2019 #20378} | **Alcohol** [^146^](#_ENREF_146) | (D) Male  PFC  **Stress**  [^99^](#_ENREF_99) |  | 11 |
| **PPP2R2B** protein phosphatase 2, regulatory subunit B, beta | 205643_s_at | (I) DE/4 63.5% | 6 | **SZ**  [^147^](#_ENREF_147)  **Longevity**  [^111^](#_ENREF_111)  **Alcohol**  [^148^](#_ENREF_148)  **ADHD**  [^149^](#_ENREF_149) | (D)  Frontal Cortex, cerebellum  **Aging**  [^150^](#_ENREF_150)  (D)  cerebral cortex  **ASD**  [^119^](#_ENREF_119)  (D)  Forebrain neural progenitor cells  **SZ**  [^151^](#_ENREF_151)  (D)  PFC  **SZ**  [^147^](#_ENREF_147)  (D)  cerebral cortex  **SZ**  [^119^](#_ENREF_119) | (D)  Fibroblast  **SZ**  [^152^](#_ENREF_152)  (D)  Blood  **Suicide**  [^124^](#_ENREF_124)  (D)  Blood  **Circadian abnormalities**  [^153^](#_ENREF_153) |  | (D) NAC  **Alcohol** [^128^](#_ENREF_128) | (D) Spleen **PTSD** [^154^](#_ENREF_154) | 11 |
| **PTGS2 prostaglandin-endoperoxide synthase 2** | 1554997_a_at | (D) DE/4 76% | 10 | **Pain**  [^103^](#_ENREF_103)  **Substances/Addictions**  [^155^](#_ENREF_155)      **MDD**  [^156^](#_ENREF_156)    **Depression**  [^157^](#_ENREF_157)  **Chronic Fatigue Syndrome**  [^158^](#_ENREF_158)      Cancer **Pain**  [^159^](#_ENREF_159) | (I) oligodendrocytes **Neurological** [^160^](#_ENREF_160)  (I)  cerebral cortex  **ASD**  [^119^](#_ENREF_119) | (I)  lymphoblastoid  **ASD**  [^161^](#_ENREF_161)  (I)  Monocytes  **MDD**  [^162^](#_ENREF_162)  (I)  Peripheral Blood cells  **MDD**  [^163^](#_ENREF_163)    (I)  Serum  **MDD**  [^164^](#_ENREF_164)    (I)  PBMC  **BP**  [^165^](#_ENREF_165)  (I)  PBMC cells  **BP**  [^166^](#_ENREF_166)    (I)  Blood  **Suicide**  [^124^](#_ENREF_124),[^144^](#_ENREF_144)  (I)  Blood  **Pain**  CRPS  [^167^](#_ENREF_167)  (I)  leukocytes  **Stress**  **Social Isolation**  [^168^](#_ENREF_168)    (I)  **Depression-Related**  Cole SW 2015  [^169^](#_ENREF_169)    altered  monocytes  **BP**  [^170^](#_ENREF_170) | **Alcohol**  [^146^](#_ENREF_146) | (I)  NAC  **Phencyclidine**  [^171^](#_ENREF_171)    (I)  PFC (males)  **BP**  [^172^](#_ENREF_172)    (I)  dentate gyrus of hippocampal formation and piriform cortex  **MDD**  [^173^](#_ENREF_173)  (I)  Ventral Hippocampus  **MDD**  [^174^](#_ENREF_174)  (I)  PFC  **Aggression**  [^175^](#_ENREF_175)  (I)  Hypothalamus (PVN)  **Stress**  [^176^](#_ENREF_176)    (I)  PFC (males)  **Stress**  [^172^](#_ENREF_172) |  | 11 |
| **RGS10** regulator of G-protein signaling 10 | 214000_s_at | (I) DE/4 63.5% | 6 | **PTSD** [^177^](#_ENREF_177) | (D)  PFC  **Aging**  [^96^](#_ENREF_96)  (D)  Brain  **BP**  [^178^](#_ENREF_178) | (D)  Blood  **Suicide**  [^144^](#_ENREF_144),[^124^](#_ENREF_124)  (D)  Blood  **Post-Deployment PTSD**  [^177^](#_ENREF_177)  (D)  Blood  **Stress**  **Female specific interpersonal-traumas**  [^179^](#_ENREF_179) |  | (D)  Brain  **SZ**  [^180^](#_ENREF_180)  (D)  AMY (males)  **BP**  [^172^](#_ENREF_172) | (D)  Blood  **Methamphetamine**  [^181^](#_ENREF_181)  (D)  Blood (Females)  **Stress**  [^182^](#_ENREF_182) | 11 |
| **TGFB1 transforming growth factor beta 1** | 203084_at | (D) AP/4 54.5% | 9 | **Suicide**  [^183^](#_ENREF_183)  **Pain**  [^103^](#_ENREF_103),  [^184^](#_ENREF_184)  **Aging** /  **Longevity**  [^185^](#_ENREF_185)  **Depression**  [^186^](#_ENREF_186) | (I)  Frontal and temporal cortex  **ASD**  [^118^](#_ENREF_118)  (I)  OFC  **BP**  [^120^](#_ENREF_120) | (I)  PBMC Cells  **SZ**  [^187^](#_ENREF_187)  (I)  Serum  **SZ**  [^188^](#_ENREF_188)  (I)  Blood  **Depression**  [^186^](#_ENREF_186)  (I)  PBMC  **PTSD**  [^189^](#_ENREF_189)  (I)  CD14+monocytes  **PTSD/Stress**  [^190^](#_ENREF_190)  (I)  PBMCs  **Stress**  [^191^](#_ENREF_191)  (I)  Peripheral blood monocytes  **Chronic** **Stress**  [^145^](#_ENREF_145)  (I) Blood **Stress**[^137^](#_ENREF_137) |  | (I)  Hippocampus  **Stress**  Early Life  [^186^](#_ENREF_186) | (I) Lymphocytes **Phencyclidine** [^171^](#_ENREF_171) | 11 |
| **THRA thyroid hormone receptor, alpha** | 214883_at | (I) DE/4 61.3% | 8 | **SZ**  [^192^](#_ENREF_192),  [^119^](#_ENREF_119) | (D)  Forebrain neural progenitor cells  **SZ**  [^151^](#_ENREF_151)  (D)  PFC  **SZ**  [^193^](#_ENREF_193) | (D)  Blood  **Suicide**  [^124^](#_ENREF_124)  (I)(D)  Blood  **Suicide** (Female)  [^125^](#_ENREF_125)  (D) Blood **Pain** {Niculescu, 2019 #20378}  (I) Blood **Stress**[^137^](#_ENREF_137) |  | (D)  Frontal Cortex  **Alcohol**  [^194^](#_ENREF_194)  (D)  AMY,CP,HIP  **Alcohol**  [^128^](#_ENREF_128)  (D) Female  NAC  **Stress**  [^99^](#_ENREF_99) | (D) Spleen **PTSD**  [^154^](#_ENREF_154) | 11 |
| **VEGFA** vascular endothelial growth factor A | 211527_x_at | (I) DE/2 45.3% | 8 | **SZ**  [^195^](#_ENREF_195)  **Pain MSK**  [^196^](#_ENREF_196)  **MDD**  [^197^](#_ENREF_197)  **Intellect**  [^198^](#_ENREF_198)  **Hallucinations**  [^195^](#_ENREF_195)  **Depression**  [^199^](#_ENREF_199)  **Anxiety**  [^199^](#_ENREF_199) | (D)  CA3/2 Stratum oriens  **BP**  [^200^](#_ENREF_200)  (D)  HIP  **BP**  [^201^](#_ENREF_201)  (D)  AMY and cingulate cortex  **MDD**  [^143^](#_ENREF_143)  (D)  CA1-Stratum oriens  **SZ**  [^200^](#_ENREF_200)  (D)  DLPFC  **SZ**  [^202^](#_ENREF_202)  (D) Male  BA25, NAC, Anterior Insula  ,BA8/9, BA11  **Depression**  [^99^](#_ENREF_99) | (D)  Blood  **Suicide (Females)**  [^125^](#_ENREF_125)  (D)  Blood  **Suicide**  [^203^](#_ENREF_203)  (D)**SZ**  [^204^](#_ENREF_204)  (D)**Completed Suicid** [^205^](#_ENREF_205)  (D) Blood **Pain** {Niculescu, 2019 #20378} |  | (D)  NAC  **Alcohol**  [^128^](#_ENREF_128)  (D)  NAC  **Chronic Stress**  [^206^](#_ENREF_206)  (D)  HIP  **Chronic Stress**  [^207^](#_ENREF_207)  (D) Female  PFC  **Stress**  [^99^](#_ENREF_99) | (D) Plasma **Alcohol** [^105^](#_ENREF_105) | 11 |
| **CEP350** centrosomal protein 350kDa | 204373_s_at | (D) DE/4 67.1% | 6 | **PTSD**  [^208^](#_ENREF_208)  **Depression**  [^209^](#_ENREF_209)  **Autism**  [^119^](#_ENREF_119)  **SZ** [^210^](#_ENREF_210) | (I)  Brain  **BP**  [^178^](#_ENREF_178)  (I)  cerebral cortex  **SZ**  [^119^](#_ENREF_119) | (I)  SH-SY5Y cells  **Cocaine**  [^211^](#_ENREF_211)  (I)  Blood  **Suicide**  [^125^](#_ENREF_125),[^144^](#_ENREF_144),[^124^](#_ENREF_124)  (I) Blood **Pain** {Niculescu, 2019 #20378}  (I) Blood **Stress**[^137^](#_ENREF_137) |  | (I) Male  NAC **Stress** [^99^](#_ENREF_99) |  | 10 |
| **CTSS cathepsin S** | 232617_at | (D) DE/4 56.9% | 8 | **Brain arousal** [^116^](#_ENREF_116) | (I) cerebral cortex **ASD** [^119^](#_ENREF_119) | (I)  PBMCs  **Aging**  [^212^](#_ENREF_212)  (I)  **Aging**  [^150^](#_ENREF_150)  (I)  Blood  **BP**  [^213^](#_ENREF_213)  (I)  Blood  **Suicide**  [^144^](#_ENREF_144)  (I) Blood **Pain** {Niculescu, 2019 #20378} |  | (I)  VT  **Alcohol**  [^214^](#_ENREF_214)  (I)  Hypothalamus  **Aging**  [^215^](#_ENREF_215)  (I)  Hippocampus  **Aging**  [^129^](#_ENREF_129)  (I)  DRG  **Pain**  [^132^](#_ENREF_132) |  | 10 |
| **MAPT microtubule associated protein tau** | 203930_s_at  233117_at  203928_x_at | (I) DE/2 33.7%  (I) DE/2 44.2%  (I) DE/4 57.5% | 10 | **SZ**  [^216^](#_ENREF_216),  [^217^](#_ENREF_217)    **Intellect**  [^198^](#_ENREF_198)    **Alcohol**  [^218^](#_ENREF_218)    **Aging**  [^219^](#_ENREF_219) | (D)  middle temporal gyrus (BA21)  **MDD**  [^220^](#_ENREF_220)  (D)  DLPFC  Cognition  **SZ**  [^221^](#_ENREF_221) | (D)  Blood  **Suicide**  [^124^](#_ENREF_124),[^144^](#_ENREF_144)  (D) Blood **Pain** {Niculescu, 2019 #20378} |  | (D)  HIP,NAC  **Alcohol**  [^128^](#_ENREF_128)  (D)  AMY  **Methamphetamine**  [^222^](#_ENREF_222)  (D)  NAC  **Phencyclidine**  [^171^](#_ENREF_171)  (D) Female,Male  PFC  **Stress**  [^99^](#_ENREF_99) |  | 10 |
| **NPTX2 neuronal pentraxin II** | 213479_at | (I) DE/4 52.5% | 8 | **Mood Disorders NOS,**  **Suicide**  [^223^](#_ENREF_223)    **Brain arousal**  [^116^](#_ENREF_116) | (D)  cerebral cortex  **BP,SZ**  [^119^](#_ENREF_119)  (D)  DLPFC  **BP,MDD,SZ**  [^224^](#_ENREF_224)  (D)  Forebrain neural progenitor cells  **SZ**  [^151^](#_ENREF_151)  (D) Female  BA11,NAC  **Depression**  [^99^](#_ENREF_99)  (D) Male  NAC  **Depression**  [^99^](#_ENREF_99) | (D)  SH-SY5Y cells  **Cocaine**  [^211^](#_ENREF_211)    (D)  PBMCs  **Stress**  [^191^](#_ENREF_191) |  | (D)  CP  **Alcohol**  [^128^](#_ENREF_128)  (D)  Cingulate Cortex  **MDD**  [^225^](#_ENREF_225)    (D) Female  PFC  **Stress**  [^99^](#_ENREF_99) |  | 10 |
| **NRP2**  neuropilin 2 | 222877_at | (I) DE/4 61.3% | 6 | **Longevity** [^226^](#_ENREF_226) | differentially expressed genes FPC **MDD** [^227^](#_ENREF_227) | (D) Blood **Pain** {Niculescu, 2019 #20378} |  | (D)  NAC  **Phencyclidine**  [^171^](#_ENREF_171)  (D) Female  PFC  **Stress**  [^99^](#_ENREF_99) |  | 10 |
| **PER1** period circadian clock 1 | 242832_at | (I) DE/4 61.3% | 6 | **MDD**  [^228^](#_ENREF_228)  **Circadian abnormalities**  [^229^](#_ENREF_229)  **Autism**  [^119^](#_ENREF_119)  **ASD**  [^230^](#_ENREF_230)  **Alcohol**  [^231^](#_ENREF_231) | DLPFC  **MDD** [^232^](#_ENREF_232)  (D)  Anterior Cingulate Cortex  **BP**  [^233^](#_ENREF_233)  (D)  DLFPC  **Suicide**  [^234^](#_ENREF_234)  (D)  Middle temporal gyrus  **SZ**  [^235^](#_ENREF_235) | (D)  Blood  **Alcohol**  [^236^](#_ENREF_236)  (D)  lymphocyte  **SZ**  [^237^](#_ENREF_237)  (D)  Blood  **Suicide**  [^144^](#_ENREF_144),[^124^](#_ENREF_124)  (D)  **Sleep Duration**  [^238^](#_ENREF_238)  (D)  Venous Blood  **PTSD**  [^239^](#_ENREF_239)  (D) Blood **Stress**[^137^](#_ENREF_137) |  | (D)  NAC  **Anxiety**  [^240^](#_ENREF_240)  (D)  Hippocampus  **MDD**  [^241^](#_ENREF_241)  (D)  Hippocampul CA1  **Depression**  [^242^](#_ENREF_242) |  | 10 |
| **UBE2L3** ubiquitin conjugating enzyme E2L 3 | 200682_s_at | (D) DE/6 91% | 4 | **ASD**  [^243^](#_ENREF_243)  **SZ**  [^244^](#_ENREF_244) | (I)  frontal cortex,motor cortex  **Alcohol**  [^97^](#_ENREF_97)  (I)  NAC  **Stress**  [^245^](#_ENREF_245)  (I)  DLPFC, prefrontal cortical parvalbumin neurons cells (PV cells)  **SZ**  [^246^](#_ENREF_246)  (I) Male  BA11, BA8/9, BA25  **Depression**  [^99^](#_ENREF_99) | (I)  PBMCs  **Aging**  [^212^](#_ENREF_212)  (I) Blood **Pain** {Niculescu, 2019 #20378} | **Alcohol** [^146^](#_ENREF_146) |  | (I) C. elegan **Aging** [^247^](#_ENREF_247) | 10 |
| GAP43  growth associated protein 43 | 204471_at | (I) DE/4 50.8% | 7 |  | (D) Brain **BP** [^178^](#_ENREF_178)  (D)  Anterior cingulate cortex  **BP**  [^248^](#_ENREF_248)  (D)  hippocampal hilar region  **BP,SZ**  [^249^](#_ENREF_249)  (D)  Hippocampus  **BP**  [^250^](#_ENREF_250)  (D)  supragenual (BA24) anterior cingulated cortex  **SZ**  [^251^](#_ENREF_251) | (D) Blood **Pain** {Niculescu, 2019 #20378} | **SZ** [^252^](#_ENREF_252) | (D)  Hippocampus  **SZ**  [^253^](#_ENREF_253)  (D)  Caudate putamen  **SZ**  [^254^](#_ENREF_254)  (D)  Hippocampus  **Depression**  [^186^](#_ENREF_186)  (D) Female  NAC  **Stress**  [^99^](#_ENREF_99) |  | 9 |
| **NKTR** natural killer cell triggering receptor | 1570342_at | (D) AP/6 85% | 4 |  | (I)  AMY and cingulate cortex  **MDD**  [^143^](#_ENREF_143)  (I)  DLPFC  **SZ**  [^221^](#_ENREF_221)  (I) Male  Nac  **Depression**  [^99^](#_ENREF_99) | (I)  L neurons  **BP**  [^255^](#_ENREF_255)  (I)  Blood  **Suicide**  [^125^](#_ENREF_125),[^124^](#_ENREF_124)  (I)  leukocytes  **Social Isolation**  [^168^](#_ENREF_168)  (I) Blood **Pain** {Niculescu, 2019 #20378}  (I) Blood **Stress**[^137^](#_ENREF_137) | **Alcohol** [^146^](#_ENREF_146) | (I) Male  Nac **Stress** [^99^](#_ENREF_99) |  | 9 |
| **UBE2I ubiquitin conjugating enzyme E2I** | 233360_at | (D) DE/6 86.8% | 6 |  | (I)  cerebral cortex  **Alcohol**  [^119^](#_ENREF_119)  (I)  Frontal and temporal cortex  **ASD**  [^118^](#_ENREF_118) | (I)  Blood  **Mood State**  [^181^](#_ENREF_181)  (I)  Blood  **Hallucinations**  [^256^](#_ENREF_256)  (I) Blood **Stress**[^137^](#_ENREF_137) |  | (I)  AMY  **Alcohol**  [^257^](#_ENREF_257)  (I) Male  Nac  **Stress**  [^99^](#_ENREF_99) | (I) C. elegan **Aging**  [^247^](#_ENREF_247) | 9 |
| **ARSB arylsulfatase B** | 1554030_at | (I) DE/6 91.7% | 6 |  | (D) Male  BA8/9  ,BA25, Subic  **Depression**  [^99^](#_ENREF_99) | (D)  Blood  **Suicide**  [^124^](#_ENREF_124)  (D) Blood **Stress**[^137^](#_ENREF_137) |  | (D)  HIP,PFC  **Alcohol**  [^128^](#_ENREF_128)  (D)  AMY  **MDD**  [^258^](#_ENREF_258) |  | 8 |
| **CD36** CD36 molecule (thrombospondin receptor) | 242197_x_at | (D) DE/4 56.9% | 6 | **SZ** [^210^](#_ENREF_210) |  | (I)  **Longevity**  [^259^](#_ENREF_259)  (I)  Blood  **Suicide** ^[125](#_ENREF_125)^.[^124^](#_ENREF_124)  (I) Blood **Pain** {Niculescu, 2019 #20378}  (I) Blood **Stress**[^137^](#_ENREF_137) | **Anxiety** [^260^](#_ENREF_260) | (I)  NAC,PFC  **Alcohol**  [^128^](#_ENREF_128)  (I)  AMY  **MDD**  [^174^](#_ENREF_174) | (I)  Blood (Females)  **Stress**  [^182^](#_ENREF_182)  (I)  Adrenal Gland  **Stress**  **Trauma**  [^261^](#_ENREF_261)  (D) C. elegan **Aging** [^247^](#_ENREF_247) | 8 |
| **GFAP** glial fibrillary acidic protein | 203540_at | (I) DE/2 34.3% | 9 |  | (D)  frontal  **Alcohol**  [^262^](#_ENREF_262)  (D)  Frontal cortex  **Alcohol**  [^97^](#_ENREF_97)  (D)  Frontal, motor cortex  **Alcohol**  [^263^](#_ENREF_263)  (D)  ACC (white matter)  **BP**  [^264^](#_ENREF_264)  (D)  PFC (BA-9)  **BP**  [^265^](#_ENREF_265)  (D)  astrocytes  **MDD**  [^266^](#_ENREF_266)  (D)  Cerebellum  **MDD**  [^267^](#_ENREF_267),  [^268^](#_ENREF_268)  (D)  Locus coeruleus (LC)forebrain  **MDD**  [^269^](#_ENREF_269)  (D)  mediodorsal thalamus ,caudate nucleus  **Suicide**  [^270^](#_ENREF_270)  (D)  ACC (white matter)  **SZ**  [^264^](#_ENREF_264)  (D)  Anterior Cingulate Cortex  **SZ**  [^271^](#_ENREF_271)  (D)  Cerebellum  **SZ**  [^272^](#_ENREF_272) | (D) Blood **Suicide**  (Female)  [^125^](#_ENREF_125)  (D) Blood **Pain** {Niculescu, 2019 #20378} |  | (D)  AMY  **Addictions**  [^273^](#_ENREF_273)  (D)  NST  **Yohimbine**  [^274^](#_ENREF_274)  (D)  PFC  **MDD**  [^275^](#_ENREF_275)  (D) Female  PFC  **Stress**  [^99^](#_ENREF_99) |  | 8 |
| **GSTM3 glutathione S-transferase mu 3 (brain)** | 235867_at | (D) DE/4 52.1% | 8 | **BP**  [^276^](#_ENREF_276) | (I)  AMY ACC  **MDD**  [^267^](#_ENREF_267)  (I)  anterior PFC (BA10), superior temporal cortex(BA 22)  **SZ**  [^277^](#_ENREF_277) | (I) Blood **Stress**[^137^](#_ENREF_137) |  |  |  | 8 |
| **GUSB glucuronidase, beta** | 202605_at | (D) DE/4 55.7% | 8 |  | (I)  Frontal Cortex, cerebellum  **Aging**  [^150^](#_ENREF_150) | (I)  **Aging**  [^150^](#_ENREF_150) |  | (I)  Caudate putamen  **Methamphetamine**  [^222^](#_ENREF_222) |  | 8 |
| **ITPKB** inositol-trisphosphate 3-kinase B | 232526_at | (I) DE/4 51.9% | 6 |  | (D)  AMY  **MDD**  [^267^](#_ENREF_267)  (D)  Anterior Cingulate Cortex  **MDD**  [^278^](#_ENREF_278)  (D)  PFC  (BA 46/10)  **Suicide,SZ**  17997842  (D) Anterior PFC **SZA** [^279^](#_ENREF_279) | (D)  Blood  **Alcohol**  [^280^](#_ENREF_280)  (D) Blood  **Aging**  [^150^](#_ENREF_150)  (D) Blood **Stress**[^137^](#_ENREF_137) |  | (D)  NAC  **Phencyclidine**  [^171^](#_ENREF_171)  (D)  PFC  **Stress**  [^99^](#_ENREF_99) |  | 8 |
| **NPC2 Niemann-Pick disease, type C2** | 200701_at | (D) DE/6 80.8% | 8 |  | (I)  Frontal Cortex  **Aging**  [^150^](#_ENREF_150)  (I)  cerebral cortex  **SZ**  [^119^](#_ENREF_119) | (I)  Blood  **Aging**  [^150^](#_ENREF_150)  (I) Blood **Pain** {Niculescu, 2019 #20378}  (D) Blood **Stress**[^137^](#_ENREF_137) |  | (I)  VT  **Alcohol**  [^214^](#_ENREF_214) |  | 8 |
| **FOXO3** forkhead box O3 | 231548_at | (I) AP/2 38.9% (I) DE/6 82.3% | 4 | **Suicide**  [^281^](#_ENREF_281)  **Longevity**  [^226^](#_ENREF_226) [^94^](#_ENREF_94) [^282^](#_ENREF_282) [^283^](#_ENREF_283) |  | (D)  SH-SY5Y cells  **Cocaine**  [^211^](#_ENREF_211)  (D)  Blood  **Suicide**  [^144^](#_ENREF_144) [^124^](#_ENREF_124)  (D) Blood **Pain** {Niculescu, 2019 #20378}  (I) Blood **Stress**[^137^](#_ENREF_137) |  | differentially regulated AMY,HIP **PTSD** [^284^](#_ENREF_284) | differentially regulated  Blood  **PTSD**  [^284^](#_ENREF_284)  (D)  Lymphocytes (males)  **BP,Stress**  [^172^](#_ENREF_172) | 7 |
| **GSK3B glycogen synthase kinase 3 beta** | 209945_s_at  242336_at | (D) DE/4 50.3%  (D) AP/2 34.1% | 10 | **ASD** [^285^](#_ENREF_285)  **Aging** [^286^](#_ENREF_286)    **BP** [^287-289^](#_ENREF_287)  **MDD**  [^290^](#_ENREF_290)  [^291^](#_ENREF_291)  [^292^](#_ENREF_292)  **SZ**  [^293^](#_ENREF_293)  [^294^](#_ENREF_294)  **Suicide**  [^295^](#_ENREF_295) |  | (I)  PBMCs  **Aging**  [^212^](#_ENREF_212)  (I)  peripheral leukocytes  **MDD**  [^296^](#_ENREF_296)  (I)  Fibroblast  **MDD**  [^297^](#_ENREF_297)  (I)  olfactory neurons  **BP**  [^298^](#_ENREF_298)  (I)  L neurons  **BP**  [^255^](#_ENREF_255)  (I)  Blood  **Suicide**  [^124^](#_ENREF_124),[^144^](#_ENREF_144)  (I)  PBMCs  **Stress**  [^191^](#_ENREF_191)  (I) Blood **Pain** {Niculescu, 2019 #20378} | **SZ**  [^293^](#_ENREF_293) | (I)  Accumbens  **Alcohol**  [^299^](#_ENREF_299)  (I)  mesocorticolimbic reward circuit (PFC,NAC, and ventral midbrain)  **Alcohol**  [^300^](#_ENREF_300)  (I)  PFC  **Phencyclidine**  [^171^](#_ENREF_171)  (I)  Hippocampus  **SZ**  [^293^](#_ENREF_293)  (I)  AMY (males)  **BP ,Stress**  [^172^](#_ENREF_172)  (I) Male  PFC  **Stress**  [^99^](#_ENREF_99)  (I) cortex, hippocampus, midbrain, cerebellum **SZ** [^301^](#_ENREF_301)  (I)rods and cones (ERG)  **BP** ,**SZ** [^302^](#_ENREF_302) |  | 7 |
| **RAB7A** RAB7A, member RAS oncogene family | 227602_at | (I) AP/2 43.8% (I) DE/4 69.6% | 7 | **Neuropathic Pain**  [^303^](#_ENREF_303),[^304^](#_ENREF_304)  **Brain arousal**  [^116^](#_ENREF_116) | (D) Female BA11 **Depression** [^99^](#_ENREF_99)  differentially expressed genes FPC **MDD** [^227^](#_ENREF_227)  (D) DLPFC BA 9 **BP** [^305^](#_ENREF_305) |  | **Neuropathic Pain** [^306^](#_ENREF_306) |  |  | 7 |
| **ITPKB** inositol-trisphosphate 3-kinase B | 1554306_at | (D) AP/4 61.1% (D) DE/4 55.7% | 6 |  | (I) Anterior PFC **MDD** [^279^](#_ENREF_279)  (I)  PFC  **Aging**  [^96^](#_ENREF_96)  (I)  Frontal Cortex, cerebellum  **Aging**  [^150^](#_ENREF_150)  (I)  Cerebral Cortex  **ASD**  [^307^](#_ENREF_307)  (I)  Anterior Cingulate Cortex  **BP**  [^278^](#_ENREF_278)  (I)  OFC  **BP**  [^120^](#_ENREF_120)  (I)  cerebral cortex  **BP**  [^119^](#_ENREF_119)  (I)  Cerebellum  **MDD**  [^267^](#_ENREF_267)  (I)  Subcortical,periventricular,medial subcortical white matter  **Neurological**  [^308^](#_ENREF_308)  (I)  PFC  **SZ**  [^309^](#_ENREF_309)  (I)  cerebral cortex  **SZ**  [^119^](#_ENREF_119) | (I)  Blood  **Alcohol**  [^280^](#_ENREF_280)  (I)  Blood  **Suicide**  [^144^](#_ENREF_144),[^124^](#_ENREF_124)  (I)  T cells from blood  **Acute Stress**  [^310^](#_ENREF_310) |  |  |  | 6 |
| **PSEN1 presenilin 1** | 203460_s_at | (D) DE/4 54.5% | 9 | **SZ**  [^311^](#_ENREF_311)  **Depression, Emotional Stability, Neuroticism**  [^312^](#_ENREF_312)    **Autism**  [^119^](#_ENREF_119) |  | (I)  **Aging**  [^150^](#_ENREF_150)  (I)  Blood  **Suicide**  [^124^](#_ENREF_124),[^144^](#_ENREF_144)  (I) Blood **Stress**[^137^](#_ENREF_137) |  | (I)  NAC  **Alcohol**  [^128^](#_ENREF_128) |  | 6 |
| **RHEB** Ras homolog enriched in brain | 243008_at | (D) AP/6 84.4% (D) DE/4 64.1% | 4 | **Suicide**  [^313^](#_ENREF_313)  **Pain**  [^314^](#_ENREF_314) |  | (I) Blood **Suicide** (Females)[^125^](#_ENREF_125)  (I) Blood **Stress**[^137^](#_ENREF_137) |  | (I)  Cerebral Cortex  **SZ-like symptoms**  [^315^](#_ENREF_315) |  | 6 |
| **SERTAD3** SERTA domain containing 3 | 219382_at | (D) DE/6 81.4% | 5 |  | (I)  PFC  **Alcohol**  [^316^](#_ENREF_316)  (I)  cerebral cortex  **ASD**  [^119^](#_ENREF_119) | (I) Blood  **Aging** [^150^](#_ENREF_150)  (I) Blood **Pain** {Niculescu, 2019 #20378} |  |  |  | 6 |
| **TREM2 triggering receptor expressed on myeloid cells 2** | 219725_at | (I) DE/2 37.6% | 11 | **SZ**  [^119^](#_ENREF_119)  **BP,SZ**  [^317^](#_ENREF_317) | (D)  Brain  **BP**  [^178^](#_ENREF_178)  (D)  cerebral cortex  **BP**  [^119^](#_ENREF_119) |  |  |  |  | 6 |
| **NOCT** nocturnin | 220671_at | (D) AP/4 69.5% | 6 | **PTSD** [^177^](#_ENREF_177) |  | (I) Blood **Post-Deployment PTSD** [^177^](#_ENREF_177) |  |  |  | 4 |
| FCGR1A  Fc fragment of IgG, high affinity Ia, receptor (CD64) | 216951_at | (I) DE/4 64.6% | 7 |  |  |  |  |  |  | 0 |

**Table S4 Top Biomarkers (from Figure 3). Evidence for modulation by drugs**

**in same direction as Increased Memory Retention (see also Figure 5).**

| **Genesymbol/Gene name** | **Probesets** | **Step 1  Discovery in Blood  (Direction of Change tracking Memory Increase) Method/ Score/ %  Up to 6pts** | **Step 2  External CFG Evidence For Involvement in AD   Score  Up to 12pts** | **Lithium** | **Omega-3** | **Antidepressants** | **Other Drugs** |
| --- | --- | --- | --- | --- | --- | --- | --- |
| **APOE apolipoprotein E** | 212884_x_at | (D) AP/2 34.1% | 11 |  | (D) Lymphocytes (males)  **Omega-3 fatty acids** [^318^](#_ENREF_318) |  |  |
| **GSK3B glycogen synthase kinase 3 beta** | 242336_at  209945_s_at | (D) AP/2 34.1%  (D) DE/4 50.3% | 10 | (D)  olfactory neurons  **Lithium** [^298^](#_ENREF_298)  **Lithium**^[319](#_ENREF_319)^ | (D)  PFC (females)  **Omega-3 fatty acids** [^318^](#_ENREF_318)  (D) HIP Alzheimer's Disease  **Astaxanthin-DHA** [^43^](#_ENREF_43) |  | (D)  HIP  **Ketamine**[^320^](#_ENREF_320)  (D)HIP **lipoteichoic acid** [^301^](#_ENREF_301)  (D)  Caudate putamen  **Valproate**[^222^](#_ENREF_222)  (D)  Frontal Cortex  **Antipsychotics**[^321^](#_ENREF_321)  **Enzastaurin** |
| **MAPT microtubule associated protein tau** | 203930_s_at  233117_at  203928_x_at | (I) DE/2 33.7%  (I) DE/2 44.2%  (I) DE/4 57.5% | 10 | (I) Schneider 2 (S2) cells   **Lithium** [^322^](#_ENREF_322) | (I)  HIP (males)  **Omega-3 fatty acids** [^318^](#_ENREF_318) |  |  |
| **PTGS2 prostaglandin-endoperoxide synthase 2 (prostaglandin G/H synthase and cyclooxygenase)** | 1554997_a_at | (D) DE/4 76% | 10 | (D)  PBMC  **Lithium**  [^165^](#_ENREF_165) |  |  | (D)  Serum, HIP  **Vorinostat**  [^323^](#_ENREF_323)  (D)  PBMC  **Antipsychotics**  [^165^](#_ENREF_165)  Acetaminophen  NSAIds |
| **GFAP** glial fibrillary acidic protein | 203540_at | (I) DE/2 34.3% | 9 |  | (I)  Brain  **Omega-3 fatty acids** [^324^](#_ENREF_324) |  | (I)  AMY,HIP,PFC  **Clozapine**[^171^](#_ENREF_171) |
| **PSEN1 presenilin 1** | 203460_s_at | (D) DE/4 54.5% | 9 |  | (D) Lymphocytes (females)  **Omega-3 fatty acids**  [^318^](#_ENREF_318) |  | **tarenflurbil** |
| **TGFB1 transforming growth factor beta 1** | 203084_at | (D) AP/4 54.5% | 9 |  | (D) Lymphocytes (females)  **Omega-3 fatty acids** [^318^](#_ENREF_318) |  | **dalantercept,fresolimumab,LY3200882,MSB0011359C** |
| **BACE1**  **Beta-Secretase 1** | 222463_s_at  224335_s_at | (I) DE/6 44.8%  (I) DE/6 43.1% | 8 |  |  |  |  |
| **CTSS cathepsin S** | 232617_at | (D) DE/4 56.9% | 8 |  | (D)  Lymphocytes (females)  **Omega-3 fatty acids** [^318^](#_ENREF_318) |  |  |
| **GUSB glucuronidase, beta** | 202605_at | (D) DE/4 55.7% | 8 |  |  |  | (D)VT **Clozapine**[^171^](#_ENREF_171) |
| **IGF1 insulin-like growth factor 1 (somatomedin C)** | 209542_x_at | (I) DE/4 54.1% | 8 | (I)lymphoblastoid cell lines  **Lithium** [^325^](#_ENREF_325) |  | (I) HIP **Fluoxetine** (**SSRI** ), **Venlafaxine (SNRI)**[^326^](#_ENREF_326) | (I)VT **Clozapine**[^171^](#_ENREF_171) |
| **NPTX2 neuronal pentraxin II** | 213479_at | (I) DE/4 52.5% | 8 |  |  | (I)  HIP  **Fluoxetine**[^327^](#_ENREF_327) | (I)  VT  **Clozapine**[^171^](#_ENREF_171) |
| **THRA thyroid hormone receptor, alpha** | 214883_at | (I) DE/4 61.3% | 8 |  |  |  | **thyroxine** |
| **VEGFA** vascular endothelial growth factor A | 211527_x_at | (I) DE/2 45.3% | 8 |  |  | (I)  Cortex  **Fluoxetine**[^328^](#_ENREF_328) | (I)  Plasma  **Antipsychotics**[^204^](#_ENREF_204)  (I)  Blood  **Steroid**[^329^](#_ENREF_329) |
| **GAP43**  growth associated protein 43 | 204471_at | (I) DE/4 50.8% | 7 |  |  |  | (I) Human astrocyte-derived cells (U-87 MG) **Valproate** [^330^](#_ENREF_330)  (I) HIP **Benzodiazepines**[^331^](#_ENREF_331) |
| **RAB7A** RAB7A, member RAS oncogene family | 227602_at | (I) AP/2 43.8% (I) DE/4 69.6% | 7 |  |  | (I)  basal forebrain  **TCA**  [^332^](#_ENREF_332) | (I)  Caudate putamen  **Valproate**  [^222^](#_ENREF_222) |
| **KIDINS220**  kinase D-interacting substrate 220kDa | 214932_at | (I) DE/4 51.9% | 6 |  |  |  | (I) VT  **Clozapine**[^171^](#_ENREF_171) |
| **CD36** CD36 molecule (thrombospondin receptor) | 242197_x_at | (D) DE/4 56.9% | 6 |  |  |  | (D)  Lymphocytes  **Benzodiazepines**[^331^](#_ENREF_331) |
| **CEP350** centrosomal protein 350kDa | 204373_s_at | (D) DE/4 67.1% | 6 |  |  | (D)  AMY  **Antidepressants, Fluoxetine** [^225^](#_ENREF_225) |  |
| **ITPKB** inositol-trisphosphate 3-kinase B | 1554306_at | (D) AP/4 61.1% (D) DE/4 55.7% | 6 |  | (D)  Lymphocytes (males)  **Omega-3 fatty acids**[^318^](#_ENREF_318) |  |  |
| **NRP2**  neuropilin 2 | 222877_at | (I) DE/4 61.3% | 6 |  |  |  | (I)  CP  **Clozapine**[^171^](#_ENREF_171) |
| **PER1** period circadian clock 1 | 242832_at | (I) DE/4 61.3% | 6 | (I)  Cerebral Cortex (right)  **Lithium**[^333^](#_ENREF_333)  (I)  lymphoblastoid cell lines (LCLs) derived  **Lithium**[^334^](#_ENREF_334) |  |  | (I)  VT  **Clozapine**  [^171^](#_ENREF_171)  (I)  AMY  **Quetiapine**[^335^](#_ENREF_335) |
| **UBE2I ubiquitin conjugating enzyme E2I** | 233360_at | (D) DE/6 86.8% | 6 |  |  |  | (D)VT **Clozapine**[^171^](#_ENREF_171) |
| **FOXO3** forkhead box O3 | 231548_at | (I) AP/2 38.9% (I) DE/6 82.3% | 4 |  |  |  | (I)  Lymphocytes, VT  **Clozapin**^[171](#_ENREF_171)^ |
| **RHEB** Ras homolog enriched in brain | 243008_at | (D) AP/6 84.4% (D) DE/4 64.1% | 4 |  |  |  | (D)  **NR1**[^336^](#_ENREF_336) |
| **UBE2L3** ubiquitin conjugating enzyme E2L 3 | 200682_s_at | (D) DE/6 91% | 4 |  |  |  | (D)VT **Clozapine**[^171^](#_ENREF_171) |

**Literature cited:**

1. Wang S, Jia J. Promoter polymorphisms which modulate BACE1 expression are associated with sporadic Alzheimer's disease. *Am J Med Genet B Neuropsychiatr Genet* 2010; **153B**(1)**:** 159-166.

2. Nowotny P, Kwon JM, Chakraverty S, Nowotny V, Morris JC, Goate AM. Association studies using novel polymorphisms in BACE1 and BACE2. *Neuroreport* 2001; **12**(9)**:** 1799-1802.

3. Randall CN, Strasburger D, Prozonic J, Morris SN, Winkie AD, Parker GR *et al.* Cluster analysis of risk factor genetic polymorphisms in Alzheimer's disease. *Neurochem Res* 2009; **34**(1)**:** 23-28.

4. Cai L, Tang G, Chen L, Zhang B, Jiang S, Ren D. Genetic studies of A2M and BACE1 genes in Chinese Han Alzheimer's disease patients. *Neuroreport* 2005; **16**(9)**:** 1023-1026.

5. Coulson DT, Beyer N, Quinn JG, Brockbank S, Hellemans J, Irvine GB *et al.* BACE1 mRNA expression in Alzheimer's disease postmortem brain tissue. *J Alzheimers Dis* 2010; **22**(4)**:** 1111-1122.

6. Zetterberg H, Andreasson U, Hansson O, Wu G, Sankaranarayanan S, Andersson ME *et al.* Elevated cerebrospinal fluid BACE1 activity in incipient Alzheimer disease. *Arch Neurol* 2008; **65**(8)**:** 1102-1107.

7. Ohno M, Sametsky EA, Younkin LH, Oakley H, Younkin SG, Citron M *et al.* BACE1 deficiency rescues memory deficits and cholinergic dysfunction in a mouse model of Alzheimer's disease. *Neuron* 2004; **41**(1)**:** 27-33.

8. Jiang H, He P, Xie J, Staufenbiel M, Li R, Shen Y. Genetic deletion of TNFRII gene enhances the Alzheimer-like pathology in an APP transgenic mouse model via reduction of phosphorylated IkappaBalpha. *Hum Mol Genet* 2014; **23**(18)**:** 4906-4918.

9. Sharoar MG, Yan R. Effects of altered RTN3 expression on BACE1 activity and Alzheimer's neuritic plaques. *Reviews in the neurosciences* 2017; **28**(2)**:** 145-154.

10. Mez J, Marden JR, Mukherjee S, Walter S, Gibbons LE, Gross AL *et al.* Alzheimer's disease genetic risk variants beyond APOE epsilon4 predict mortality. *Alzheimers Dement (Amst)* 2017; **8:** 188-195.

11. Liu M, Zhang Y, Huo YR, Liu S, Liu S, Wang J *et al.* Influence of the rs1080985 Single Nucleotide Polymorphism of the CYP2D6 Gene and APOE Polymorphism on the Response to Donepezil Treatment in Patients with Alzheimer's Disease in China. *Dementia and geriatric cognitive disorders extra* 2014; **4**(3)**:** 450-456.

12. Genin E, Hannequin D, Wallon D, Sleegers K, Hiltunen M, Combarros O *et al.* APOE and Alzheimer disease: a major gene with semi-dominant inheritance. *Mol Psychiatry* 2011; **16**(9)**:** 903-907.

13. Maloney B, Ge YW, Petersen RC, Hardy J, Rogers JT, Perez-Tur J *et al.* Functional characterization of three single-nucleotide polymorphisms present in the human APOE promoter sequence: Differential effects in neuronal cells and on DNA-protein interactions. *Am J Med Genet B Neuropsychiatr Genet* 2010; **153B**(1)**:** 185-201.

14. Bekris LM, Galloway NM, Montine TJ, Schellenberg GD, Yu CE. APOE mRNA and protein expression in postmortem brain are modulated by an extended haplotype structure. *Am J Med Genet B Neuropsychiatr Genet* 2010; **153B**(2)**:** 409-417.

15. Zubenko GS, Hughes HB, 3rd, Zubenko WN. D10S1423 identifies a susceptibility locus for Alzheimer's disease (AD7) in a prospective, longitudinal, double-blind study of asymptomatic individuals: results at 14 years. *Am J Med Genet B Neuropsychiatr Genet* 2010; **153B**(2)**:** 359-364.

16. Blacker D, Bertram L, Saunders AJ, Moscarillo TJ, Albert MS, Wiener H *et al.* Results of a high-resolution genome screen of 437 Alzheimer's disease families. *Hum Mol Genet* 2003; **12**(1)**:** 23-32.

17. Lee JH, Cheng R, Graff-Radford N, Foroud T, Mayeux R, National Institute on Aging Late-Onset Alzheimer's Disease Family Study G. Analyses of the National Institute on Aging Late-Onset Alzheimer's Disease Family Study: implication of additional loci. *Arch Neurol* 2008; **65**(11)**:** 1518-1526.

18. Butler AW, Ng MY, Hamshere ML, Forabosco P, Wroe R, Al-Chalabi A *et al.* Meta-analysis of linkage studies for Alzheimer's disease--a web resource. *Neurobiol Aging* 2009; **30**(7)**:** 1037-1047.

19. Sillen A, Andrade J, Lilius L, Forsell C, Axelman K, Odeberg J *et al.* Expanded high-resolution genetic study of 109 Swedish families with Alzheimer's disease. *Eur J Hum Genet* 2008; **16**(2)**:** 202-208.

20. Kiddle SJ, Thambisetty M, Simmons A, Riddoch-Contreras J, Hye A, Westman E *et al.* Plasma based markers of [11C] PiB-PET brain amyloid burden. *PLoS ONE* 2012; **7**(9)**:** e44260.

21. Thambisetty M, Tripaldi R, Riddoch-Contreras J, Hye A, An Y, Campbell J *et al.* Proteome-based plasma markers of brain amyloid-beta deposition in non-demented older individuals. *J Alzheimers Dis* 2010; **22**(4)**:** 1099-1109.

22. Mann KM, Thorngate FE, Katoh-Fukui Y, Hamanaka H, Williams DL, Fujita S *et al.* Independent effects of APOE on cholesterol metabolism and brain Abeta levels in an Alzheimer disease mouse model. *Hum Mol Genet* 2004; **13**(17)**:** 1959-1968.

23. Castillo E, Leon J, Mazzei G, Abolhassani N, Haruyama N, Saito T *et al.* Comparative profiling of cortical gene expression in Alzheimer's disease patients and mouse models demonstrates a link between amyloidosis and neuroinflammation. *Sci Rep* 2017; **7**(1)**:** 17762.

24. Guerreiro R, Wojtas A, Bras J, Carrasquillo M, Rogaeva E, Majounie E *et al.* TREM2 variants in Alzheimer's disease. *N Engl J Med* 2013; **368**(2)**:** 117-127.

25. Jonsson T, Stefansson H, Steinberg S, Jonsdottir I, Jonsson PV, Snaedal J *et al.* Variant of TREM2 associated with the risk of Alzheimer's disease. *N Engl J Med* 2013; **368**(2)**:** 107-116.

26. Sims R, van der Lee SJ, Naj AC, Bellenguez C, Badarinarayan N, Jakobsdottir J *et al.* Rare coding variants in PLCG2, ABI3, and TREM2 implicate microglial-mediated innate immunity in Alzheimer's disease. *Nat Genet* 2017; **49**(9)**:** 1373-1384.

27. Carmona S, Zahs K, Wu E, Dakin K, Bras J, Guerreiro R. The role of TREM2 in Alzheimer's disease and other neurodegenerative disorders. *The Lancet Neurology* 2018; **17**(8)**:** 721-730.

28. Lue LF, Schmitz CT, Serrano G, Sue LI, Beach TG, Walker DG. TREM2 Protein Expression Changes Correlate with Alzheimer's Disease Neurodegenerative Pathologies in Post-Mortem Temporal Cortices. *Brain Pathol* 2015; **25**(4)**:** 469-480.

29. Piccio L, Deming Y, Del-Aguila JL, Ghezzi L, Holtzman DM, Fagan AM *et al.* Cerebrospinal fluid soluble TREM2 is higher in Alzheimer disease and associated with mutation status. *Acta Neuropathol* 2016; **131**(6)**:** 925-933.

30. Suarez-Calvet M, Kleinberger G, Araque Caballero MA, Brendel M, Rominger A, Alcolea D *et al.* sTREM2 cerebrospinal fluid levels are a potential biomarker for microglia activity in early-stage Alzheimer's disease and associate with neuronal injury markers. *EMBO molecular medicine* 2016; **8**(5)**:** 466-476.

31. Heslegrave A, Heywood W, Paterson R, Magdalinou N, Svensson J, Johansson P *et al.* Increased cerebrospinal fluid soluble TREM2 concentration in Alzheimer's disease. *Mol Neurodegener* 2016; **11:** 3.

32. Hu N, Tan MS, Yu JT, Sun L, Tan L, Wang YL *et al.* Increased expression of TREM2 in peripheral blood of Alzheimer's disease patients. *J Alzheimers Dis* 2014; **38**(3)**:** 497-501.

33. Casati M, Ferri E, Gussago C, Mazzola P, Abbate C, Bellelli G *et al.* Increased expression of TREM2 in peripheral cells from mild cognitive impairment patients who progress into Alzheimer's disease. *European journal of neurology* 2018; **25**(6)**:** 805-810.

34. Cheng-Hathaway PJ, Reed-Geaghan EG, Jay TR, Casali BT, Bemiller SM, Puntambekar SS *et al.* The Trem2 R47H variant confers loss-of-function-like phenotypes in Alzheimer's disease. *Mol Neurodegener* 2018; **13**(1)**:** 29.

35. Brendel M, Kleinberger G, Probst F, Jaworska A, Overhoff F, Blume T *et al.* Increase of TREM2 during Aging of an Alzheimer's Disease Mouse Model Is Paralleled by Microglial Activation and Amyloidosis. *Frontiers in aging neuroscience* 2017; **9:** 8.

36. Lee CYD, Daggett A, Gu X, Jiang LL, Langfelder P, Li X *et al.* Elevated TREM2 Gene Dosage Reprograms Microglia Responsivity and Ameliorates Pathological Phenotypes in Alzheimer's Disease Models. *Neuron* 2018; **97**(5)**:** 1032-1048 e1035.

37. Kwok JB, Loy CT, Hamilton G, Lau E, Hallupp M, Williams J *et al.* Glycogen synthase kinase-3beta and tau genes interact in Alzheimer's disease. *Ann Neurol* 2008; **64**(4)**:** 446-454.

38. Schaffer BA, Bertram L, Miller BL, Mullin K, Weintraub S, Johnson N *et al.* Association of GSK3B with Alzheimer disease and frontotemporal dementia. *Arch Neurol* 2008; **65**(10)**:** 1368-1374.

39. Mateo I, Infante J, Llorca J, Rodriguez E, Berciano J, Combarros O. Association between glycogen synthase kinase-3beta genetic polymorphism and late-onset Alzheimer's disease. *Dement Geriatr Cogn Disord* 2006; **21**(4)**:** 228-232.

40. Blalock EM, Geddes JW, Chen KC, Porter NM, Markesbery WR, Landfield PW. Incipient Alzheimer's disease: microarray correlation analyses reveal major transcriptional and tumor suppressor responses. *Proc Natl Acad Sci U S A* 2004; **101**(7)**:** 2173-2178.

41. Hye A, Kerr F, Archer N, Foy C, Poppe M, Brown R *et al.* Glycogen synthase kinase-3 is increased in white cells early in Alzheimer's disease. *Neurosci Lett* 2005; **373**(1)**:** 1-4.

42. Kimura T, Yamashita S, Nakao S, Park JM, Murayama M, Mizoroki T *et al.* GSK-3beta is required for memory reconsolidation in adult brain. *PLoS ONE* 2008; **3**(10)**:** e3540.

43. Che H, Li Q, Zhang T, Wang D, Yang L, Xu J *et al.* Effects of Astaxanthin and Docosahexaenoic-Acid-Acylated Astaxanthin on Alzheimer's Disease in APP/PS1 Double-Transgenic Mice. *Journal of agricultural and food chemistry* 2018; **66**(19)**:** 4948-4957.

44. Bullido MJ, Aldudo J, Frank A, Coria F, Avila J, Valdivieso F. A polymorphism in the tau gene associated with risk for Alzheimer's disease. *Neurosci Lett* 2000; **278**(1-2)**:** 49-52.

45. Kaivorinne AL, Kruger J, Kuivaniemi K, Tuominen H, Moilanen V, Majamaa K *et al.* Role of MAPT mutations and haplotype in frontotemporal lobar degeneration in Northern Finland. *BMC Neurol* 2008; **8:** 48.

46. Blasko I, Lederer W, Oberbauer H, Walch T, Kemmler G, Hinterhuber H *et al.* Measurement of thirteen biological markers in CSF of patients with Alzheimer's disease and other dementias. *Dement Geriatr Cogn Disord* 2006; **21**(1)**:** 9-15.

47. Seppala TT, Nerg O, Koivisto AM, Rummukainen J, Puli L, Zetterberg H *et al.* CSF biomarkers for Alzheimer disease correlate with cortical brain biopsy findings. *Neurology* 2012; **78**(20)**:** 1568-1575.

48. Zetterberg H, Wilson D, Andreasson U, Minthon L, Blennow K, Randall J *et al.* Plasma tau levels in Alzheimer's disease. *Alzheimers Res Ther* 2013; **5**(2)**:** 9.

49. De Vos A, Jacobs D, Struyfs H, Fransen E, Andersson K, Portelius E *et al.* C-terminal neurogranin is increased in cerebrospinal fluid but unchanged in plasma in Alzheimer's disease. *Alzheimers Dement* 2015; **11**(12)**:** 1461-1469.

50. Thorsell A, Bjerke M, Gobom J, Brunhage E, Vanmechelen E, Andreasen N *et al.* Neurogranin in cerebrospinal fluid as a marker of synaptic degeneration in Alzheimer's disease. *Brain Res* 2010; **1362:** 13-22.

51. Koedam EL, van der Vlies AE, van der Flier WM, Verwey NA, Koene T, Scheltens P *et al.* Cognitive correlates of cerebrospinal fluid biomarkers in frontotemporal dementia. *Alzheimers Dement* 2013; **9**(3)**:** 269-275.

52. Sjogren M, Rosengren L, Minthon L, Davidsson P, Blennow K, Wallin A. Cytoskeleton proteins in CSF distinguish frontotemporal dementia from AD. *Neurology* 2000; **54**(10)**:** 1960-1964.

53. Mattsson N, Scholl M, Strandberg O, Smith R, Palmqvist S, Insel PS *et al.* (18)F-AV-1451 and CSF T-tau and P-tau as biomarkers in Alzheimer's disease. *EMBO molecular medicine* 2017; **9**(9)**:** 1212-1223.

54. Allen B, Ingram E, Takao M, Smith MJ, Jakes R, Virdee K *et al.* Abundant tau filaments and nonapoptotic neurodegeneration in transgenic mice expressing human P301S tau protein. *J Neurosci* 2002; **22**(21)**:** 9340-9351.

55. Abdullah L, Ait-Ghezala G, Crawford F, Crowell TA, Barker WW, Duara R *et al.* The cyclooxygenase 2 -765 C promoter allele is a protective factor for Alzheimer's disease. *Neurosci Lett* 2006; **395**(3)**:** 240-243.

56. Colangelo V, Schurr J, Ball MJ, Pelaez RP, Bazan NG, Lukiw WJ. Gene expression profiling of 12633 genes in Alzheimer hippocampal CA1: transcription and neurotrophic factor down-regulation and up-regulation of apoptotic and pro-inflammatory signaling. *J Neurosci Res* 2002; **70**(3)**:** 462-473.

57. Yokota O, Terada S, Ishihara T, Nakashima H, Kugo A, Ujike H *et al.* Neuronal expression of cyclooxygenase-2, a pro-inflammatory protein, in the hippocampus of patients with schizophrenia. *Prog Neuropsychopharmacol Biol Psychiatry* 2004; **28**(4)**:** 715-721.

58. Maes OC, Xu S, Yu B, Chertkow HM, Wang E, Schipper HM. Transcriptional profiling of Alzheimer blood mononuclear cells by microarray. *Neurobiol Aging* 2007; **28**(12)**:** 1795-1809.

59. van Rooij JGJ, Meeter LHH, Melhem S, Nijholt DAT, Wong TH, Netherlands Brain B *et al.* Hippocampal transcriptome profiling combined with protein-protein interaction analysis elucidates Alzheimer's disease pathways and genes. *Neurobiol Aging* 2018; **74:** 225-233.

60. Satoh J, Kino Y, Kawana N, Yamamoto Y, Ishida T, Saito Y *et al.* TMEM106B expression is reduced in Alzheimer's disease brains. *Alzheimers Res Ther* 2014; **6**(2)**:** 17.

61. Zahid S, Oellerich M, Asif AR, Ahmed N. Differential expression of proteins in brain regions of Alzheimer's disease patients. *Neurochem Res* 2014; **39**(1)**:** 208-215.

62. Jesse S, Steinacker P, Cepek L, von Arnim CA, Tumani H, Lehnert S *et al.* Glial fibrillary acidic protein and protein S-100B: different concentration pattern of glial proteins in cerebrospinal fluid of patients with Alzheimer's disease and Creutzfeldt-Jakob disease. *J Alzheimers Dis* 2009; **17**(3)**:** 541-551.

63. Oeckl P, Halbgebauer S, Anderl Straub S, Steinacker P, Huss AM, Neugebauer H *et al.* Glial Fibrillary Acidic Protein in Serum Is Increased in Alzheimer's Disease and Correlates with Cognitive Impairment. *J Alzheimers Dis* 2018.

64. Helisalmi S, Hiltunen M, Mannermaa A, Koivisto AM, Lehtovirta M, Alafuzoff I *et al.* Is the presenilin-1 E318G missense mutation a risk factor for Alzheimer's disease? *Neurosci Lett* 2000; **278**(1-2)**:** 65-68.

65. Albani D, Roiter I, Artuso V, Batelli S, Prato F, Pesaresi M *et al.* Presenilin-1 mutation E318G and familial Alzheimer's disease in the Italian population. *Neurobiol Aging* 2007; **28**(11)**:** 1682-1688.

66. Belbin O, Beaumont H, Warden D, Smith AD, Kalsheker N, Morgan K. PSEN1 polymorphisms alter the rate of cognitive decline in sporadic Alzheimer's disease patients. *Neurobiol Aging* 2009; **30**(12)**:** 1992-1999.

67. Hsu S, Gordon BA, Hornbeck R, Norton JB, Levitch D, Louden A *et al.* Discovery and validation of autosomal dominant Alzheimer's disease mutations. *Alzheimers Res Ther* 2018; **10**(1)**:** 67.

68. Duff K, Eckman C, Zehr C, Yu X, Prada CM, Perez-tur J *et al.* Increased amyloid-beta42(43) in brains of mice expressing mutant presenilin 1. *Nature* 1996; **383**(6602)**:** 710-713.

69. Oddo S, Caccamo A, Shepherd JD, Murphy MP, Golde TE, Kayed R *et al.* Triple-transgenic model of Alzheimer's disease with plaques and tangles: intracellular Abeta and synaptic dysfunction. *Neuron* 2003; **39**(3)**:** 409-421.

70. Kiyota T, Morrison CM, Tu G, Dyavarshetty B, Weir RA, Zhang G *et al.* Presenilin-1 familial Alzheimer's disease mutation alters hippocampal neurogenesis and memory function in CCL2 null mice. *Brain Behav Immun* 2015; **49:** 311-321.

71. Arosio B, Bergamaschini L, Galimberti L, La Porta C, Zanetti M, Calabresi C *et al.* +10 T/C polymorphisms in the gene of transforming growth factor-beta1 are associated with neurodegeneration and its clinical evolution. *Mech Ageing Dev* 2007; **128**(10)**:** 553-557.

72. Luedecking EK, DeKosky ST, Mehdi H, Ganguli M, Kamboh MI. Analysis of genetic polymorphisms in the transforming growth factor-beta1 gene and the risk of Alzheimer's disease. *Hum Genet* 2000; **106**(5)**:** 565-569.

73. Gaertner RF, Wyss-Coray T, Von Euw D, Lesne S, Vivien D, Lacombe P. Reduced brain tissue perfusion in TGF-beta 1 transgenic mice showing Alzheimer's disease-like cerebrovascular abnormalities. *Neurobiol Dis* 2005; **19**(1-2)**:** 38-46.

74. Grupe A, Abraham R, Li Y, Rowland C, Hollingworth P, Morgan A *et al.* Evidence for novel susceptibility genes for late-onset Alzheimer's disease from a genome-wide association study of putative functional variants. *Hum Mol Genet* 2007; **16**(8)**:** 865-873.

75. Hong GS, Heun R, Jessen F, Popp J, Hentschel F, Kelemen P *et al.* Gene variations in GSTM3 are a risk factor for Alzheimer's disease. *Neurobiol Aging* 2009; **30**(5)**:** 691-696.

76. Maes OC, Schipper HM, Chong G, Chertkow HM, Wang E. A GSTM3 polymorphism associated with an etiopathogenetic mechanism in Alzheimer disease. *Neurobiol Aging* 2010; **31**(1)**:** 34-45.

77. Liu F, Arias-Vasquez A, Sleegers K, Aulchenko YS, Kayser M, Sanchez-Juan P *et al.* A genomewide screen for late-onset Alzheimer disease in a genetically isolated Dutch population. *Am J Hum Genet* 2007; **81**(1)**:** 17-31.

78. Giedraitis V, Kilander L, Degerman-Gunnarsson M, Sundelof J, Axelsson T, Syvanen AC *et al.* Genetic analysis of Alzheimer's disease in the Uppsala Longitudinal Study of Adult Men. *Dement Geriatr Cogn Disord* 2009; **27**(1)**:** 59-68.

79. Wollmer MA, Sleegers K, Ingelsson M, Zekanowski C, Brouwers N, Maruszak A *et al.* Association study of cholesterol-related genes in Alzheimer's disease. *Neurogenetics* 2007; **8**(3)**:** 179-188.

80. Dayon L, Nunez Galindo A, Wojcik J, Cominetti O, Corthesy J, Oikonomidi A *et al.* Alzheimer disease pathology and the cerebrospinal fluid proteome. *Alzheimers Res Ther* 2018; **10**(1)**:** 66.

81. Goumidi L, Flamant F, Lendon C, Galimberti D, Pasquier F, Scarpini E *et al.* Study of thyroid hormone receptor alpha gene polymorphisms on Alzheimer's disease. *Neurobiol Aging* 2011; **32**(4)**:** 624-630.

82. Chiappelli M, Borroni B, Archetti S, Calabrese E, Corsi MM, Franceschi M *et al.* VEGF gene and phenotype relation with Alzheimer's disease and mild cognitive impairment. *Rejuvenation research* 2006; **9**(4)**:** 485-493.

83. Del Bo R, Scarlato M, Ghezzi S, Martinelli Boneschi F, Fenoglio C, Galbiati S *et al.* Vascular endothelial growth factor gene variability is associated with increased risk for AD. *Ann Neurol* 2005; **57**(3)**:** 373-380.

84. Yuan Q, Zuo X, Jia J. Association between promoter polymorphisms of vascular endothelial growth factor gene and sporadic Alzheimer's disease among Northern Chinese Han. *Neurosci Lett* 2009; **457**(3)**:** 133-136.

85. Sjogren M, Davidsson P, Gottfries J, Vanderstichele H, Edman A, Vanmechelen E *et al.* The cerebrospinal fluid levels of tau, growth-associated protein-43 and soluble amyloid precursor protein correlate in Alzheimer's disease, reflecting a common pathophysiological process. *Dement Geriatr Cogn Disord* 2001; **12**(4)**:** 257-264.

86. Potkin SG, Guffanti G, Lakatos A, Turner JA, Kruggel F, Fallon JH *et al.* Hippocampal atrophy as a quantitative trait in a genome-wide association study identifying novel susceptibility genes for Alzheimer's disease. *PLoS ONE* 2009; **4**(8)**:** e6501.

87. Berchtold NC, Sabbagh MN, Beach TG, Kim RC, Cribbs DH, Cotman CW. Brain gene expression patterns differentiate mild cognitive impairment from normal aged and Alzheimer's disease. *Neurobiol Aging* 2014; **35**(9)**:** 1961-1972.

88. Taguchi K, Yamagata HD, Zhong W, Kamino K, Akatsu H, Hata R *et al.* Identification of hippocampus-related candidate genes for Alzheimer's disease. *Ann Neurol* 2005; **57**(4)**:** 585-588.

89. Garranzo-Asensio M, San Segundo-Acosta P, Martinez-Useros J, Montero-Calle A, Fernandez-Acenero MJ, Haggmark-Manberg A *et al.* Identification of prefrontal cortex protein alterations in Alzheimer's disease. *Oncotarget* 2018; **9**(13)**:** 10847-10867.

90. Emilsson L, Saetre P, Jazin E. Alzheimer's disease: mRNA expression profiles of multiple patients show alterations of genes involved with calcium signaling. *Neurobiol Dis* 2006; **21**(3)**:** 618-625.

91. Wang X, Lopez OL, Sweet RA, Becker JT, DeKosky ST, Barmada MM *et al.* Genetic determinants of disease progression in Alzheimer's disease. *J Alzheimers Dis* 2015; **43**(2)**:** 649-655.

92. Chen CM, Hou YT, Liu JY, Wu YR, Lin CH, Fung HC *et al.* PPP2R2B CAG repeat length in the Han Chinese in Taiwan: Association analyses in neurological and psychiatric disorders and potential functional implications. *Am J Med Genet B Neuropsychiatr Genet* 2009; **150B**(1)**:** 124-129.

93. Ahn K, Song JH, Kim DK, Park MH, Jo SA, Koh YH. Ubc9 gene polymorphisms and late-onset Alzheimer's disease in the Korean population: a genetic association study. *Neurosci Lett* 2009; **465**(3)**:** 272-275.

94. Deelen J, Uh HW, Monajemi R, van Heemst D, Thijssen PE, Bohringer S *et al.* Gene set analysis of GWAS data for human longevity highlights the relevance of the insulin/IGF-1 signaling and telomere maintenance pathways. *Age (Dordr)* 2013; **35**(1)**:** 235-249.

95. Pereira AC, McQuillin A, Puri V, Anjorin A, Bass N, Kandaswamy R *et al.* Genetic association and sequencing of the insulin-like growth factor 1 gene in bipolar affective disorder. *Am J Med Genet B Neuropsychiatr Genet* 2011; **156**(2)**:** 177-187.

96. Erraji-Benchekroun L, Underwood MD, Arango V, Galfalvy H, Pavlidis P, Smyrniotopoulos P *et al.* Molecular aging in human prefrontal cortex is selective and continuous throughout adult life. *Biol Psychiatry* 2005; **57**(5)**:** 549-558.

97. Liu J, Lewohl JM, Dodd PR, Randall PK, Harris RA, Mayfield RD. Gene expression profiling of individual cases reveals consistent transcriptional changes in alcoholic human brain. *J Neurochem* 2004; **90**(5)**:** 1050-1058.

98. Fromer M, Roussos P, Sieberts SK, Johnson JS, Kavanagh DH, Perumal TM *et al.* Gene expression elucidates functional impact of polygenic risk for schizophrenia. *Nature neuroscience* 2016; **19**(11)**:** 1442-1453.

99. Labonte B, Engmann O, Purushothaman I, Menard C, Wang J, Tan C *et al.* Sex-specific transcriptional signatures in human depression. *Nat Med* 2017; **23**(9)**:** 1102-1111.

100. Sebastiani P, Thyagarajan B, Sun F, Schupf N, Newman AB, Montano M *et al.* Biomarker signatures of aging. *Aging Cell* 2017; **16**(2)**:** 329-338.

101. Alisch RS, Van Hulle C, Chopra P, Bhattacharyya A, Zhang SC, Davidson RJ *et al.* A multi-dimensional characterization of anxiety in monozygotic twin pairs reveals susceptibility loci in humans. *Translational psychiatry* 2017; **7**(12)**:** 1282.

102. Mitschelen M, Yan H, Farley JA, Warrington JP, Han S, Herenu CB *et al.* Long-term deficiency of circulating and hippocampal insulin-like growth factor I induces depressive behavior in adult mice: a potential model of geriatric depression. *Neuroscience* 2011; **185:** 50-60.

103. Zorina-Lichtenwalter K, Meloto CB, Khoury S, Diatchenko L. Genetic predictors of human chronic pain conditions. *Neuroscience* 2016; **338:** 36-62.

104. Muhie S, Gautam A, Meyerhoff J, Chakraborty N, Hammamieh R, Jett M. Brain transcriptome profiles in mouse model simulating features of post-traumatic stress disorder. *Mol Brain* 2015; **8:** 14.

105. Freeman WM, Salzberg AC, Gonzales SW, Grant KA, Vrana KE. Classification of alcohol abuse by plasma protein biomarkers. *Biol Psychiatry* 2010; **68**(3)**:** 219-222.

106. Merritt VC, Clark AL, Sorg SF, Evangelista ND, Werhane M, Bondi MW *et al.* Apolipoprotein E epsilon4 Genotype Is Associated with Elevated Psychiatric Distress in Veterans with a History of Mild to Moderate Traumatic Brain Injury. *Journal of neurotrauma* 2018; **35**(19)**:** 2272-2282.

107. Hwang JP, Yang CH, Hong CJ, Lirng JF, Yang YM, Tsai SJ. Association of APOE genetic polymorphism with cognitive function and suicide history in geriatric depression. *Dement Geriatr Cogn Disord* 2006; **22**(4)**:** 334-338.

108. Saiz PA, Garcia-Portilla P, Paredes B, Corcoran P, Arango C, Morales B *et al.* Role of serotonergic-related systems in suicidal behavior: Data from a case-control association study. *Prog Neuropsychopharmacol Biol Psychiatry* 2011; **35**(6)**:** 1518-1524.

109. Mota NP, Han S, Harpaz-Rotem I, Maruff P, Krystal JH, Southwick SM *et al.* Apolipoprotein E gene polymorphism, trauma burden, and posttraumatic stress symptoms in U.S. military veterans: Results from the National Health and Resilience in Veterans Study. *Depress Anxiety* 2018; **35**(2)**:** 168-177.

110. Reeser JC, Payne E, Kitchner T, McCarty CA. Apolipoprotein e4 genotype increases the risk of being diagnosed with posttraumatic fibromyalgia. *PM & R : the journal of injury, function, and rehabilitation* 2011; **3**(3)**:** 193-197.

111. Sebastiani P, Solovieff N, Dewan AT, Walsh KM, Puca A, Hartley SW *et al.* Genetic signatures of exceptional longevity in humans. *PLoS ONE* 2012; **7**(1)**:** e29848.

112. Davies G, Harris SE, Reynolds CA, Payton A, Knight HM, Liewald DC *et al.* A genome-wide association study implicates the APOE locus in nonpathological cognitive ageing. *Mol Psychiatry* 2014; **19**(1)**:** 76-87.

113. Arpawong TE, Pendleton N, Mekli K, McArdle JJ, Gatz M, Armoskus C *et al.* Genetic variants specific to aging-related verbal memory: Insights from GWASs in a population-based cohort. *PLoS ONE* 2017; **12**(8)**:** e0182448.

114. Rajan KB, Wilson RS, Skarupski KA, Mendes de Leon CF, Evans DA. Gene-behavior interaction of depressive symptoms and the apolipoprotein E {varepsilon}4 allele on cognitive decline. *Psychosom Med* 2014; **76**(2)**:** 101-108.

115. Lopez-Leon S, Janssens AC, Gonzalez-Zuloeta Ladd AM, Del-Favero J, Claes SJ, Oostra BA *et al.* Meta-analyses of genetic studies on major depressive disorder. *Mol Psychiatry* 2008; **13**(8)**:** 772-785.

116. Jawinski P, Kirsten H, Sander C, Spada J, Ulke C, Huang J *et al.* Human brain arousal in the resting state: a genome-wide association study. *Molecular psychiatry* 2018.

117. Craig D, Hart DJ, McCool K, McIlroy SP, Passmore AP. Apolipoprotein E e4 allele influences aggressive behaviour in Alzheimer's disease. *J Neurol Neurosurg Psychiatry* 2004; **75**(9)**:** 1327-1330.

118. Parikshak NN, Swarup V, Belgard TG, Irimia M, Ramaswami G, Gandal MJ *et al.* Genome-wide changes in lncRNA, splicing, and regional gene expression patterns in autism. *Nature* 2016; **540**(7633)**:** 423-427.

119. Gandal MJ, Haney JR, Parikshak NN, Leppa V, Ramaswami G, Hartl C *et al.* Shared molecular neuropathology across major psychiatric disorders parallels polygenic overlap. *Science* 2018; **359**(6376)**:** 693-697.

120. Darby MM, Yolken RH, Sabunciyan S. Consistently altered expression of gene sets in postmortem brains of individuals with major psychiatric disorders. *Transl Psychiatry* 2016; **6**(9)**:** e890.

121. Wang J, Qu S, Wang W, Guo L, Zhang K, Chang S *et al.* A combined analysis of genome-wide expression profiling of bipolar disorder in human prefrontal cortex. *Journal of psychiatric research* 2016; **82:** 23-29.

122. Scarr E, Udawela M, Thomas EA, Dean B. Changed gene expression in subjects with schizophrenia and low cortical muscarinic M1 receptors predicts disrupted upstream pathways interacting with that receptor. *Mol Psychiatry* 2018; **23**(2)**:** 295-303.

123. Dean B, Laws SM, Hone E, Taddei K, Scarr E, Thomas EA *et al.* Increased levels of apolipoprotein E in the frontal cortex of subjects with schizophrenia. *Biol Psychiatry* 2003; **54**(6)**:** 616-622.

124. Niculescu AB, Le-Niculescu H, Levey DF, Phalen PL, Dainton HL, Roseberry K *et al.* Precision medicine for suicidality: from universality to subtypes and personalization. *Mol Psychiatry* 2017; **22**(9)**:** 1250-1273.

125. Levey DF, Niculescu EM, Le-Niculescu H, Dainton HL, Phalen PL, Ladd TB *et al.* Towards understanding and predicting suicidality in women: biomarkers and clinical risk assessment. *Mol Psychiatry* 2016; **21**(6)**:** 768-785.

126. Nielsen DA, Spellicy CJ, Harding MJ, Graham DP. Apolipoprotein E DNA methylation and posttraumatic stress disorder are associated with plasma ApoE level: A preliminary study. *Behav Brain Res* 2019; **356:** 415-422.

127. McBride WJ, Kimpel MW, Schultz JA, McClintick JN, Edenberg HJ, Bell RL. Changes in gene expression in regions of the extended amygdala of alcohol-preferring rats after binge-like alcohol drinking. *Alcohol* 2010; **44**(2)**:** 171-183.

128. Rodd ZA, Bertsch BA, Strother WN, Le-Niculescu H, Balaraman Y, Hayden E *et al.* Candidate genes, pathways and mechanisms for alcoholism: an expanded convergent functional genomics approach. *Pharmacogenomics J* 2007; **7**(4)**:** 222-256.

129. Terao A, Apte-Deshpande A, Dousman L, Morairty S, Eynon BP, Kilduff TS *et al.* Immune response gene expression increases in the aging murine hippocampus. *J Neuroimmunol* 2002; **132**(1-2)**:** 99-112.

130. Liu M, Fitzgibbon M, Wang Y, Reilly J, Qian X, O'Brien T *et al.* Ulk4 regulates GABAergic signaling and anxiety-related behavior. *Transl Psychiatry* 2018; **8**(1)**:** 43.

131. Descalzi G, Mitsi V, Purushothaman I, Gaspari S, Avrampou K, Loh YE *et al.* Neuropathic pain promotes adaptive changes in gene expression in brain networks involved in stress and depression. *Sci Signal* 2017; **10**(471).

132. LaCroix-Fralish ML, Austin JS, Zheng FY, Levitin DJ, Mogil JS. Patterns of pain: meta-analysis of microarray studies of pain. *Pain* 2011; **152**(8)**:** 1888-1898.

133. Lee HC, Chang DE, Yeom M, Kim GH, Choi KD, Shim I *et al.* Gene expression profiling in hypothalamus of immobilization-stressed mouse using cDNA microarray. *Brain Res Mol Brain Res* 2005; **135**(1-2)**:** 293-300.

134. Carboni L, Becchi S, Piubelli C, Mallei A, Giambelli R, Razzoli M *et al.* Early-life stress and antidepressants modulate peripheral biomarkers in a gene-environment rat model of depression. *Prog Neuropsychopharmacol Biol Psychiatry* 2010; **34**(6)**:** 1037-1048.

135. Tombacz D, Maroti Z, Kalmar T, Csabai Z, Balazs Z, Takahashi S *et al.* High-Coverage Whole-Exome Sequencing Identifies Candidate Genes for Suicide in Victims with Major Depressive Disorder. *Sci Rep* 2017; **7**(1)**:** 7106.

136. Sequeira A, Klempan T, Canetti L, ffrench-Mullen J, Benkelfat C, Rouleau GA *et al.* Patterns of gene expression in the limbic system of suicides with and without major depression. *Mol Psychiatry* 2007; **12**(7)**:** 640-655.

137. Le-Niculescu H, Roseberry K, Levey DF, Rogers J, Kosary K, Prabha S *et al.* Towards precision medicine for stress disorders: diagnostic biomarkers and targeted drugs. *Molecular psychiatry* 2019.

138. Savonenko AV, Melnikova T, Laird FM, Stewart KA, Price DL, Wong PC. Alteration of BACE1-dependent NRG1/ErbB4 signaling and schizophrenia-like phenotypes in BACE1-null mice. *Proc Natl Acad Sci U S A* 2008; **105**(14)**:** 5585-5590.

139. Kranz TM, Berns A, Shields J, Rothman K, Walsh-Messinger J, Goetz RR *et al.* Phenotypically distinct subtypes of psychosis accompany novel or rare variants in four different signaling genes. *EBioMedicine* 2016; **6:** 206-214.

140. Livshits G, Malkin I, Freidin MB, Xia Y, Gao F, Wang J *et al.* Genome-wide methylation analysis of a large population sample shows neurological pathways involvement in chronic widespread musculoskeletal pain. *Pain* 2017; **158**(6)**:** 1053-1062.

141. McClintick JN, Xuei X, Tischfield JA, Goate A, Foroud T, Wetherill L *et al.* Stress-response pathways are altered in the hippocampus of chronic alcoholics. *Alcohol* 2013; **47**(7)**:** 505-515.

142. Sokolov BP, Jiang L, Trivedi NS, Aston C. Transcription profiling reveals mitochondrial, ubiquitin and signaling systems abnormalities in postmortem brains from subjects with a history of alcohol abuse or dependence. *J Neurosci Res* 2003; **72**(6)**:** 756-767.

143. Gaiteri C, Guilloux JP, Lewis DA, Sibille E. Altered gene synchrony suggests a combined hormone-mediated dysregulated state in major depression. *PLoS ONE* 2010; **5**(4)**:** e9970.

144. Niculescu AB, Levey DF, Phalen PL, Le-Niculescu H, Dainton HD, Jain N *et al.* Understanding and predicting suicidality using a combined genomic and clinical risk assessment approach. *Mol Psychiatry* 2015; **20**(11)**:** 1266-1285.

145. Miller GE, Chen E, Sze J, Marin T, Arevalo JM, Doll R *et al.* A functional genomic fingerprint of chronic stress in humans: blunted glucocorticoid and increased NF-kappaB signaling. *Biol Psychiatry* 2008; **64**(4)**:** 266-272.

146. Lo CL, Lossie AC, Liang T, Liu Y, Xuei X, Lumeng L *et al.* High Resolution Genomic Scans Reveal Genetic Architecture Controlling Alcohol Preference in Bidirectionally Selected Rat Model. *PLoS Genet* 2016; **12**(8)**:** e1006178.

147. Rampino A, Di Carlo P, Fazio L, Ursini G, Pergola G, De Virgilio C *et al.* Association of functional genetic variation in PP2A with prefrontal working memory processing. *Behav Brain Res* 2017; **316:** 125-130.

148. Treutlein J, Cichon S, Ridinger M, Wodarz N, Soyka M, Zill P *et al.* Genome-wide association study of alcohol dependence. *Arch Gen Psychiatry* 2009; **66**(7)**:** 773-784.

149. Zayats T, Jacobsen KK, Kleppe R, Jacob CP, Kittel-Schneider S, Ribases M *et al.* Exome chip analyses in adult attention deficit hyperactivity disorder. *Transl Psychiatry* 2016; **6**(10)**:** e923.

150. Peters MJ, Joehanes R, Pilling LC, Schurmann C, Conneely KN, Powell J *et al.* The transcriptional landscape of age in human peripheral blood. *Nature communications* 2015; **6:** 8570.

151. Roussos P, Guennewig B, Kaczorowski DC, Barry G, Brennand KJ. Activity-Dependent Changes in Gene Expression in Schizophrenia Human-Induced Pluripotent Stem Cell Neurons. *JAMA Psychiatry* 2016; **73**(11)**:** 1180-1188.

152. Brennand KJ, Simone A, Jou J, Gelboin-Burkhart C, Tran N, Sangar S *et al.* Modelling schizophrenia using human induced pluripotent stem cells. *Nature* 2011; **473**(7346)**:** 221-225.

153. Moller-Levet CS, Archer SN, Bucca G, Laing EE, Slak A, Kabiljo R *et al.* Effects of insufficient sleep on circadian rhythmicity and expression amplitude of the human blood transcriptome. *Proc Natl Acad Sci U S A* 2013; **110**(12)**:** E1132-1141.

154. Muhie S, Gautam A, Chakraborty N, Hoke A, Meyerhoff J, Hammamieh R *et al.* Molecular indicators of stress-induced neuroinflammation in a mouse model simulating features of post-traumatic stress disorder. *Transl Psychiatry* 2017; **7**(5)**:** e1135.

155. Uhl GR, Drgon T, Johnson C, Fatusin OO, Liu QR, Contoreggi C *et al.* "Higher order" addiction molecular genetics: convergent data from genome-wide association in humans and mice. *Biochem Pharmacol* 2008; **75**(1)**:** 98-111.

156. Lee PH, Perlis RH, Jung JY, Byrne EM, Rueckert E, Siburian R *et al.* Multi-locus genome-wide association analysis supports the role of glutamatergic synaptic transmission in the etiology of major depressive disorder. *Transl Psychiatry* 2012; **2:** e184.

157. Su KP, Huang SY, Peng CY, Lai HC, Huang CL, Chen YC *et al.* Phospholipase A2 and cyclooxygenase 2 genes influence the risk of interferon-alpha-induced depression by regulating polyunsaturated fatty acids levels. *Biol Psychiatry* 2010; **67**(6)**:** 550-557.

158. Schlauch KA, Khaiboullina SF, De Meirleir KL, Rawat S, Petereit J, Rizvanov AA *et al.* Genome-wide association analysis identifies genetic variations in subjects with myalgic encephalomyelitis/chronic fatigue syndrome. *Transl Psychiatry* 2016; **6:** e730.

159. Reyes-Gibby CC, Swartz MD, Yu X, Wu X, Yennurajalingam S, Anderson KO *et al.* Symptom clusters of pain, depressed mood, and fatigue in lung cancer: assessing the role of cytokine genes. *Supportive care in cancer : official journal of the Multinational Association of Supportive Care in Cancer* 2013; **21**(11)**:** 3117-3125.

160. Rose JW, Hill KE, Watt HE, Carlson NG. Inflammatory cell expression of cyclooxygenase-2 in the multiple sclerosis lesion. *J Neuroimmunol* 2004; **149**(1-2)**:** 40-49.

161. Hu VW, Frank BC, Heine S, Lee NH, Quackenbush J. Gene expression profiling of lymphoblastoid cell lines from monozygotic twins discordant in severity of autism reveals differential regulation of neurologically relevant genes. *BMC Genomics* 2006; **7:** 118.

162. Carvalho LA, Bergink V, Sumaski L, Wijkhuijs J, Hoogendijk WJ, Birkenhager TK *et al.* Inflammatory activation is associated with a reduced glucocorticoid receptor alpha/beta expression ratio in monocytes of inpatients with melancholic major depressive disorder. *Transl Psychiatry* 2014; **4:** e344.

163. Galecki P, Galecka E, Maes M, Chamielec M, Orzechowska A, Bobinska K *et al.* The expression of genes encoding for COX-2, MPO, iNOS, and sPLA2-IIA in patients with recurrent depressive disorder. *J Affect Disord* 2012; **138**(3)**:** 360-366.

164. Galecki P, Talarowska M, Bobinska K, Szemraj J. COX-2 gene expression is correlated with cognitive function in recurrent depressive disorder. *Psychiatry Res* 2014; **215**(2)**:** 488-490.

165. Padmos RC, Hillegers MH, Knijff EM, Vonk R, Bouvy A, Staal FJ *et al.* A discriminating messenger RNA signature for bipolar disorder formed by an aberrant expression of inflammatory genes in monocytes. *Arch Gen Psychiatry* 2008; **65**(4)**:** 395-407.

166. Gurvich A, Begemann M, Dahm L, Sargin D, Miskowiak K, Ehrenreich H. A role for prostaglandins in rapid cycling suggested by episode-specific gene expression shifts in peripheral blood mononuclear cells: a preliminary report. *Bipolar Disord* 2014; **16**(8)**:** 881-888.

167. Jin EH, Zhang E, Ko Y, Sim WS, Moon DE, Yoon KJ *et al.* Genome-wide expression profiling of complex regional pain syndrome. *PLoS ONE* 2013; **8**(11)**:** e79435.

168. Cole SW, Hawkley LC, Arevalo JM, Sung CY, Rose RM, Cacioppo JT. Social regulation of gene expression in human leukocytes. *Genome Biol* 2007; **8**(9)**:** R189.

169. Cole SW, Levine ME, Arevalo JM, Ma J, Weir DR, Crimmins EM. Loneliness, eudaimonia, and the human conserved transcriptional response to adversity. *Psychoneuroendocrinology* 2015; **62:** 11-17.

170. Padmos RC, Van Baal GC, Vonk R, Wijkhuijs AJ, Kahn RS, Nolen WA *et al.* Genetic and environmental influences on pro-inflammatory monocytes in bipolar disorder: a twin study. *Arch Gen Psychiatry* 2009; **66**(9)**:** 957-965.

171. Le-Niculescu H, Balaraman Y, Patel S, Tan J, Sidhu K, Jerome RE *et al.* Towards understanding the schizophrenia code: an expanded convergent functional genomics approach. *Am J Med Genet B Neuropsychiatr Genet* 2007; **144B**(2)**:** 129-158.

172. Le-Niculescu H, McFarland MJ, Ogden CA, Balaraman Y, Patel S, Tan J *et al.* Phenomic, convergent functional genomic, and biomarker studies in a stress-reactive genetic animal model of bipolar disorder and co-morbid alcoholism. *Am J Med Genet B Neuropsychiatr Genet* 2008; **147B**(2)**:** 134-166.

173. Sun W, Park KW, Choe J, Rhyu IJ, Kim IH, Park SK *et al.* Identification of novel electroconvulsive shock-induced and activity-dependent genes in the rat brain. *Biochem Biophys Res Commun* 2005; **327**(3)**:** 848-856.

174. Bagot RC, Cates HM, Purushothaman I, Lorsch ZS, Walker DM, Wang J *et al.* Circuit-wide Transcriptional Profiling Reveals Brain Region-Specific Gene Networks Regulating Depression Susceptibility. *Neuron* 2016; **90**(5)**:** 969-983.

175. Malki K, Tosto MG, Pain O, Sluyter F, Mineur YS, Crusio WE *et al.* Comparative mRNA analysis of behavioral and genetic mouse models of aggression. *American journal of medical genetics Part B, Neuropsychiatric genetics : the official publication of the International Society of Psychiatric Genetics* 2016; **171B**(3)**:** 427-436.

176. Reyes TM, Walker JR, DeCino C, Hogenesch JB, Sawchenko PE. Categorically distinct acute stressors elicit dissimilar transcriptional profiles in the paraventricular nucleus of the hypothalamus. *J Neurosci* 2003; **23**(13)**:** 5607-5616.

177. Breen MS, Maihofer AX, Glatt SJ, Tylee DS, Chandler SD, Tsuang MT *et al.* Gene networks specific for innate immunity define post-traumatic stress disorder. *Mol Psychiatry* 2015; **20**(12)**:** 1538-1545.

178. Chen H, Wang N, Zhao X, Ross CA, O'Shea KS, McInnis MG. Gene expression alterations in bipolar disorder postmortem brains. *Bipolar Disord* 2013; **15**(2)**:** 177-187.

179. Breen MS, Tylee DS, Maihofer AX, Neylan TC, Mehta D, Binder EB *et al.* PTSD Blood Transcriptome Mega-Analysis: Shared Inflammatory Pathways across Biological Sex and Modes of Trauma. *Neuropsychopharmacology* 2018; **43**(3)**:** 469-481.

180. Driessen TM, Eisinger BE, Zhao C, Stevenson SA, Saul MC, Gammie SC. Genes showing altered expression in the medial preoptic area in the highly social maternal phenotype are related to autism and other disorders with social deficits. *BMC Neurosci* 2014; **15:** 11.

181. Le-Niculescu H, Kurian SM, Yehyawi N, Dike C, Patel SD, Edenberg HJ *et al.* Identifying blood biomarkers for mood disorders using convergent functional genomics. *Mol Psychiatry* 2009; **14**(2)**:** 156-174.

182. Daskalakis NP, Cohen H, Cai G, Buxbaum JD, Yehuda R. Expression profiling associates blood and brain glucocorticoid receptor signaling with trauma-related individual differences in both sexes. *Proc Natl Acad Sci U S A* 2014; **111**(37)**:** 13529-13534.

183. Omrani MD, Bagheri M, Bushehri B, Azizi F, Anoshae MR. The association of TGF-beta1 codon 10 polymorphism with suicide behavior. *Am J Med Genet B Neuropsychiatr Genet* 2012; **159B**(7)**:** 772-775.

184. Chen Y, Xie W, Hu F, Chen J, Zheng H, Zhou H *et al.* Clinical diagnosis and mutation analysis of a Chinese family with Camurati-Engelmann disease. *Molecular medicine reports* 2017; **15**(1)**:** 235-239.

185. Carrieri G, Marzi E, Olivieri F, Marchegiani F, Cavallone L, Cardelli M *et al.* The G/C915 polymorphism of transforming growth factor beta1 is associated with human longevity: a study in Italian centenarians. *Aging Cell* 2004; **3**(6)**:** 443-448.

186. Cattaneo A, Cattane N, Malpighi C, Czamara D, Suarez A, Mariani N *et al.* FoxO1, A2M, and TGF-beta1: three novel genes predicting depression in gene X environment interactions are identified using cross-species and cross-tissues transcriptomic and miRNomic analyses. *Mol Psychiatry* 2018; **23**(11)**:** 2192-2208.

187. Amoli MM, Khatami F, Arzaghi SM, Enayati S, Nejatisafa AA. Over-expression of TGF-beta1 gene in medication free Schizophrenia. *Psychoneuroendocrinology* 2019; **99:** 265-270.

188. Borovcanin M, Jovanovic I, Radosavljevic G, Djukic Dejanovic S, Bankovic D, Arsenijevic N *et al.* Elevated serum level of type-2 cytokine and low IL-17 in first episode psychosis and schizophrenia in relapse. *J Psychiatr Res* 2012; **46**(11)**:** 1421-1426.

189. Bam M, Yang X, Zumbrun EE, Ginsberg JP, Leyden Q, Zhang J *et al.* Decreased AGO2 and DCR1 in PBMCs from War Veterans with PTSD leads to diminished miRNA resulting in elevated inflammation. *Transl Psychiatry* 2017; **7**(8)**:** e1222.

190. Neylan TC, Sun B, Rempel H, Ross J, Lenoci M, O'Donovan A *et al.* Suppressed monocyte gene expression profile in men versus women with PTSD. *Brain Behav Immun* 2011; **25**(3)**:** 524-531.

191. Segman RH, Shefi N, Goltser-Dubner T, Friedman N, Kaminski N, Shalev AY. Peripheral blood mononuclear cell gene expression profiles identify emergent post-traumatic stress disorder among trauma survivors. *Mol Psychiatry* 2005; **10**(5)**:** 500-513, 425.

192. Gulsuner S, Walsh T, Watts AC, Lee MK, Thornton AM, Casadei S *et al.* Spatial and temporal mapping of de novo mutations in schizophrenia to a fetal prefrontal cortical network. *Cell* 2013; **154**(3)**:** 518-529.

193. Mistry M, Gillis J, Pavlidis P. Genome-wide expression profiling of schizophrenia using a large combined cohort. *Mol Psychiatry* 2013; **18**(2)**:** 215-225.

194. Worst TJ, Tan JC, Robertson DJ, Freeman WM, Hyytia P, Kiianmaa K *et al.* Transcriptome analysis of frontal cortex in alcohol-preferring and nonpreferring rats. *J Neurosci Res* 2005; **80**(4)**:** 529-538.

195. Lizano P, Lutz O, Ling G, Padmanabhan J, Tandon N, Sweeney J *et al.* VEGFA GENE variation influences hallucinations and frontotemporal morphology in psychotic disorders: a B-SNIP study. *Transl Psychiatry* 2018; **8**(1)**:** 215.

196. Han IB, Ropper AE, Teng YD, Shin DA, Jeon YJ, Park HM *et al.* Association between VEGF and eNOS gene polymorphisms and lumbar disc degeneration in a young Korean population. *Genetics and molecular research : GMR* 2013; **12**(3)**:** 2294-2305.

197. Xie T, Stathopoulou MG, de Andres F, Siest G, Murray H, Martin M *et al.* VEGF-related polymorphisms identified by GWAS and risk for major depression. *Transl Psychiatry* 2017; **7**(3)**:** e1055.

198. Davies G, Lam M, Harris SE, Trampush JW, Luciano M, Hill WD *et al.* Study of 300,486 individuals identifies 148 independent genetic loci influencing general cognitive function. *Nature communications* 2018; **9**(1)**:** 2098.

199. Katsuura S, Kamezaki Y, Yamagishi N, Kuwano Y, Nishida K, Masuda K *et al.* Circulating vascular endothelial growth factor is independently and negatively associated with trait anxiety and depressive mood in healthy Japanese university students. *Int J Psychophysiol* 2011; **81**(1)**:** 38-43.

200. Benes FM, Lim B, Subburaju S. Site-specific regulation of cell cycle and DNA repair in post-mitotic GABA cells in schizophrenic versus bipolars. *Proc Natl Acad Sci U S A* 2009; **106**(28)**:** 11731-11736.

201. Sheng G, Demers M, Subburaju S, Benes FM. Differences in the circuitry-based association of copy numbers and gene expression between the hippocampi of patients with schizophrenia and the hippocampi of patients with bipolar disorder. *Arch Gen Psychiatry* 2012; **69**(6)**:** 550-561.

202. Fulzele S, Pillai A. Decreased VEGF mRNA expression in the dorsolateral prefrontal cortex of schizophrenia subjects. *Schizophr Res* 2009; **115**(2-3)**:** 372-373.

203. Flory JD, Donohue D, Muhie S, Yang R, Miller SA, Hammamieh R *et al.* Gene expression associated with suicide attempts in US veterans. *Transl Psychiatry* 2017; **7**(9)**:** e1226.

204. Lee BH, Hong JP, Hwang JA, Ham BJ, Na KS, Kim WJ *et al.* Alterations in plasma vascular endothelial growth factor levels in patients with schizophrenia before and after treatment. *Psychiatry Res* 2015; **228**(1)**:** 95-99.

205. Isung J, Mobarrez F, Nordstrom P, Asberg M, Jokinen J. Low plasma vascular endothelial growth factor (VEGF) associated with completed suicide. *World J Biol Psychiatry* 2012; **13**(6)**:** 468-473.

206. Hodes GE, Pfau ML, Purushothaman I, Ahn HF, Golden SA, Christoffel DJ *et al.* Sex Differences in Nucleus Accumbens Transcriptome Profiles Associated with Susceptibility versus Resilience to Subchronic Variable Stress. *J Neurosci* 2015; **35**(50)**:** 16362-16376.

207. Aalling N, Hageman I, Miskowiak K, Orlowski D, Wegener G, Wortwein G. Erythropoietin prevents the effect of chronic restraint stress on the number of hippocampal CA3c dendritic terminals-relation to expression of genes involved in synaptic plasticity, angiogenesis, inflammation, and oxidative stress in male rats. *J Neurosci Res* 2018; **96**(1)**:** 103-116.

208. Kuan PF, Waszczuk MA, Kotov R, Clouston S, Yang X, Singh PK *et al.* Gene expression associated with PTSD in World Trade Center responders: An RNA sequencing study. *Translational psychiatry* 2017; **7**(12)**:** 1297.

209. Dunn EC, Wiste A, Radmanesh F, Almli LM, Gogarten SM, Sofer T *et al.* Genome-Wide Association Study (Gwas) and Genome-Wide by Environment Interaction Study (Gweis) of Depressive Symptoms in African American and Hispanic/Latina Women. *Depress Anxiety* 2016; **33**(4)**:** 265-280.

210. Szatkiewicz JP, O'Dushlaine C, Chen G, Chambert K, Moran JL, Neale BM *et al.* Copy number variation in schizophrenia in Sweden. *Mol Psychiatry* 2014; **19**(7)**:** 762-773.

211. Fernandez-Castillo N, Cabana-Dominguez J, Soriano J, Sanchez-Mora C, Roncero C, Grau-Lopez L *et al.* Transcriptomic and genetic studies identify NFAT5 as a candidate gene for cocaine dependence. *Transl Psychiatry* 2015; **5:** e667.

212. Harris SE, Riggio V, Evenden L, Gilchrist T, McCafferty S, Murphy L *et al.* Age-related gene expression changes, and transcriptome wide association study of physical and cognitive aging traits, in the Lothian Birth Cohort 1936. *Aging* 2017; **9**(12)**:** 2489-2503.

213. Beech RD, Lowthert L, Leffert JJ, Mason PN, Taylor MM, Umlauf S *et al.* Increased peripheral blood expression of electron transport chain genes in bipolar depression. *Bipolar Disord* 2010; **12**(8)**:** 813-824.

214. McBride WJ, Kimpel MW, McClintick JN, Ding ZM, Hauser SR, Edenberg HJ *et al.* Changes in gene expression within the ventral tegmental area following repeated excessive binge-like alcohol drinking by alcohol-preferring (P) rats. *Alcohol* 2013; **47**(5)**:** 367-380.

215. Jiang CH, Tsien JZ, Schultz PG, Hu Y. The effects of aging on gene expression in the hypothalamus and cortex of mice. *Proc Natl Acad Sci U S A* 2001; **98**(4)**:** 1930-1934.

216. Boks MP, Hoogendoorn M, Jungerius BJ, Bakker SC, Sommer IE, Sinke RJ *et al.* Do mood symptoms subdivide the schizophrenia phenotype? Association of the GMP6A gene with a depression subgroup. *Am J Med Genet B Neuropsychiatr Genet* 2008; **147B**(6)**:** 707-711.

217. Lencz T, Lambert C, DeRosse P, Burdick KE, Morgan TV, Kane JM *et al.* Runs of homozygosity reveal highly penetrant recessive loci in schizophrenia. *Proceedings of the National Academy of Sciences of the United States of America* 2007; **104**(50)**:** 19942-19947.

218. Sanchez-Roige S, Palmer AA, Fontanillas P, Elson SL, andMe Research T, Substance Use Disorder Working Group of the Psychiatric Genomics C *et al.* Genome-Wide Association Study Meta-Analysis of the Alcohol Use Disorders Identification Test (AUDIT) in Two Population-Based Cohorts. *Am J Psychiatry* 2018**:** appiajp201818040369.

219. Voutetakis K, Chatziioannou A, Gonos ES, Trougakos IP. Comparative Meta-Analysis of Transcriptomics Data during Cellular Senescence and In Vivo Tissue Ageing. *Oxidative medicine and cellular longevity* 2015; **2015:** 732914.

220. Aston C, Jiang L, Sokolov BP. Transcriptional profiling reveals evidence for signaling and oligodendroglial abnormalities in the temporal cortex from patients with major depressive disorder. *Mol Psychiatry* 2005; **10**(3)**:** 309-322.

221. Glatt SJ, Everall IP, Kremen WS, Corbeil J, Sasik R, Khanlou N *et al.* Comparative gene expression analysis of blood and brain provides concurrent validation of SELENBP1 up-regulation in schizophrenia. *Proc Natl Acad Sci U S A* 2005; **102**(43)**:** 15533-15538.

222. Ogden CA, Rich ME, Schork NJ, Paulus MP, Geyer MA, Lohr JB *et al.* Candidate genes, pathways and mechanisms for bipolar (manic-depressive) and related disorders: an expanded convergent functional genomics approach. *Mol Psychiatry* 2004; **9**(11)**:** 1007-1029.

223. Strauss J, McGregor S, Freeman N, Tiwari A, George CJ, Kovacs M *et al.* Association study of early-immediate genes in childhood-onset mood disorders and suicide attempt. *Psychiatry Res* 2012; **197**(1-2)**:** 49-54.

224. Kimoto S, Zaki MM, Bazmi HH, Lewis DA. Altered Markers of Cortical gamma-Aminobutyric Acid Neuronal Activity in Schizophrenia: Role of the NARP Gene. *JAMA Psychiatry* 2015; **72**(8)**:** 747-756.

225. Surget A, Wang Y, Leman S, Ibarguen-Vargas Y, Edgar N, Griebel G *et al.* Corticolimbic transcriptome changes are state-dependent and region-specific in a rodent model of depression and of antidepressant reversal. *Neuropsychopharmacology* 2009; **34**(6)**:** 1363-1380.

226. Levine ME, Crimmins EM. A Genetic Network Associated With Stress Resistance, Longevity, and Cancer in Humans. *J Gerontol A Biol Sci Med Sci* 2016; **71**(6)**:** 703-712.

227. Zhurov V, Stead JD, Merali Z, Palkovits M, Faludi G, Schild-Poulter C *et al.* Molecular pathway reconstruction and analysis of disturbed gene expression in depressed individuals who died by suicide. *PLoS ONE* 2012; **7**(10)**:** e47581.

228. Utge SJ, Soronen P, Loukola A, Kronholm E, Ollila HM, Pirkola S *et al.* Systematic analysis of circadian genes in a population-based sample reveals association of TIMELESS with depression and sleep disturbance. *PLoS ONE* 2010; **5**(2)**:** e9259.

229. McCarthy MJ, Welsh DK. Cellular circadian clocks in mood disorders. *Journal of biological rhythms* 2012; **27**(5)**:** 339-352.

230. Nicholas B, Rudrasingham V, Nash S, Kirov G, Owen MJ, Wimpory DC. Association of Per1 and Npas2 with autistic disorder: support for the clock genes/social timing hypothesis. *Mol Psychiatry* 2007; **12**(6)**:** 581-592.

231. Dong L, Bilbao A, Laucht M, Henriksson R, Yakovleva T, Ridinger M *et al.* Effects of the circadian rhythm gene period 1 (per1) on psychosocial stress-induced alcohol drinking. *Am J Psychiatry* 2011; **168**(10)**:** 1090-1098.

232. Li JZ, Bunney BG, Meng F, Hagenauer MH, Walsh DM, Vawter MP *et al.* Circadian patterns of gene expression in the human brain and disruption in major depressive disorder. *Proc Natl Acad Sci U S A* 2013; **110**(24)**:** 9950-9955.

233. Gonzalez R, Bernardo C, Cruz D, Walss-Bass C, Thompson PM. The relationships between clinical characteristics, alcohol and psychotropic exposure, and circadian gene expression in human postmortem samples of affective disorder and control subjects. *Psychiatry Res* 2014; **218**(3)**:** 359-362.

234. Sequeira A, Morgan L, Walsh DM, Cartagena PM, Choudary P, Li J *et al.* Gene expression changes in the prefrontal cortex, anterior cingulate cortex and nucleus accumbens of mood disorders subjects that committed suicide. *PLoS ONE* 2012; **7**(4)**:** e35367.

235. Aston C, Jiang L, Sokolov BP. Microarray analysis of postmortem temporal cortex from patients with schizophrenia. *J Neurosci Res* 2004; **77**(6)**:** 858-866.

236. Huang MC, Ho CW, Chen CH, Liu SC, Chen CC, Leu SJ. Reduced expression of circadian clock genes in male alcoholic patients. *Alcohol Clin Exp Res* 2010; **34**(11)**:** 1899-1904.

237. Middleton FA, Pato CN, Gentile KL, McGann L, Brown AM, Trauzzi M *et al.* Gene expression analysis of peripheral blood leukocytes from discordant sib-pairs with schizophrenia and bipolar disorder reveals points of convergence between genetic and functional genomic approaches. *Am J Med Genet B Neuropsychiatr Genet* 2005; **136B**(1)**:** 12-25.

238. Livingston WS, Rusch HL, Nersesian PV, Baxter T, Mysliwiec V, Gill JM. Improved Sleep in Military Personnel is Associated with Changes in the Expression of Inflammatory Genes and Improvement in Depression Symptoms. *Front Psychiatry* 2015; **6:** 59.

239. Guardado P, Olivera A, Rusch HL, Roy M, Martin C, Lejbman N *et al.* Altered gene expression of the innate immune, neuroendocrine, and nuclear factor-kappa B (NF-kappaB) systems is associated with posttraumatic stress disorder in military personnel. *Journal of anxiety disorders* 2016; **38:** 9-20.

240. Spencer S, Falcon E, Kumar J, Krishnan V, Mukherjee S, Birnbaum SG *et al.* Circadian genes Period 1 and Period 2 in the nucleus accumbens regulate anxiety-related behavior. *Eur J Neurosci* 2013; **37**(2)**:** 242-250.

241. Voleti B, Tanis KQ, Newton SS, Duman RS. Analysis of target genes regulated by chronic electroconvulsive therapy reveals role for Fzd6 in depression. *Biol Psychiatry* 2012; **71**(1)**:** 51-58.

242. Christiansen SL, Bouzinova EV, Fahrenkrug J, Wiborg O. Altered Expression Pattern of Clock Genes in a Rat Model of Depression. *Int J Neuropsychopharmacol* 2016; **19**(11).

243. Bremer A, Giacobini M, Eriksson M, Gustavsson P, Nordin V, Fernell E *et al.* Copy number variation characteristics in subpopulations of patients with autism spectrum disorders. *Am J Med Genet B Neuropsychiatr Genet* 2011; **156**(2)**:** 115-124.

244. International Schizophrenia C. Rare chromosomal deletions and duplications increase risk of schizophrenia. *Nature* 2008; **455**(7210)**:** 237-241.

245. Canli T, Wen R, Wang X, Mikhailik A, Yu L, Fleischman D *et al.* Differential transcriptome expression in human nucleus accumbens as a function of loneliness. *Mol Psychiatry* 2017; **22**(7)**:** 1069-1078.

246. Enwright Iii JF, Huo Z, Arion D, Corradi JP, Tseng G, Lewis DA. Transcriptome alterations of prefrontal cortical parvalbumin neurons in schizophrenia. *Mol Psychiatry* 2018; **23**(7)**:** 1606-1613.

247. Walther DM, Kasturi P, Zheng M, Pinkert S, Vecchi G, Ciryam P *et al.* Widespread Proteome Remodeling and Aggregation in Aging C. elegans. *Cell* 2015; **161**(4)**:** 919-932.

248. Eastwood SL, Harrison PJ. Synaptic pathology in the anterior cingulate cortex in schizophrenia and mood disorders. A review and a Western blot study of synaptophysin, GAP-43 and the complexins. *Brain Res Bull* 2001; **55**(5)**:** 569-578.

249. Tian SY, Wang JF, Bezchlibnyk YB, Young LT. Immunoreactivity of 43 kDa growth-associated protein is decreased in post mortem hippocampus of bipolar disorder and schizophrenia. *Neurosci Lett* 2007; **411**(2)**:** 123-127.

250. Torrey EF, Barci BM, Webster MJ, Bartko JJ, Meador-Woodruff JH, Knable MB. Neurochemical markers for schizophrenia, bipolar disorder, and major depression in postmortem brains. *Biol Psychiatry* 2005; **57**(3)**:** 252-260.

251. Focking M, Lopez LM, English JA, Dicker P, Wolff A, Brindley E *et al.* Proteomic and genomic evidence implicates the postsynaptic density in schizophrenia. *Mol Psychiatry* 2015; **20**(4)**:** 424-432.

252. Metz GA, Schwab ME. Behavioral characterization in a comprehensive mouse test battery reveals motor and sensory impairments in growth-associated protein-43 null mutant mice. *Neuroscience* 2004; **129**(3)**:** 563-574.

253. Eastwood SL, Lyon L, George L, Andrieux A, Job D, Harrison PJ. Altered expression of synaptic protein mRNAs in STOP (MAP6) mutant mice. *J Psychopharmacol* 2007; **21**(6)**:** 635-644.

254. Sommer JU, Schmitt A, Heck M, Schaeffer EL, Fendt M, Zink M *et al.* Differential expression of presynaptic genes in a rat model of postnatal hypoxia: relevance to schizophrenia. *Eur Arch Psychiatry Clin Neurosci* 2010; **260 Suppl 2:** S81-89.

255. Kim KH, Liu J, Sells Galvin RJ, Dage JL, Egeland JA, Smith RC *et al.* Transcriptomic Analysis of Induced Pluripotent Stem Cells Derived from Patients with Bipolar Disorder from an Old Order Amish Pedigree. *PLoS ONE* 2015; **10**(11)**:** e0142693.

256. Kurian SM, Le-Niculescu H, Patel SD, Bertram D, Davis J, Dike C *et al.* Identification of blood biomarkers for psychosis using convergent functional genomics. *Mol Psychiatry* 2011; **16**(1)**:** 37-58.

257. Lesscher HM, Houthuijzen JM, Groot Koerkamp MJ, Holstege FC, Vanderschuren LJ. Amygdala 14-3-3zeta as a novel modulator of escalating alcohol intake in mice. *PLoS ONE* 2012; **7**(5)**:** e37999.

258. Andrus BM, Blizinsky K, Vedell PT, Dennis K, Shukla PK, Schaffer DJ *et al.* Gene expression patterns in the hippocampus and amygdala of endogenous depression and chronic stress models. *Mol Psychiatry* 2012; **17**(1)**:** 49-61.

259. Sood S, Gallagher IJ, Lunnon K, Rullman E, Keohane A, Crossland H *et al.* A novel multi-tissue RNA diagnostic of healthy ageing relates to cognitive health status. *Genome Biol* 2015; **16:** 185.

260. Zapata I, Serpell JA, Alvarez CE. Genetic mapping of canine fear and aggression. *BMC Genomics* 2016; **17:** 572.

261. Jacobson ML, Kim LA, Patro R, Rosati B, McKinnon D. Common and differential transcriptional responses to different models of traumatic stress exposure in rats. *Transl Psychiatry* 2018; **8**(1)**:** 165.

262. Lewohl JM, Wang L, Miles MF, Zhang L, Dodd PR, Harris RA. Gene expression in human alcoholism: microarray analysis of frontal cortex. *Alcohol Clin Exp Res* 2000; **24**(12)**:** 1873-1882.

263. Mayfield RD, Lewohl JM, Dodd PR, Herlihy A, Liu J, Harris RA. Patterns of gene expression are altered in the frontal and motor cortices of human alcoholics. *J Neurochem* 2002; **81**(4)**:** 802-813.

264. Webster MJ, O'Grady J, Kleinman JE, Weickert CS. Glial fibrillary acidic protein mRNA levels in the cingulate cortex of individuals with depression, bipolar disorder and schizophrenia. *Neuroscience* 2005; **133**(2)**:** 453-461.

265. Tkachev D, Mimmack ML, Ryan MM, Wayland M, Freeman T, Jones PB *et al.* Oligodendrocyte dysfunction in schizophrenia and bipolar disorder. *Lancet* 2003; **362**(9386)**:** 798-805.

266. Chandley MJ, Szebeni K, Szebeni A, Crawford J, Stockmeier CA, Turecki G *et al.* Gene expression deficits in pontine locus coeruleus astrocytes in men with major depressive disorder. *J Psychiatry Neurosci* 2013; **38**(4)**:** 276-284.

267. Forero DA, Guio-Vega GP, Gonzalez-Giraldo Y. A comprehensive regional analysis of genome-wide expression profiles for major depressive disorder. *J Affect Disord* 2017; **218:** 86-92.

268. Fatemi SH, Laurence JA, Araghi-Niknam M, Stary JM, Schulz SC, Lee S *et al.* Glial fibrillary acidic protein is reduced in cerebellum of subjects with major depression, but not schizophrenia. *Schizophr Res* 2004; **69**(2-3)**:** 317-323.

269. Bernard R, Kerman IA, Thompson RC, Jones EG, Bunney WE, Barchas JD *et al.* Altered expression of glutamate signaling, growth factor, and glia genes in the locus coeruleus of patients with major depression. *Mol Psychiatry* 2011; **16**(6)**:** 634-646.

270. Torres-Platas SG, Nagy C, Wakid M, Turecki G, Mechawar N. Glial fibrillary acidic protein is differentially expressed across cortical and subcortical regions in healthy brains and downregulated in the thalamus and caudate nucleus of depressed suicides. *Mol Psychiatry* 2016; **21**(4)**:** 509-515.

271. Clark D, Dedova I, Cordwell S, Matsumoto I. A proteome analysis of the anterior cingulate cortex gray matter in schizophrenia. *Mol Psychiatry* 2006; **11**(5)**:** 459-470, 423.

272. Vawter MP, Barrett T, Cheadle C, Sokolov BP, Wood WH, 3rd, Donovan DM *et al.* Application of cDNA microarrays to examine gene expression differences in schizophrenia. *Brain Res Bull* 2001; **55**(5)**:** 641-650.

273. Matsuoka T, Tsunoda M, Sumiyoshi T, Takasaki I, Tabuchi Y, Seo T *et al.* Effect of MK-801 on gene expressions in the amygdala of rats. *Synapse* 2008; **62**(1)**:** 1-7.

274. Alonso E, Garrido E, Diez-Fernandez C, Perez-Garcia C, Herradon G, Ezquerra L *et al.* Yohimbine prevents morphine-induced changes of glial fibrillary acidic protein in brainstem and alpha2-adrenoceptor gene expression in hippocampus. *Neurosci Lett* 2007; **412**(2)**:** 163-167.

275. Banasr M, Chowdhury GM, Terwilliger R, Newton SS, Duman RS, Behar KL *et al.* Glial pathology in an animal model of depression: reversal of stress-induced cellular, metabolic and behavioral deficits by the glutamate-modulating drug riluzole. *Mol Psychiatry* 2010; **15**(5)**:** 501-511.

276. Kataoka M, Matoba N, Sawada T, Kazuno AA, Ishiwata M, Fujii K *et al.* Exome sequencing for bipolar disorder points to roles of de novo loss-of-function and protein-altering mutations. *Mol Psychiatry* 2016; **21**(7)**:** 885-893.

277. Barnes MR, Huxley-Jones J, Maycox PR, Lennon M, Thornber A, Kelly F *et al.* Transcription and pathway analysis of the superior temporal cortex and anterior prefrontal cortex in schizophrenia. *J Neurosci Res* 2011; **89**(8)**:** 1218-1227.

278. Tomita H, Ziegler ME, Kim HB, Evans SJ, Choudary PV, Li JZ *et al.* G protein-linked signaling pathways in bipolar and major depressive disorders. *Frontiers in genetics* 2013; **4:** 297.

279. Gottschalk MG, Wesseling H, Guest PC, Bahn S. Proteomic enrichment analysis of psychotic and affective disorders reveals common signatures in presynaptic glutamatergic signaling and energy metabolism. *Int J Neuropsychopharmacol* 2014; **18**(2).

280. Beech RD, Qu J, Leffert JJ, Lin A, Hong KA, Hansen J *et al.* Altered expression of cytokine signaling pathway genes in peripheral blood cells of alcohol dependent subjects: preliminary findings. *Alcohol Clin Exp Res* 2012; **36**(9)**:** 1487-1496.

281. Sokolowski M, Wasserman J, Wasserman D. Polygenic associations of neurodevelopmental genes in suicide attempt. *Mol Psychiatry* 2016; **21**(10)**:** 1381-1390.

282. Wheeler HE, Kim SK. Genetics and genomics of human ageing. *Philos Trans R Soc Lond B Biol Sci* 2011; **366**(1561)**:** 43-50.

283. Willcox BJ, Tranah GJ, Chen R, Morris BJ, Masaki KH, He Q *et al.* The FoxO3 gene and cause-specific mortality. *Aging Cell* 2016; **15**(4)**:** 617-624.

284. Chattarji S, Rao RP. Blood-brain biomarkers for stress susceptibility. *Proc Natl Acad Sci U S A* 2014; **111**(37)**:** 13253-13254.

285. Johnson MR, Shkura K, Langley SR, Delahaye-Duriez A, Srivastava P, Hill WD *et al.* Systems genetics identifies a convergent gene network for cognition and neurodevelopmental disease. *Nat Neurosci* 2016; **19**(2)**:** 223-232.

286. Druley TE, Wang L, Lin SJ, Lee JH, Zhang Q, Daw EW *et al.* Candidate gene resequencing to identify rare, pedigree-specific variants influencing healthy aging phenotypes in the long life family study. *BMC geriatrics* 2016; **16:** 80.

287. Szczepankiewicz A, Skibinska M, Hauser J, Slopien A, Leszczynska-Rodziewicz A, Kapelski P *et al.* Association analysis of the GSK-3beta T-50C gene polymorphism with schizophrenia and bipolar disorder. *Neuropsychobiology* 2006; **53**(1)**:** 51-56.

288. Lachman HM, Pedrosa E, Petruolo OA, Cockerham M, Papolos A, Novak T *et al.* Increase in GSK3beta gene copy number variation in bipolar disorder. *Am J Med Genet B Neuropsychiatr Genet* 2007; **144B**(3)**:** 259-265.

289. Jimenez E, Arias B, Mitjans M, Goikolea JM, Roda E, Ruiz V *et al.* Association between GSK3beta gene and increased impulsivity in bipolar disorder. *Eur Neuropsychopharmacol* 2014; **24**(4)**:** 510-518.

290. Lewis CM, Ng MY, Butler AW, Cohen-Woods S, Uher R, Pirlo K *et al.* Genome-wide association study of major recurrent depression in the U.K. population. *Am J Psychiatry* 2010; **167**(8)**:** 949-957.

291. Inkster B, Nichols TE, Saemann PG, Auer DP, Holsboer F, Muglia P *et al.* Association of GSK3beta polymorphisms with brain structural changes in major depressive disorder. *Arch Gen Psychiatry* 2009; **66**(7)**:** 721-728.

292. Kripke DF, Nievergelt CM, Tranah GJ, Murray SS, Rex KM, Grizas AP *et al.* FMR1, circadian genes and depression: suggestive associations or false discovery? *Journal of circadian rhythms* 2013; **11**(1)**:** 3.

293. Mozhui K, Wang X, Chen J, Mulligan MK, Li Z, Ingles J *et al.* Genetic regulation of Nrxn1 [corrected] expression: an integrative cross-species analysis of schizophrenia candidate genes. *Transl Psychiatry* 2011; **1:** e25.

294. Blasi G, Napolitano F, Ursini G, Di Giorgio A, Caforio G, Taurisano P *et al.* Association of GSK-3beta genetic variation with GSK-3beta expression, prefrontal cortical thickness, prefrontal physiology, and schizophrenia. *Am J Psychiatry* 2013; **170**(8)**:** 868-876.

295. Jimenez E, Arias B, Mitjans M, Goikolea JM, Roda E, Saiz PA *et al.* Genetic variability at IMPA2, INPP1 and GSK3beta increases the risk of suicidal behavior in bipolar patients. *Eur Neuropsychopharmacol* 2013; **23**(11)**:** 1452-1462.

296. Numata S, Ishii K, Tajima A, Iga J, Kinoshita M, Watanabe S *et al.* Blood diagnostic biomarkers for major depressive disorder using multiplex DNA methylation profiles: discovery and validation. *Epigenetics : official journal of the DNA Methylation Society* 2015; **10**(2)**:** 135-141.

297. Garbett KA, Vereczkei A, Kalman S, Wang L, Korade Z, Shelton RC *et al.* Fibroblasts from patients with major depressive disorder show distinct transcriptional response to metabolic stressors. *Transl Psychiatry* 2015; **5:** e523.

298. McLean CK, Narayan S, Lin SY, Rai N, Chung Y, Hipolito MS *et al.* Lithium-associated transcriptional regulation of CRMP1 in patient-derived olfactory neurons and symptom changes in bipolar disorder. *Transl Psychiatry* 2018; **8**(1)**:** 81.

299. Sommer W, Arlinde C, Heilig M. The search for candidate genes of alcoholism: evidence from expression profiling studies. *Addict Biol* 2005; **10**(1)**:** 71-79.

300. Wolen AR, Phillips CA, Langston MA, Putman AH, Vorster PJ, Bruce NA *et al.* Genetic dissection of acute ethanol responsive gene networks in prefrontal cortex: functional and mechanistic implications. *PloS one* 2012; **7**(4)**:** e33575.

301. Park SJ, Lee JY, Kim SJ, Choi SY, Yune TY, Ryu JH. Toll-like receptor-2 deficiency induces schizophrenia-like behaviors in mice. *Scientific reports* 2015; **5:** 8502.

302. Lavoie J, Hebert M, Beaulieu JM. Glycogen synthase kinase-3 overexpression replicates electroretinogram anomalies of offspring at high genetic risk for schizophrenia and bipolar disorder. *Biol Psychiatry* 2014; **76**(2)**:** 93-100.

303. Rotthier A, Baets J, De Vriendt E, Jacobs A, Auer-Grumbach M, Levy N *et al.* Genes for hereditary sensory and autonomic neuropathies: a genotype-phenotype correlation. *Brain* 2009; **132**(Pt 10)**:** 2699-2711.

304. Peddareddygari LR, Oberoi K, Vellore JR, Grewal RP. Factors Affecting Phenotype Variability in a Family with CMT2B: Gender and LRSAM1 Genotype. *Case reports in neurology* 2016; **8**(2)**:** 120-126.

305. Chan MK, Tsang TM, Harris LW, Guest PC, Holmes E, Bahn S. Evidence for disease and antipsychotic medication effects in post-mortem brain from schizophrenia patients. *Mol Psychiatry* 2011; **16**(12)**:** 1189-1202.

306. Janssens K, Goethals S, Atkinson D, Ermanoska B, Fransen E, Jordanova A *et al.* Human Rab7 mutation mimics features of Charcot-Marie-Tooth neuropathy type 2B in Drosophila. *Neurobiol Dis* 2014; **65:** 211-219.

307. Voineagu I, Wang X, Johnston P, Lowe JK, Tian Y, Horvath S *et al.* Transcriptomic analysis of autistic brain reveals convergent molecular pathology. *Nature* 2011; **474**(7351)**:** 380-384.

308. Tajouri L, Mellick AS, Tourtellotte A, Nagra RM, Griffiths LR. An examination of MS candidate genes identified as differentially regulated in multiple sclerosis plaque tissue, using absolute and comparative real-time Q-PCR analysis. *Brain Res Brain Res Protoc* 2005; **15**(2)**:** 79-91.

309. Miller BH, Zeier Z, Xi L, Lanz TA, Deng S, Strathmann J *et al.* MicroRNA-132 dysregulation in schizophrenia has implications for both neurodevelopment and adult brain function. *Proc Natl Acad Sci U S A* 2012; **109**(8)**:** 3125-3130.

310. Cao-Lei L, Massart R, Suderman MJ, Machnes Z, Elgbeili G, Laplante DP *et al.* DNA methylation signatures triggered by prenatal maternal stress exposure to a natural disaster: Project Ice Storm. *PLoS ONE* 2014; **9**(9)**:** e107653.

311. Hatzimanolis A, McGrath JA, Wang R, Li T, Wong PC, Nestadt G *et al.* Multiple variants aggregate in the neuregulin signaling pathway in a subset of schizophrenia patients. *Transl Psychiatry* 2013; **3:** e264.

312. Luciano M, Houlihan LM, Harris SE, Gow AJ, Hayward C, Starr JM *et al.* Association of existing and new candidate genes for anxiety, depression and personality traits in older people. *Behav Genet* 2010; **40**(4)**:** 518-532.

313. Coon H, Darlington TM, DiBlasi E, Callor WB, Ferris E, Fraser A *et al.* Genome-wide significant regions in 43 Utah high-risk families implicate multiple genes involved in risk for completed suicide. *Mol Psychiatry* 2018.

314. Williams FM, Scollen S, Cao D, Memari Y, Hyde CL, Zhang B *et al.* Genes contributing to pain sensitivity in the normal population: an exome sequencing study. *PLoS Genet* 2012; **8**(12)**:** e1003095.

315. Ouchi Y, Kubota Y, Kuramasu A, Watanabe T, Ito C. Gene expression profiling in whole cerebral cortices of phencyclidine- or methamphetamine-treated rats. *Brain Res Mol Brain Res* 2005; **140**(1-2)**:** 142-149.

316. Flatscher-Bader T, van der Brug M, Hwang JW, Gochee PA, Matsumoto I, Niwa S *et al.* Alcohol-responsive genes in the frontal cortex and nucleus accumbens of human alcoholics. *J Neurochem* 2005; **93**(2)**:** 359-370.

317. Yu H, Bi W, Liu C, Zhao Y, Zhang D, Yue W. A hypothesis-driven pathway analysis reveals myelin-related pathways that contribute to the risk of schizophrenia and bipolar disorder. *Prog Neuropsychopharmacol Biol Psychiatry* 2014; **51:** 140-145.

318. Le-Niculescu H, Case NJ, Hulvershorn L, Patel SD, Bowker D, Gupta J *et al.* Convergent functional genomic studies of omega-3 fatty acids in stress reactivity, bipolar disorder and alcoholism. *Transl Psychiatry* 2011; **1:** e4.

319. McCarthy MJ, Wei H, Marnoy Z, Darvish RM, McPhie DL, Cohen BM *et al.* Genetic and clinical factors predict lithium's effects on PER2 gene expression rhythms in cells from bipolar disorder patients. *Transl Psychiatry* 2013; **3:** e318.

320. Grieco SF, Velmeshev D, Magistri M, Eldar-Finkelman H, Faghihi MA, Jope RS *et al.* Ketamine up-regulates a cluster of intronic miRNAs within the serotonin receptor 2C gene by inhibiting glycogen synthase kinase-3. *The world journal of biological psychiatry : the official journal of the World Federation of Societies of Biological Psychiatry* 2017; **18**(6)**:** 445-456.

321. Fatemi SH, Reutiman TJ, Folsom TD. Chronic psychotropic drug treatment causes differential expression of Reelin signaling system in frontal cortex of rats. *Schizophr Res* 2009; **111**(1-3)**:** 138-152.

322. Herteleer L, Zwarts L, Hens K, Forero D, Del-Favero J, Callaerts P. Mood stabilizing drugs regulate transcription of immune, neuronal and metabolic pathway genes in Drosophila. *Psychopharmacology (Berl)* 2016; **233**(9)**:** 1751-1762.

323. Kv A, Madhana RM, Js IC, Lahkar M, Sinha S, Naidu VGM. Antidepressant activity of vorinostat is associated with amelioration of oxidative stress and inflammation in a corticosterone-induced chronic stress model in mice. *Behav Brain Res* 2018; **344:** 73-84.

324. Hammamieh R, Chakraborty N, Gautam A, Miller SA, Muhie S, Meyerhoff J *et al.* Transcriptomic analysis of the effects of a fish oil enriched diet on murine brains. *PLoS ONE* 2014; **9**(3)**:** e90425.

325. Squassina A, Costa M, Congiu D, Manchia M, Angius A, Deiana V *et al.* Insulin-like growth factor 1 (IGF-1) expression is up-regulated in lymphoblastoid cell lines of lithium responsive bipolar disorder patients. *Pharmacol Res* 2013; **73:** 1-7.

326. Khawaja X, Xu J, Liang JJ, Barrett JE. Proteomic analysis of protein changes developing in rat hippocampus after chronic antidepressant treatment: Implications for depressive disorders and future therapies. *J Neurosci Res* 2004; **75**(4)**:** 451-460.

327. Huang GJ, Ben-David E, Tort Piella A, Edwards A, Flint J, Shifman S. Neurogenomic evidence for a shared mechanism of the antidepressant effects of exercise and chronic fluoxetine in mice. *PLoS ONE* 2012; **7**(4)**:** e35901.

328. Benton CS, Miller BH, Skwerer S, Suzuki O, Schultz LE, Cameron MD *et al.* Evaluating genetic markers and neurobiochemical analytes for fluoxetine response using a panel of mouse inbred strains. *Psychopharmacology (Berl)* 2012; **221**(2)**:** 297-315.

329. Weber KT, Satoh S, Alipui DO, Virojanapa J, Levine M, Sison C *et al.* Exploratory study for identifying systemic biomarkers that correlate with pain response in patients with intervertebral disc disorders. *Immunologic research* 2015; **63**(1-3)**:** 170-180.

330. Yu Z, Ono C, Kim HB, Komatsu H, Tanabe Y, Sakae N *et al.* Four mood stabilizers commonly induce FEZ1 expression in human astrocytes. *Bipolar Disord* 2011; **13**(5-6)**:** 486-499.

331. Le-Niculescu H, Balaraman Y, Patel SD, Ayalew M, Gupta J, Kuczenski R *et al.* Convergent functional genomics of anxiety disorders: translational identification of genes, biomarkers, pathways and mechanisms. *Transl Psychiatry* 2011; **1:** e9.

332. Lagus M, Gass N, Saharinen J, Saarela J, Porkka-Heiskanen T, Paunio T. Gene expression patterns in a rodent model for depression. *Eur J Neurosci* 2010; **31**(8)**:** 1465-1473.

333. McQuillin A, Rizig M, Gurling HM. A microarray gene expression study of the molecular pharmacology of lithium carbonate on mouse brain mRNA to understand the neurobiology of mood stabilization and treatment of bipolar affective disorder. *Pharmacogenet Genomics* 2007; **17**(8)**:** 605-617.

334. Geoffroy PA, Curis E, Courtin C, Moreira J, Morvillers T, Etain B *et al.* Lithium response in bipolar disorders and core clock genes expression. *World J Biol Psychiatry* 2017**:** 1-14.

335. Moriya S, Tahara Y, Sasaki H, Hamaguchi Y, Kuriki D, Ishikawa R *et al.* Effect of quetiapine on Per1, Per2, and Bmal1 clock gene expression in the mouse amygdala and hippocampus. *J Pharmacol Sci* 2014; **125**(3)**:** 329-332.

336. Mahoney SJ, Narayan S, Molz L, Berstler LA, Kang SA, Vlasuk GP *et al.* A small molecule inhibitor of Rheb selectively targets mTORC1 signaling. *Nature communications* 2018; **9**(1)**:** 548.
